# Secondary Metabolites with *α*-Glucosidase Inhibitory Activity from Mangrove Endophytic Fungus *Talaromyces* sp. CY-3

**DOI:** 10.3390/md19090492

**Published:** 2021-08-28

**Authors:** Wencong Yang, Qi Tan, Yihao Yin, Yan Chen, Yi Zhang, Jianying Wu, Leyao Gao, Bo Wang, Zhigang She

**Affiliations:** 1 School of Chemistry, Sun Yat-Sen University, Guangzhou 510275, China; yangwc6@mail2.sysu.edu.cn (W.Y.); tanq27@mail2.sysu.edu.cn (Q.T.); yinyh6@mail2.sysu.edu.cn (Y.Y.); chenyan27@mail2.sysu.edu.cn (Y.C.); wujy89@mail2.sysu.edu.cn (J.W.); gaoly6@mail2.sysu.edu.cn (L.G.); 2 National R & D Center for Edible Fungus Processing Technology, Henan University, Kaifeng 475004, China; 3 Research Institute for Marine Drugs and Nutrition, College of Food Science and Technology, Guangdong Ocean University, Zhanjiang 524088, China; hubeizhangyi@163.com

**Keywords:** *Talaromyces* sp., molecular networking, sambutoxin, polyketides, *α*-glucosidase

## Abstract

Eight new compounds, including two sambutoxin derivatives (**1**–**2**), two highly oxygenated cyclopentenones (**7**–**8**), four highly oxygenated cyclohexenones (**9**–**12**), together with four known sambutoxin derivatives (**3**–**6**), were isolated from semimangrove endophytic fungus *Talaromyces* sp. CY-3, under the guidance of molecular networking. The structures of new isolates were elucidated by analysis of detailed spectroscopic data, ECD spectra, chemical hydrolysis, ^13^C NMR calculation, and DP4+ analysis. In bioassays, compounds **1**–**5** displayed better *α*-glucosidase inhibitory activity than the positive control 1-deoxynojirimycin (IC_50_ = 80.8 ± 0.3 μM), and the IC_50_ value was in the range of 12.6 ± 0.9 to 57.3 ± 1.3 μM.

## 1. Introduction

According to the WHO forecast, the number of diabetes patients will reach 693 million in 2045. Type II diabetes accounts for 90%, and *α*-glucosidase inhibitors originating from natural products, such as acarbose, miglitol, and voglibose, are used to treat type II diabetes [1]. However, most clinical antidiabetic drugs cause side effects [2]. Therefore, there is an urgent need to find and discover new antidiabetic drugs.

Mangrove endophytic fungi are an important resource to provide a large number of structurally unique secondary metabolites [3,4] with good biological activities, such as *α*-glucosidase inhibitory activities [5], antibacterial [6], antifungal [7], anti-insect [8], antitumor [9], antiviral [10], antioxidant [11], and anti-inflammatory activities [12]. As part of our ongoing search for new compounds with *α*-glucosidase inhibitory activities from mangrove-derived fungi [5,12,13,14], secondary metabolites of fungus *Talaromyces* sp. CY-3, collected from the fresh leaves of the semimangrove *Hibiscus tiliaceus* in Zhanjiang, were studied.

Recently, the advent of visual molecular network technology has led to a new perspective in the research of natural products [15]. Global Natural Product Social (GNPS) can establish a molecular network to classify compounds with the same LC–MS/MS ion fragments into similar clusters. Moreover, it can rapidly discover novel compounds through accurate MS data and database comparison [16].

**Figure 1:**
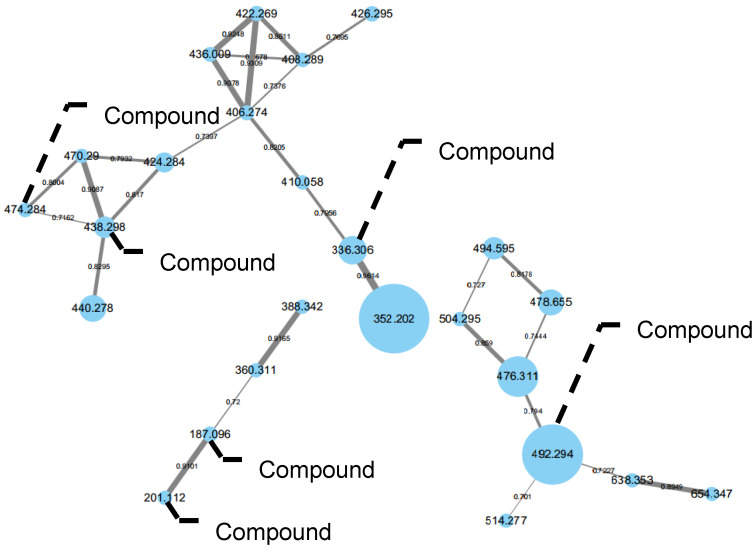
Clusters of nodes from *Talaromyces* sp. for compounds **1**–**3**, **5, 8**, and **10**–**12**.

**Figure 2:**
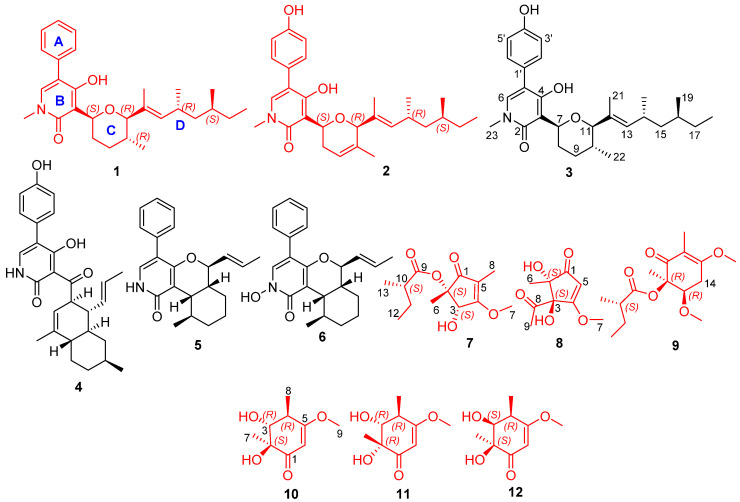
Structure of compounds **1**–**12**.

Extracts of CY-3 were analyzed by LC–MS/MS, and a visible molecular network was generated (Figure 1). Guided by MS/MS-based molecular networking through the GNPS platform, small clusters of compounds were tracked for isolation with *m*/*z* 492 [M+K]^+^, 336 [M+H]^+^, 438 [M+H]^+^, 474 [M+Na]^+^, 201 [M+H]^+^, and 187 [M+H]^+^. Two new sambutoxin derivatives (**1**–**2**), six highly oxygenated new polyketides (**7**–**12**), and four known sambutoxin derivatives (**3**–**6**) were isolated (Figure 2). Herein, the isolation, structure elucidation, and *α*-glucosidase inhibitory activity of all compounds are presented.

## 2. Results

### 2.1. Structure Identification

**Figure 3:**
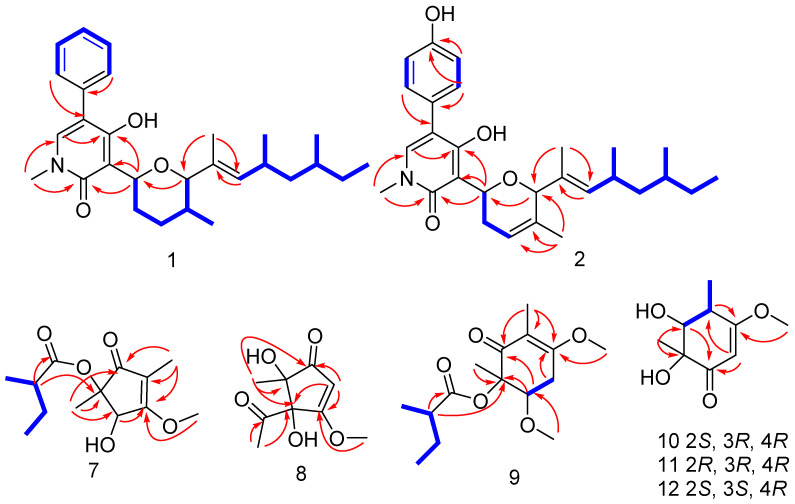
HMBC (red arrow) and key COSY (blue bold line) of **1**–**2** and **7**–**12**.

Sambutoxin A (**1**), obtained as a light-yellow oil, was displayed to have a molecular formula of C_28_H_37_NO_4_ with 10 degrees of unsaturation at *m*/*z* 438.2999 [M+H]^+^ (calcd. 438.3002), as shown by a positive HR–ESI–MS spectrum. The ^1^H NMR of **1** showed six methyls (*δ*_H_ 0.74, 0.82, 0.83, 0.90, 1.61, and 3.50), four methylenes (*δ*_H_ 1.03, 1.19, 1.34, 1.44, 1.64, 1.91, and 2.09), five methines (*δ*_H_ 1.30, 1.69, 2.45, 3.53, and 5.02), seven unsaturated protons (*δ*_H_ 5.18, 7.14, 7.32, 7.39, and 7.42), and one exchangeable hydrogen atom (*δ*_H_ 9.99). Its ^13^C NMR displayed a total of 28 carbons resonances, six methyls (*δ*_c_ 11.3, 11.7, 17.8, 19.7, 20.8, and 37.2), four methylenes (*δ*_c_ 29.0, 30.8, 32.2, and 44.8), five methines (*δ*_c_ 29.7, 32.1, 32.5, 78.0, and 92.7), seven carbons with unsaturated protons (*δ*_c_ 127.5, 128.4, 129.3, 136.4, and 138.1), and six quaternary carbons (*δ*_c_ 110.4, 115.2, 130.4, 134.2, 161.5, and 162.0). The HMBC from H-13 to C-12, from H-21 to C-13, together with the ^1^H-^1^H COSY H-13/H-14(/H-20)/H-15/H-16(/H-19)/H-17/H-18 formed the side chain D. HMBC from H-11 to C-7, together with ^1^H-^1^H COSY H-7/H-8/H-9/H-10(/H-22)/H-11, established the ring C moiety. HMBC from H-6 to C-2, C-4, and C-5 and from H-23 to C-2 and C-6, constructed a ring B moiety. ^1^H-^1^H COSY H-2′/H-3′/H-4′/H-5′/H-6′, together with HMBC from H-2′ to C-1′ and from H-6′ to C-1′, constructed ring A. Finally, rings A, B, C, and side chain D were connected by the HMBCs from H-6′ to C-5, H-7 to C-2, C-3, and C-4, and H-21 to C-11. The 1D and 2D NMR data were similar to those of **3** (Table 1). The only difference between them was that the 4′-OH (*δ*_C_ 156.5) is reduced to a hydrogen atom (*δ*_H_ 7.29–7.34, *δ*_C_ 127.5). Thus, the planar structure of **1** was shown (Figure 3). 

**Figure 4:**
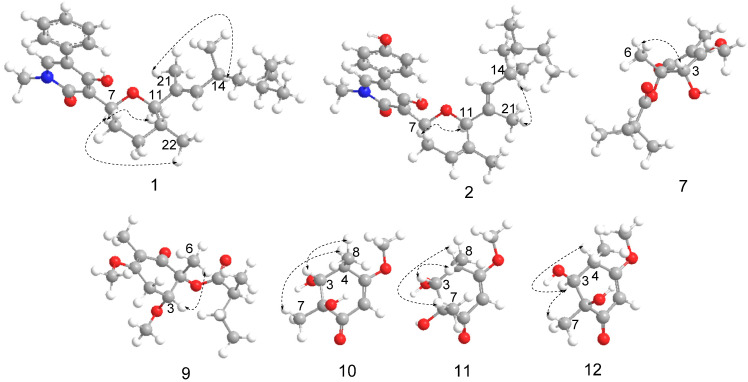
NOESY correlations of **1**–**2** and **7**–**12**.

**Figure 5:**
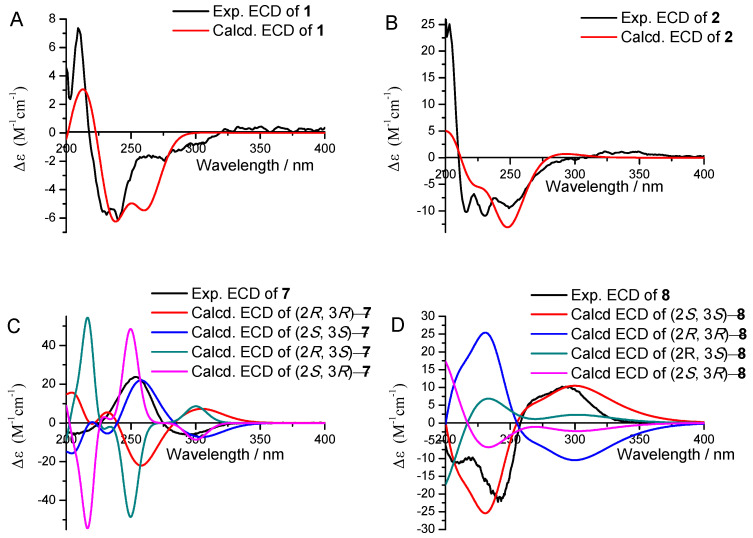

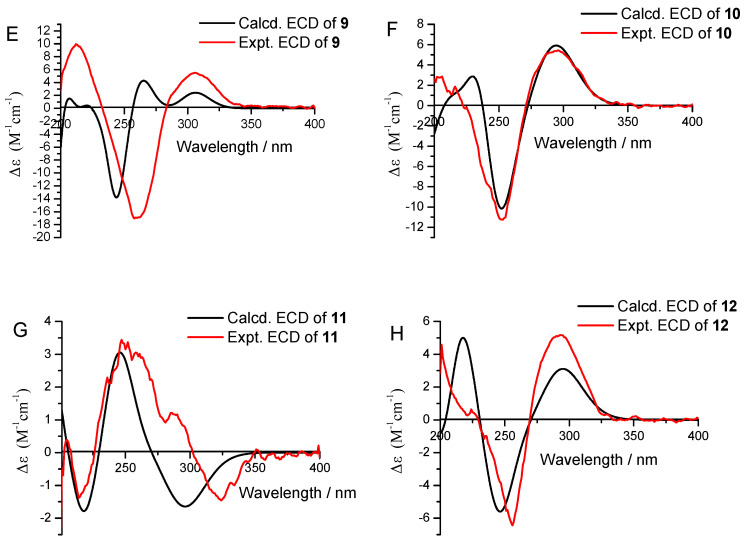
ECD spectra of compounds **1** (**A**), **2** (**B**), **7** (**C**), **8** (**D**), **9** (**E**), **10** (**F**), **11** (**G**) and **12** (**H**) in CH_3_OH.

The relative configuration of ring C and the double bond between C-12 and C-13 of compound **1** were defined by the NOESY spectrum. The correlations of H-22/H-7/H-11 and H-14/H-21 were also observed in the NOESY spectrum (Figure 4), which means H-22, H-7, and H-11 were on the same side, as were H-14 and H-21. Therefore, the relative configuration of C-7, C-10, and C-11 in ring C was deduced to be (7S*, 10R*, 11R*), and the double bond between C-12 and C-13 was assigned to be (*E*). In the present work, assigning the configurations at C-14 and C-16 in the aliphatic side chains of **1** was a challenging task due to the high conformational flexibility of fatty chains. As described in the literature, the absolute side-chain configurations for C-14 and C-16 were also determined by ^13^C NMR calculation and DP4+ analysis [17,18]. (14*R*, 16*S*)-**1** was assigned with a 100% probability (Figures S68 and S69). Consequently, the side-chain configurations of C-14 and C-16 of **1** were assigned to be 14*R*, 16*S*. The absolute configuration of **1** was determined by comparing the calculated ECD spectra (7*S*, 10*R*, 11*R,* 14*R*, 16*S*)-**1** and (7*R*, 10*S*, 11*S,* 14*R*, 16*S*)-**1** with the experimental one. The calculated ECD curves (7*S*, 10*R*, 11*R*, 14*R*, 16*S*)-**1** showed better agreement with the experimental one (Figure 5A). Thus, the absolute configuration of **1** was assigned to be 7*S*, 10*R*, 11*R*, 13*E*, 14*R*, 16*S*. Compounds **1** and **3** share a common biosynthetic pathway for sambutoxin derivatives, which is consistent with the configuration reported in the literature [19,20].

Sambutoxin B (**2**), also isolated as a light-yellow oil, displayed a molecular formula of C_28_H_37_NO_4_ with 11 degrees of unsaturation at *m*/*z* 452.2794 [M+H]^+^ (calcd. 452.2795) by positive HR–ESI–MS spectrum. Its 1D and 2D NMR were similar to **3**. The only difference between them was that the single bond between C-9 (*δ*_H_ 1.44, 1.97, *δ*_C_ 32.2) and C-10 (*δ*_H_ 1.64–1.70, *δ*_C_ 32.1) was converted to a double bond (*δ*_H_ 5.72, *δ*_C_ 121.9, 132.9). Thus, the planar structure of **2** was as shown in Figure 3. The correlations of H-7/H-11 and H-14/H-21 were observed in the NOESY spectrum (Figure 4). Thus, the double bond between C-12 and C-13 was assigned to be (*E*), and the relative configuration was deduced to be (7S*, 11R*). The calculated ECD spectra of (7*S*, 11*R*)-**2** and (7*R*, 11*S*)-**2** were compared to the measured one, and the calculated ECD curve of (7*S*, 11*R*)-**2** was showed a good agreement with the experimental one (Figure 5B). The stereochemistry of C-14 and C-16 was biogenetically established as 14*R*, 16*S*. Moreover, they were also verified by ^13^C NMR calculation and DP4+ probability (Figures S70 and S71). Thus, the absolute configuration of **2** was determined as 7*S*, 11*R*, 13*E*, 14*R*, 16*S*.

**Figure 6:**
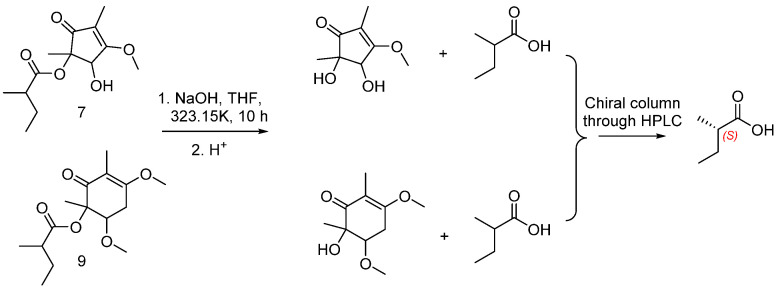
Confirmation for C-10 of compounds **7** and **9** through chemical hydrolysis.

Talaketides A(**7**) was isolated as a yellow oil, and its molecular formula was determined as C_13_H_20_O_5_ by the HR–ESI–MS data at *m*/*z* 279.1197 [M+Na]^+^ (calcd. 279.1203). The ^1^H NMR of **7** displayed four methyls, one methylene, two methines, and one methoxy (Table 2). Its ^13^C NMR showed a total of 13 carbons resonances, including four methyls, one methylene, two methines, one methoxy, one ester carboxyl, one carbonyl, two olefinic carbons, and one quaternary carbon (Table 3). The HMBC from H-3 to C-4, H-6 to C-1, C-2, and C-3, H-7 to C-4, H-8 to C-1, C-4, and C-5, H-10 to C-2 and C-9, together with the ^1^H-^1^H COSY correlation from H-13/H-10/H-11/H-12, formed the planar structure. In order to determine the absolute ring configuration of **7**, the ECD of (2*S*, 3*S*)-**7**, (2*R*, 3*R*)-**7**, (2*S*, 3*R*)-**7**, and (2*R*, 3*S*)-**7** was compared with the measured one. (2*S*, 3*S*)-**7** showed a good agreement with the experimental one (Figure 5C). Furthermore, the side chain of **7** was hydrolysis in 1M NaOH solution and compared to the standard of (*S*)-2-methylbutanoic acid and (*R*)-2-methylbutanoic acid through a chiral column by HPLC (Figure 6 and Figure S72). The retention time of hydrolyzate matched with (*S*)-2-methylbutanoic acid. Thus, the absolute configuration of **7** was determined as 2*S*, 3*S*, 10*S*.

Talaketides C (9) was isolated as a yellow oil, and its molecular formula was determined as C_15_H_24_O_5_ by the HR–ESI–MS data at *m*/*z* 307.1517 [M+Na]^+^ (calcd. 307.1516). The 1D and 2D NMR were also similar to **7**, and the only difference between them was compound **9** had one more methoxy group (*δ*_H_ 3.16, *δ*_c_ 51.8) and one more methylene group (*δ*_H_ 2.82, 3.03, *δ*_c_ 31.2) than compound **7**. According to the HMBCs, the methylene group was connected oxymethine (*δ*_H_ 5.10, *δ*_c_ 71.7) and a quaternary carbon (*δ*_c_ 188.2), and methoxy was connected to oxymethine (*δ*_H_ 5.10, *δ*_c_ 71.7), thus the planar structure of **9** was established (Figure 3). The NOESY correlation of H-5 to H-12 was observed (Figure 4), and the ECD curves of (2*R*, 3*R*) and (2*S*, 3*S*) were compared to the measured one. The calculated ECD of (2*R*, 3*R*) showed a good agreement with the experimental one (Figure 5E), and the side-chain stereostructure of C-10 was also determined as 10*S* through chemical hydrolysis. (Figure 6 and Figure S72). Therefore, the absolute configuration of **9** was determined as 2*R*, 3*R*, 10*S*.

Talaketides D (**10**) was isolated as a light-yellow oil, and its molecular formula was determined as C_9_H_14_O_4_ by the HR–ESI–MS data at *m*/*z* 209.0783 [M+Na]^+^ (calcd. 209.0784). Analysis of the 1D and 2D NMR spectrum found compound **10** to be similar to the known compound phomaligol D [21]. The only difference between them was the departure of hydroxyl at C-4 (*δ*_c_ 73.1 changed to *δ*_H_ 2.55, *δ*_c_ 41.5). Thus, the planar structure of **10** was determined (Figure 3), which was dehydroxylated phomaligol D. H-3/H-8 and H-4/H-7 correlations were observed in the NOESY spectrum, which indicated that the relative configuration of **10** was (2S*, 3R*, 4R*) (Figure 4). In order to determine its absolution configuration, the ECD spectra of (2*S*, 3*R*, 4*R*)-**10** and (2*R*, 3*S*, 4*S*)-**10** were compared with the measured one. The calculated CD curve of (2*S*, 3*R*, 4*R*) showed a good agreement with the experimental one (Figure 5F). Therefore, the absolution configuration of **10** was deduced to be (2*S*, 3*R*, 4*R*) and named Talaketides D.

Talaketides E (**11**) and Talaketides F (**12**), were also isolated as a light-yellow oil. Similarities in 1D and 2D NMR and HR–ESI–MS indicated that they shared the same planar structure (Figure 3), but the observed correlations were different: one was H-3/H-7/H-8, and the other was H-3/H-4/H-7 in the NOESY spectrum (Figure 4). This phenomenon indicated that their relative configuration was assigned to be (2R*, 3R*, 4R*) and (2S*, 3S*, 4R*), respectively. Their stereostructures were also determined by ECD calculation and comparison, and the absolution configuration of **11** and **12** was confirmed as (2*R*, 3*R*, 4*R*) and (2*S*, 3*S*, 4*R*), respectively (Figure 5G).

The structures of compounds **3**–**6** were identified as (–)-sambutoxin (**3**) [19], ilicicolin H (**4**) [22], deoxyleporin B (**5**) [23], and leporine B (**6**) [24], respectively, by comparison of their NMR data, MS, CD, and optical rotation with those in the literature.

### 2.2. Proposed Biosynthesis Pathway

**Figure 7:**
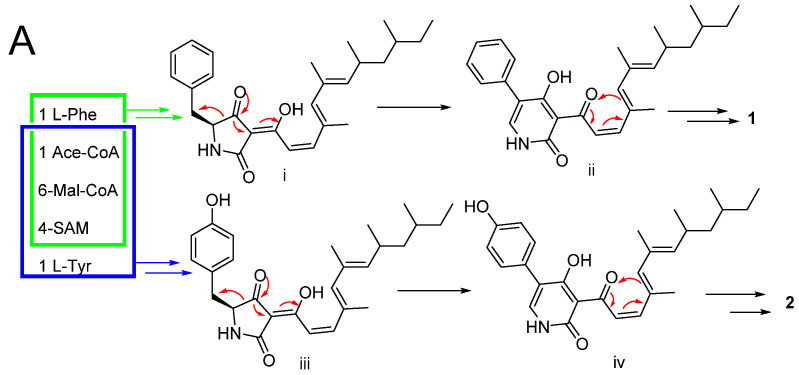

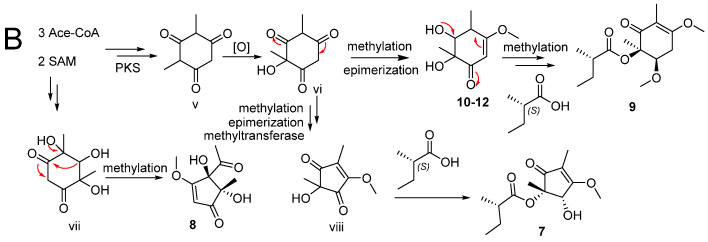
Proposed biogenetic pathways of **1**–**2** (**A**) and **7**–**12** (**B**).

A hypothetical biosynthetic pathway for compounds **1**–**2** and **7**–**12** was proposed (Figure 7) [25,26]. Compounds **1**–**2** are the PKS-NRPS biosynthetic pathway. Starting from one L-Phe molecule, one acetyl-CoA molecule, six malonyl-CoA molecules, and four SAM molecules through the PKS pathway formed intermediate i. Then, the formation of **1** was constructed by rearranging and reducing. The formation of **2** was similar to **1**, and the only difference between them was that L-Phe was replaced by L-Tyr (Figure 7A).

The remaining compounds **7**–**12** are considered to be the origin of biosynthetic polyketides. The key intermediate vi was obtained through the PKS pathway, oxidation, and formed **10**–**12** by electron transfer, methylation, and epimerization. Further electron transfer, methylation, and esterification to form **9**. **7** was formed through electron transfer, methylation, epimerization, methyltransferase, and esterification. Together, compound **8** started from two SAM molecules and three acetyl-CoA molecules through the PKS pathway and further rearranging and methylating (Figure 7B).

### 2.3. *α*-Glucosidase Inhibitory Activity

Compounds **1**–**12** were tested for their *α*-glucosidase inhibitory activity (Table 4). Compounds **1**–**5** displayed better *α*-glucosidase inhibitory activity with an IC_50_ value in the range of 12.6 ± 0.9 to 57.3 ± 1.3 μM compared to the positive control 1-deoxynojirimycin (IC_50_ = 80.8 ± 0.3 μM).

The IC_50_ value of compound **2** is 37.4 ± 1.4 μM, lower than that of compound **1** (12.6 ± 0.9 μM) and compound **3** (16.9 ± 0.6 μM), which illustrates that the double bond formed between C-9 and C-10 reduced the *α*-glucosidase inhibitory activity. Different from compounds **1**–**3**, compound **5** presented a much bigger IC_50_ value of 57.3 ± 1.3 μM, and the IC_50_ of compound **6** was even bigger than 100 μM. Therefore, it can be considered that the branch chain attached to ring C contributes a lot to the inhibitory activity. Compound **4** showed relatively higher inhibitory activity (IC_50_ = 16.5 ± 0.7 μM), which may be due to its different structure from the other five.

### 2.4. Molecular Docking Study

**Figure 8:**
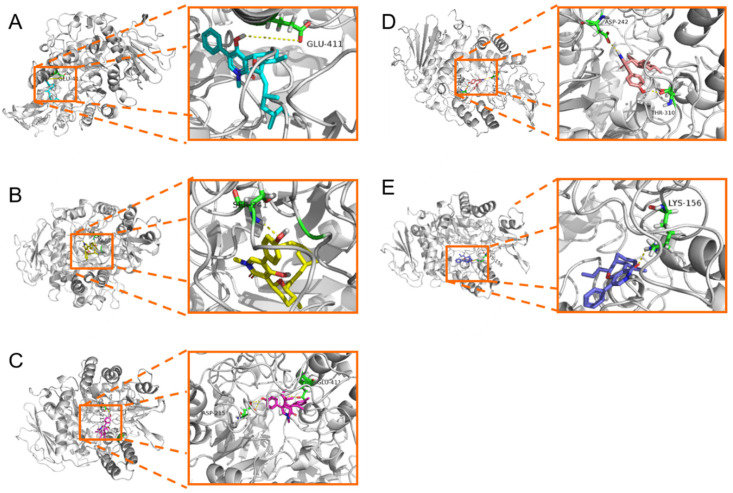
Binding mode of sambutoxins **1** (**A**), **2** (**B**), **3** (**C**), **4** (**D**), and **5** (**E**) with *α*-glucosidase.

To explain the difference in inhibitory activity of compounds **1**–**5** to *α*-glucosidase, molecular docking between them and *α*-glucosidase was carried out using Autodock. The interaction energies of compounds **1**–**5** with *α*-glucosidase were 8.25, 7.99, 8.85, 9.19, and 7.96 kcal/mol, respectively, which is consistent with the change in IC_50_ value. Compound **1** mainly formed a hydrogen bond with Glu-411 with a bond length of 2.85 Å (Figure 8A), **2** formed a hydrogen bond with Ser-241 with a bond length of 3.06 Å (Figure 8B), and **3** formed two hydrogen bonds with Asp-215 and Glu-411 with bond lengths of 3.06 and 2.85 Å, respectively. Moreover, compound **4** also formed two hydrogen bonds with Asp-242 and Thr-310 with bond lengths of 3.31 and 2.88 Å, respectively, while **5** only formed a hydrogen bond with Lys-156 with a bond length of 2.77 Å. In general, **1**, **3**, and **4** possess higher interaction energy and stronger interaction with amino acid residue than **2** and **5**, which explains their significant activity.

## 3. Experimental Section

### 3.1. General Experimental Procedures

The 1D and 2D NMR were recorded on a Bruker Avance 400 MHz spectrometer (Karlsruhe, Germany) at room temperature. HR–ESI–MS spectra of all test compounds were acquired on a ThermoFisher LTQ–Orbitrap–LC–MS spectrometer (Palo Alto, CA, USA). UV–vis spectra were measured on a Shimadzu UV–2600 spectrophotometer (Kyoto, Japan). Optical rotations were acquired on an Anton–Paar MCP500 automatic polarimeter at 25 ℃ (Graz, Austria). CD curves were recorded on an Applied Photophysics Chirascan spectropolarimeter (Surrey, UK). All spectrophotometric measurements used a 96-well Bio-Rad microplate reader (Hercules, CA, USA). Solvent was removed by a Heidolph rotavapor with a vacuum pump. Semipreparative HPLC chromatography was used on a U3000 separation module coupled with a DAD detector manufactured by ThermoFisher and a chiral semipreparative column (Nu-Analytical Solutions Co., LTD-packed chiral INB, 5 µm, 4.6 × 250 mm) was used for separation. Column chromatography (CC) used silica gel (200–300 mesh (Qingdao Marine Chemical Factory)) and Sephadex LH-20 (Amersham Pharmacia, Stockholm, Sweden). Precoated silica gel plates (Qingdao Huang Hai Chemical Group Co., G60, F-254) were used for TLC analysis. LC–MS analysis was performed on a Q-TOF manufactured by Waters and a Waters Acquity UPLC BEH C18 column (1.7 µm, 2.1 × 100 mm) was used for analysis.

### 3.2. Fungal Material

Fungus CY-3 was isolated from the fresh leaves of the semimangrove *Hibiscus tiliaceus* (collected in June 2020 from Zhanjiang Mangrove National Nature Reserve in Guangdong Province, China). It was identified as *Talaromyces* sp. using ITS gene sequencing. The ITS rDNA gene sequence data of the fungi were deposited to GenBank (Accession No. MZ614621), and fungus CY-3 was deposited at Sun Yat-Sen University, China.

### 3.3. Fermentation

CY-3 was activated on a potato dextrose agar (PDA) Petri dish at 28 ℃, then cultured in potato dextrose broth (PDB) in 6 × 500 mL Erlenmeyer flasks at 28 ℃ for 3 days in a shaker to obtain spore inoculum. The routine-scale fermentation was performed in 60 × 1 L Erlenmeyer flasks, each containing 50 mL of 2% saline and 50 g of rice. The Erlenmeyer flask containing the culture medium was autoclaved at 121 ℃ for 25 min. After cooling to room temperature, 10 mL of CY-3 inoculum was inoculated in each bottle and incubated at room temperature for 30 days.

### 3.4. Extraction and Purification

After fermentation, the mycelium and medium were extracted three times with EA (3 × 20 L). Then, the extracts were condensed under 50 ℃ in vacuo and combined to obtain a crude extract (31 g). The residue was separated by a silica gel column, eluting with a gradient of PE/EA (1:0–0:1) to afford 9 fractions (Frs. 1–8). Fr. 3 (2.4 g) was subjected to Sephadex LH-20 (methanol) to yield five sub-fractions (SFrs. 3.1–3.5). SFrs. 3–1 (352 mg) was applied to silica gel CC (DCM/MeOH *v*/*v*, 200:1) to give compounds **1** (5.3 mg), **2** (4.9 mg), and **3** (27.1 mg). Compounds **10** (6.3 mg, t_R_ = 7.3 min), **11** (5.7 mg, t_R_ = 9.1 min), and **12** (4.5 mg, t_R_ = 14.3 min) were obtained from SFr3-4 (298 mg) using chiral ND (the gradient was hexane/2-propanol *v*/*v*, 19:1, flow rate: 1 mL/min). Fr. 4 (2.5 g) was subjected to Sephadex LH-20 (ethanol) to yield three sub-fractions (SFrs. 4.1–4.3). Compounds **4** (8.7 mg), **5** (3.1 mg), and **6** (2.9 mg) were obtained from SFr4–1 (792 mg) using silica gel CC (DCM/MeOH *v*/*v*, 200:1). Fr. 6 (4.7 g) was subjected to Sephadex LH-20 (methanol) to yield three subfractions (SFrs. 6.1–6.3). Compounds **7** (50.1 mg), **8** (6.2 mg), and **9** (4.6 mg) were obtained from SFr6–3 (503 mg) using silica gel CC. (DCM/MeOH *v*/*v*, 50:1).

Sambutoxin A (**1**): C_28_H_39_NO_3_; yellow oil; ${\left[\alpha \right]}_{D}^{25}$ − 30.75° (*c* 0.74 MeOH); UV (MeOH) *λ*_max_ (log *ε*) 240 (1.95) nm; ECD (MeOH) *λ*_max_ (Δ*ε*) 210 (+2.65), 230 (−2.04), 243 (−2.03), 279 (−0.59) nm; ^1^H (400 MHz, CDCl_3_) and ^13^C NMR (100 MHz, CDCl_3_) data, see Table 1; HR–ESI–MS: *m*/*z* 438.2999 [M+H]^+^ (calcd. for C_28_H_40_NO_3_, 438.3002).

Sambutoxin B (**2**): C_28_H_37_NO_4_; yellow oil; ${\left[\alpha \right]}_{D}^{25}$ − 10.54° (*c* 1.05, MeOH); UV (MeOH) *λ*_max_ (log *ε*) 238 (2.86) nm; ECD (MeOH) *λ*_max_ (Δ*ε*) 217 (−3.45), 230 (−3.74), 259 (−3.16) nm; ^1^H (400 MHz, CDCl_3_) and ^13^C NMR (100 MHz, CDCl_3_) data, see Table 1; HR–ESI–MS: **m*/*z** 452.2794 [M+H]^+^ (calcd. for C_28_H_38_NO_4_, 452.2795).

Talaketides A (**7**): C_13_H_20_O_5_; yellow oil; ${\left[\alpha \right]}_{D}^{25}$ − 14.09° (*c* 0.26, MeOH); UV (MeOH) *λ*_max_ (log *ε*) 252 (2.70) nm; ECD (MeOH) *λ*_max_ (Δ*ε*) 211 (−1.52), 251 (+6.85), 301 (−2.68) nm; ^1^H (400 MHz, CDCl_3_) and ^13^C NMR (100 MHz, CDCl_3_) data, see Table 2 and Table 3; HR–ESI–MS: *m*/*z* 279.1197 [M+Na]^+^ (calcd. for C_13_H_20_O_5_Na, 279.1203).

Talaketides B (**8**): C_9_H_12_O_5_; yellow oil; ${\left[\alpha \right]}_{D}^{25}$ + 3.69° (*c* 0.24, MeOH); UV (MeOH) *λ*_max_ (log *ε*) 240 (3.28), 295 (2.53) nm; ECD (MeOH) *λ*_max_ (Δ*ε*) 208 (−2.95), 241 (−5.73), 294 (+2.65) nm; ^1^H (400 MHz, CDCl_3_) and ^13^C NMR (100 MHz, CDCl_3_) data, see Table 2 and Table 3; HR–ESI–MS: *m*/*z* 223.0574 [M+Na]^+^ (calcd. for C_9_H_12_O_5_Na, 233.0577).

Talaketides C (**9**): C_15_H_24_O_5_; yellow oil; ${\left[\alpha \right]}_{D}^{25}$ + 2.96° (*c* 1.12, MeOH); UV (MeOH) *λ*_max_ (log *ε*) 261 (2.94) nm; ECD (MeOH) *λ*_max_ (Δ*ε*) 205 (+0.36), 243 (−3.35), 262 (+0.97), 307 (+0.60) nm; ^1^H (400 MHz, CDCl_3_) and ^13^C NMR (100 MHz, CDCl_3_) data, see Table 2 and Table 3; HR–ESI–MS: *m*/*z* 307.1517 [M+Na]^+^ (calcd. for C_15_H_24_O_5_Na, 307.1516).

Talaketides D (**10**): C_9_H_14_O_4_; yellow oil; ${\left[\alpha \right]}_{D}^{25}$ + 16.04° (*c* 0.24, MeOH); UV (MeOH) *λ*_max_ (log *ε*) 251 (2.84) nm; ECD (MeOH) *λ*_max_ (Δ*ε*) 205 (+0.52), 252 (−2.08), 296 (+1.02) nm; ^1^H (400 MHz, CDCl_3_) and ^13^C NMR (100 MHz, CDCl_3_) data, see Table 2 and Table 3; HR–ESI–MS: *m*/*z* 209.0783 [M+Na]^+^ (calcd. for C_9_H_14_O_4_Na, 209.0784).

Talaketides E (**11**): C_9_H_14_O_4_; yellow oil; ${\left[\alpha \right]}_{D}^{25}$ − 11.35° (*c* 0.25, MeOH); UV (MeOH) *λ*_max_ (log *ε*) 252 (2.81) nm; ECD (MeOH) *λ*_max_ (Δ*ε*) 216 (−0.23), 249 (+0.60), 323 (−0.26) nm; ^1^H (400 MHz, CDCl_3_) and ^13^C NMR (100 MHz, CDCl_3_) data, see Table 2 and Table 3; HR–ESI–MS: *m*/*z* 209.0784 [M+Na]^+^ (calcd. for C_23_H_33_O_6_, 209.0784).

Talaketides F (**12**): C_19_H_25_ClO_5_; yellow oil; ${\left[\alpha \right]}_{D}^{25}$ + 49.14° (*c* 0.19, MeOH); UV (MeOH) *λ*_max_ (log *ε*) 252 (2.91) nm; ECD (MeOH) *λ*_max_ (Δ*ε*) 201 (+1.09), 256 (−1.52), 294 (+1.24) nm; ^1^H (400 MHz, CDCl_3_) and ^13^C NMR (100 MHz, CDCl_3_) data, see Table 2 and Table 3; HR–ESI–MS: *m*/*z* 369.1468 [M+H]^+^ (calcd. for C_19_H_26_ClO_5_, 369.1468).

### 3.5. Molecular Networking

The crude extract of CY-3 was analyzed by LC–MS/MS (LTQ Velos Pro-Orbitrap, Waltham, MA, USA) and a C_18_ column (Thermo Fisher Scientific-packed Hypersil GOLD, 1.9 µm, 2.1 × 100 mm). Samples were dissolved in MeCN at 1 mg/mL. A 10 μL of sample was injected and eluted with a gradient of H_2_O containing 0.1% HCOOH and MeCN containing 0.1% HCOOH with a gradient method as follows: 10% MeCN/H_2_O for 1 min, 10% MeCN/H_2_O to 60% in 9 min, 60% MeCN/H_2_O to 90% in 3 min, held at 90% MeCN/H_2_O for 3 min, then 90% MeCN/H_2_O to 10% MeCN/H_2_O in 0.2 min, and finally held at 10% MeCN/H_2_O for 3.8 min with the flow rate of 0.3 mL/min. Mass spectra were recorded in positive ESI mode (*m*/*z* 200–2000) and with an automated fully dependent MS/MS scan enabled. The molecular networking were made as described previously [27,28].

### 3.6. ECD and ^13^C NMR Calculations

ECD calculations and 13C NMR calculations were performed by the Gaussian 09 program and Spartan’14. The conformation with a Boltzmann population greater than 5% was selected for optimization and calculation in methanol at B3LYP/6-31+G (d, p). The ECD spectra were generated by the program SpecDis 1.6 (University of Würzburg, Würzburg, Germany) and drawn by OriginPro 8.0 (OriginLab, Ltd., Northampton, MA, USA) from dipole-length rotational strengths by applying Gaussian band shapes with sigma = 0.30 eV [29,30].

### 3.7. Bioassay

Compounds **1**–**12** were evaluated for *α*-glucosidase inhibitory activity, as described previously [31]. Three parallel concentrations of 1-deoxynojirimycin were taken as positive controls. DMSO was used as blank controls.

### 3.8. Molecular Docking

Because the crystal structure of glucosidase from *Saccharomyces cerevisiae* cannot be obtained, the α-glucosidase homology model (PDB:3AXH) provided by SWISSMODEL Repository was used, and the model quality was evaluated [29]. The α-glucosidase homology model (PDB:3AXH) with compounds **1**–**5** was performed on Autodock, as described previously [31,32,33,34].

## 4. Conclusions

In summary, two new sambutoxin derivatives (**1**–**2**) and six new highly oxygenated polyketides derivatives (**7**–**12**), together with four known compounds (**3**–**6**), were obtained using guidance through molecular networking from semimangrove endophytic fungus *Talaromyces* sp. CY-3. The structures of new isolates were elucidated by 1D and 2D NMR, HR–ESI–MS, ECD spectra, ^13^C NMR calculation, and DP4+ analysis, as well as chemical hydrolysis. The absolute configuration of sambutoxin derivatives (**1**–**2**) was determined through ^13^C NMR calculation and DP4+ analysis for the first time. In bioassays, compounds **1**–**5** displayed better *α*-glucosidase inhibitory activity with IC_50_ values in the range of 12.6 ± 0.9 to 57.3 ± 1.3 μM compared to the positive control 1-deoxynojirimycin (IC_50_ = 80.8 ± 0.3 μM).

## Figures and Tables

**Table 1:** ^1^H NMR and ^13^C NMR of **1**–**3**.

NO.	1 (CDCl_3_)		2 (CDCl_3_)		3 (CDCl_3_)	
	*δ*_C_, Type	*δ*_H_(*J* in Hz)	*δ*_C_, Type	*δ*_H_(*J* in Hz)	*δ*_C_, Type	*δ*_H_(*J* in Hz)
2	161.5, C		161.7, C		162.7, C	
3	110.4, C		110.1, C		110.4, C	
4	162.0, C		162.5, C		162.4, C	
5	115.2, C		115.4, C		115.9, C	
6	136.4, CH	7.14, s	136.2, CH	7.11, s	136.0, CH	7.12, s
7	78.0, CH	5.02, d, (9.1)	73.7, CH	5.17, dd, (10.5, 3.1)	77.8, CH	5.02, d, (9.0)
8	30.8, CH_2_	1.64–1.69, m, 2.09, d, (11.4)	29.9, CH_2_	1.25–1.31, m, 2.25–2.35, m	30.9, CH_2_	1.64–1.70, m, 2.09, d, (11.4)
9	32.3, CH_2_	1.44, d, (10.1) 1.91, d, (13.1)	121.9, CH	5.72, d, (5.2)	32.2, CH_2_	1.44, d, (10.1) 1.91, d, (13.1)
10	32.5, CH	1.64–1.69, m	132.9, C		32.1, CH	1.64–1.70, m
11	92.7, CH	3.53, s	86.9, CH	4.56, s	92.7, CH	3.53, s
12	130.4, C		130.2, C		130.3, C	
13	138.1, CH	5.18, d, (9.5)	139.7, CH	5.29, d, (9.6)	138.1, CH	5.18, d, (9.5)
14	29.7, CH	2.42–2.49, m	30.0, CH	2.40–2.50, m	29.7, CH	2.42–2.49, m
15	44.8, CH_2_	1.01–1.07, m 1.16–1.21, m	44.8, CH_2_	1.01–1.04, m, 1.25–1.31, m	44.8, CH_2_	1.01–1.07, m 1.16–1.21, m
16	32.1, CH	1.29–1.35, m	32.2, CH	1.25–1.31, m	32.4, CH	1.29–1.34, m
17	29.0, CH_2_	1.01–1.07, m 1.29–1.35, m	29.0, CH_2_	1.01–1.04, m, 1.25–1.31, m	29.0, CH_2_	1.01–1.07, m 1.29–1.34, m
18	11.3, CH_3_	0.83, s	11.0, CH_3_	1.53, d, (1.3)	11.3, CH_3_	0.83, s
19	19.7, CH_3_	0.82, d, (6.5)	20.8, CH_3_	0.90, d, (6.6)	19.7, CH_3_	0.82, d, (6.5)
20	20.8, CH_3_	0.90, d, (6.6)	19.7, CH_3_	0.82, d, (6.4)	20.8, CH_3_	0.90, d, (6.6)
21	11.7, CH_3_	1.61, s	11.3, CH_3_	1.49, s	11.7, CH_3_	1.61, s
22	17.8, CH_3_	0.74, d, (6.5)	19.1, CH_3_	0.81, s	17.7, CH_3_	0.74, d, (6.5)
23	37.2, CH_3_	3.50, s	37.3, CH_3_	3.50, s	37.4, CH_3_	3.50, s
1′	134.2, C		125.9, C		125.2, C	
2′	129.3, CH	7.35–7.44, m	130.6, CH	7.26, d, (9.0)	130.5, CH	7.26, d, (9.0)
3′	128.4, CH	7.35–7.44, m	115.5, CH	6.87, d, (8.1)	115.6, CH	6.92, d, (8.1)
4′	127.5, CH	7.29–7.34, m	155.8, C		156.5, C	
5′	128.4, CH	7.35–7.44, m	115.5, CH	6.87, d, (8.1)	115.6, CH	6.92, d, (8.1)
6′	129.3, CH	7.35–7.44, m	130.6, CH	7.26, d, (9.0)	130.5, CH	7.26, d, (9.0)
4′-OH		9.99, s		9.83, s		9.83, s

**Table 2:** ^1^H NMR of **7**–**12**.

Position	7	8	9	10	11	12
3	4.98, s		5.10, q, (6.3)	3.51, d, (9.5)	3.40, d, (7.2)	3.80, d, (3.2)
4				2.55, dqd, (11.3, 6.8, 1.7)	2.77–2.83, m	3.02, qdd, (7.1, 3.2, 1.9)
5		5.55, s				
6	1.30, s	1.42, s	1.29, d, (6.4)	5.30, d, (1.7)	5.33, d, (1.2)	5.34, d, (1.9)
7	4.16, s	3.98, s	4.10, s	1.28, d, (6.8)	1.30, d, (7.2)	1.27, d, (7.1)
8	1.67, s		1.56, t, (1.8)	1.22, s	1.34, s	1.30, s
9		2.20, s		3.75, s	3.76, s	3.75, s
10	2.37, q, (7.0)		2.19–2.28, m			
11	1.44–1.50, m 1.58–1.65, m		1.34–1.40, m 1.44–1.50, m			
12	0.94, t, (7.5)		0.80, t, (7.5)			
13	1.12, d, (7.0)		1.02, d, (7.0)			
14			2.82, dq, (18.2, 1.8) 3.03, dq, (18.2, 1.8)			
15			3.16, s			

**Table 3:** ^13^C NMR of **7**–**12**.

Position	7	8	9	10	11	12
1	202.0	200.6	205.0	203.0	200.9	203.3
2	86.4	80.4	84.3	78.4	75.5	77.7
3	73.5	92.5	71.7	78.1	78.7	79.0
4	179.7	192.4	188.2	41.5	40.1	39.0
5	115.6	103.3	117.5	180.1	180.5	178.9
6	19.5	23.5	14.9	99.5	99.6	99.0
7	59.1	60.1	58.2	14.0	14.2	13.4
8	6.08	207.2	5.6	19.2	21.4	23.6
9	177.5	27.5	176.4	57.2	57.0	57.1
10	42.0		42.4			
11	27.8		27.8			
12	11.8		11.7			
13	16.8		16.6			
14			31.2			
15			51.8			

**Table 4:** *α*-glucosidase inhibitory activity for **1**–**12**.

Compounds	IC_50_/μM	Compounds	IC_50_/μM
**1**	12.6 ± 0.9	**7**	>100
**2**	37.4 ± 1.4	**8**	>100
**3**	16.9 ± 0.6	**9**	>100
**4**	16.5 ± 0.7	**10**	>100
**5**	57.3 ± 1.3	**11**	>100
**6**	>100	**12**	>100
1-deoxynojirimycin	80.8 ± 0.3		

## Data Availability

Data are contained within the article and Supplementary Material.

## Supplementary Materials

The following are available online at https://www.mdpi.com/article/10.3390/md19090492/s1, Figure S1 LC–MS/MS of CY-3. Figure S2 ^1^H NMR of **1**. Figure S3 ^13^C NMR of **1**. Figure S4 DEPT 135 of **1**. Figure S5 HSQC spectrum of **1**. Figure S6 HMBC spectrum of **1**. Figure S7 COSY spectrum of **1**. Figure S8 NOESY spectrum of **1**. Figure S9 HR–ESI–MS spectrum of **1**. Figure S10 UV spectrum of **1**. Figure S11 ^1^H NMR of **2**. Figure S12 ^13^C NMR of **2**. Figure S13 DEPT 135 of **2**. Figure S14 HSQC spectrum of **2**. Figure S15 HMBC spectrum of **2**. Figure S16 COSY spectrum of **2**. Figure S17 NOESY spectrum of **2**. Figure S18 HR–ESI–MS spectrum of **2**. Figure S19 UV spectrum of **2**. Figure S20 ^1^H NMR of **7**. Figure S21 ^13^C NMR of **7**. Figure S22 HSQC spectrum of **7**. Figure S23 HMBC spectrum of **7**. Figure S24 COSY spectrum of **7**. Figure S25 NOESY spectrum of **7**. Figure S26 HR–ESI–MS spectrum of **7**. Figure S27 UV spectrum of **7**. Figure S28 ^1^H NMR of **8**. Figure S29 ^13^C NMR of **8**. Figure S30 HSQC spectrum of **8**. Figure S31 HMBC spectrum of **8**. Figure S32 COSY spectrum of **8**. Figure S33 NOESY spectrum of **8**. Figure S34 HR–ESI–MS spectrum of **8**. Figure S35 UV spectrum of **8**. Figure S36 ^1^H NMR of **9**. Figure S37 ^13^C NMR of **9**. Figure S38 HSQC spectrum of **9**. Figure S39 HMBC spectrum of **9**. Figure S40 COSY spectrum of **9**. Figure S41 NOESY spectrum of **9**. Figure S42 HR–ESI–MS spectrum of **9**. Figure S43 UV spectrum of **9**. Figure S44 ^1^H NMR of **10**. Figure S45 ^13^C NMR of **10**. Figure S46 HSQC spectrum of **10**. Figure S47 HMBC spectrum of **10**. Figure S48 COSY spectrum of **10**. Figure S49 NOESY spectrum of **10**. Figure S50 HR–ESI–MS spectrum of **10**. Figure S51 UV spectrum of **10**. Figure S52 ^1^H NMR of **11**. Figure S53 ^13^C NMR of **11**. Figure S54 HSQC spectrum of **11**. Figure S55 HMBC spectrum of **11**. Figure S56 COSY spectrum of **11**. Figure S57 NOESY spectrum of **11**. Figure S58 HR–ESI–MS spectrum of **11**. Figure S59 UV of **11**. Figure S60 ^1^H NMR of **12**. Figure S61 ^13^C NMR of **12**. Figure S62 HSQC spectrum of **12**. Figure S63 HMBC spectrum of **12**. Figure S64 COSY spectrum of **12**. Figure S65 NOESY spectrum of **12**. Figure S66 HR–ESI–MS spectrum of **12**. Figure S67 UV spectrum of **12**. Figure S68 Comparison of the experimental ^13^C NMR data of the 2,4,6-trimethyloct-2-ene side chain for compound **1** and the calculated chemical shifts for four 2,4,6-trimethyloct-2-ene side-chain diastereomers (14*R*,16*S*-*1*, 14*R*,16*R*-**1** 14*S*,16*R*-**1**, and 14*S*, 16*S*-**1**). Figure S69 DP4+ analysis of **1**. Figure S70 Comparison of the experimental ^13^C NMR data of the 2,4,6-trimethyloct-2-ene side chain for compound **2** and the calculated chemical shifts for four 2,4,6-trimethyloct-2-ene side-chain diastereomers (14*R*,16*S*-**2**, 14*R*,16*R*-**2** 14*S*,16*R*-**2**, and 14*S*, 16*S*-**2**). Figure S71 DP4+ analysis of **2**. Figure S72 Hydrolysis of **7** and **9** compared with the standard through HPLC chiral column.

## References

[1] Dhameja M., Gupta P. (2019). Synthetic heterocyclic candidates as promising *α*-glucosidase inhibitors: An overview. Eur. J. Med. Chem..

[2] Rajalakshmi R., Lalitha P., Sharma S.C., Rajiv A., Chithambharan A., Ponnusamy A. (2021). In Silico studies: Physicochemical properties, drug score, toxicity predictions and molecular docking of organosulphur compounds against *Diabetes mellitus*. J. Mol. Recognit..

[3] Carroll A.R., Coop B.R., Davis R.A., Keyzers R.A., Prinsep M.R. (2021). Marine natural products. Nat. Prod. Rep..

[4] Blunt J.W., Carroll A.R., Coop B.R., Davis R.A., Keyzers R.A., Prinsep M.R. (2018). Marine natural products. Nat. Prod. Rep..

[5] Liu Y.Y., Yang Q., Xia G.P., Huang H.B., Li H.X., Ma L., Lu Y.J., He L., Xia X.K., She Z.G. (2015). Polyketides with *α*-glucosidase inhibitory activity from a mangrove endophytic fungus, *Penicillium* sp. HN29-3B1. J. Nat. Prod..

[6] Chen S.H., Chen D.N., Cai R.L., Cui H., Long Y.H., Lu Y.J., Li C.Y., She Z.G. (2016). Cytotoxic and antibacterial preussomerins from the mangrove endophytic fungus *Lasiodiplodia theobromae* ZJ-HQ1. J. Nat. Prod..

[7] Liang Z.Y., Shen N.X., Zheng Y.Y., Wu J.T., Miao L., Fu X.M., Chen M., Wang C.Y. (2019). Two new unsaturated fatty acids from the mangrove rhizosphere soil-derived fungus *Penicillium javanicum* HK1-22. Bioorg. Chem..

[8] An C.Y., Li X.M., Li C.S., Wang M.H., Xu G.M., Wang B.G. (2013). Aniquinazolines A-D, four new quinazolinone alkaloids from marine-derived endophytic fungus *Aspergillus nidulans*. Mar. Drugs.

[9] Chen Y., Liu Z.M., Huang Y., Liu L., He J.G., Wang L., Yuan J., She Z.G. (2019). Ascomylactams A−C, cytotoxic 12- or 13-membered-ring macrocyclic alkaloids isolated from the mangrove endophytic fungus *Didymella* sp. CYSK-4, and structure eevisions of phomapyrrolidones A and C. J. Nat. Prod..

[10] Peng J.X., Lin T., Wang W., Xin Z.H., Zhu T.J., Gu Q.Q., Li D.H. (2013). Antiviral alkaloids produced by the mangrove-derived fungus *Cladosporium* sp. PJX-41. J. Nat. Prod..

[11] Zou G., Tan Q., Chen Y., Yang W.C., Zang Z.M., Jiang H.M., Chen S.Y., Wang B., She Z.G. (2021). Furobenzotropolones A, B and 3-hydroxyepicoccone B with antioxidative activity from mangrove endophytic fungus *Epicoccum nigrum* MLY-3. Mar. Drugs.

[12] Chen Y., Yang W.C., Zou G., Yan Z.Y., Qiu P., Long Y.H., She Z.G. (2020). Metabolites with anti-inflammatory and alpha-glucosidase inhibitory activities from the mangrove endophytic fungus *Phoma* sp. SYSU-SK-7. Tetrahedron Lett..

[13] Cai R.L., Wu Y.N., Chen S.H., Cui H., Liu Z.M., Li C.Y., She Z.G. (2018). Peniisocoumarins A–J: Isocoumarins from *Penicillium commune* QQF-3, an endophytic fungus of the mangrove plant *Kandelia candel*. J. Nat. Prod..

[14] Wu Y.N., Chen Y., Huang X.S., Pan Y.H., Liu Z.M., Yan T., Cao W.H., She Z.G. (2018). *α*-Glucosidase inhibitors: Diphenyl ethers and phenolic bisabolane sesquiterpenoids from the mangrove endophytic fungus *Aspergillus flavus* QQSG-3. Mar. Drugs.

[15] Allard P.M., Péresse T., Bisson J., Gindro K., Marcourt L., Pham V.C., Roussi F., Litaudon M., Wolfender J.L. (2016). Integration of molecular networking and in-silico MS/MS fragmentation for natural products dereplication. Anal. Chem..

[16] Nie Y.Y., Yang W.C., Liu Y.Y., Yang J.M., Lei X.L., Gerwick W.H., Zhang Y. (2020). Acetylcholinesterase inhibitors and antioxidants mining from marine fungi: Bioassays, bioactivity coupled LC–MS/MS analyses and molecular networking. Mar. Life Sci. Technol..

[17] Gu B., Wu Y., Tang J., Jiao W., Li L., Sun F., Wang S., Yang F., Lin H. (2018). Azaphilone and isocoumarin derivatives from the sponge-derived fungus *Eupenicillium* sp. 6A-9. Tetrahedron Lett..

[18] Huo C., Lu X., Zheng Z., Li Y., Xu Y., Zheng H., Niu Y. (2020). Azaphilones with protein tyrosine phosphatase inhibitory activity isolated from the fungus *Aspergillus deflectus*. Phytochemistry.

[19] Kim J.C., Lee Y.W., Tamura H., Yoshizawa T. (1995). Sambutoxin: A new mycotoxin isolated from *Fusarium sambucinum*. Tetrahedron Lett..

[20] Williams D.R., Turske R.A. (2000). Construction of 4-hydroxy-2-pyridinones. Total synthesis of (+)-sambutoxin. Org. Lett..

[21] Chunyu W.X., Zhao J.Y., Ding Z.G., Han X.L., Wang Y.X., Ding J.H., Wang F., Li M.G., Wen M.L. (2019). A new cyclohexenone from the tin mine tailingsderived fungus *Aspergillus flavus* YIM DT 10012. Nat. Prod. Res..

[22] Matsumoto M., Minato H. (1976). Structure of ilicicolin H, an antifungal antibiotic. Tetrahedron Lett..

[23] Sinder B.B., Lu Q. (1996). Total synthesis of (±)-leporin A. J. Org. Chem..

[24] Zhang C., Jin L., Mondie B., Mitchell S.S., Castelhano A.L., Cai W., Bergenhem N. (2003). Leporin B: A novel hexokinase II gene inducing agent from an unidentified fungus. Bioorg. Med. Chem. Lett..

[25] Zhang Z., Jamieson C.S., Zhao Y., Li D., Ohashi M., Houk K.N., Tang Y. (2019). Enzyme-catalyzed inverse-electron demand Diels-Alder reaction in the biosynthesis of antifungal ilicicolin H. J. Am. Chem. Soc..

[26] Millot M., Dieu A., Tomosi S. (2016). Dibenzofurans and derivatives from lichens and ascomycetes. Nat. Prod. Rep..

[27] Wang M., Carver J.J., Phelan V.V., Sanchez L.M., Garg N., Peng Y., Nguyen D.D., Watrous J., Kapono C.A., Luzzatto-Knaan T. (2016). Sharing and community curation of mass spectrometry data with Global Natural Products Social Molecular Networking. Nat. Biotechnol..

[28] Li Y., Yu H.B., Zhang Y., Leao T., Glukhov E., Pierce M.L., Zhang C., Kim H., Mao H.H., Fang F. (2020). Pagoamide A, a cyclic depsipeptide isolated from a cultured marine chlorophyte, *Derbesia* sp., using MS/MS-based molecular networking. J. Nat. Prod..

[29] Cui H., Liu Y.N., Li J., Huang X.S., Yan T., Cao W.H., Liu H.J., Long Y.H., She Z.G. (2018). Diaporindenes A–D: Four unusual 2,3-dihydro-1H-indene analogues with anti-inflammatory activities from the mangrove endophytic fungus *Diaporthe* sp. SYSU. J. Org. Chem..

[30] Frisch M.J., Trucks G.W., Schlegel H.B., Scuseria G.E., Robb M.A., Cheeseman J.R., Scalmani G., Barone V., Mennucci B., Petersson G.A. (2016). Gaussian 09.

[31] Ye G.J., Lan T., Huang Z.X., Cheng X.N., Cai C.Y., Ding S.M., Xie M.L., Wang B. (2019). Design and synthesis of novel xanthone-triazole derivatives aspotential antidiabetic agents:*α*-glucosidase inhibition and glucoseuptake promotion. Eur. J. Med. Chem..

[32] Chen T., Huang Y., Hong J., Wei X., Zeng F., Li J., Ye G., Yuan J., Long Y. (2021). Preparation, COX-2 inhibition and anticancer activity of sclerotiorin derivatives. Mar. Drugs.

[33] DeLano W.L. (2002). The PyMOL Molecular Graphics System. http://www.pymol.org.

[34] Wallace A.C., Laskowski R.A., Thornton J.M. (1995). LIGPLOT: A program to generate schematic diagrams of protein-ligand interactions. Protein Eng..

