# Supplementary material for: Secondary Metabolites with α-Glucosidase Inhibitory Activity from Mangrove Endophytic Fungus Talaromyces sp. CY-3
Source: Mar Drugs. 2021 Aug 28;19(9):492. doi: 10.3390/md19090492 (PMC8465095; doi:10.3390/md19090492)
Supplement: Supplementary file 1 [file marinedrugs-19-00492-s001.zip › marinedrugs-1354996-supplementary.pdf]

Secondary Metabolites with α-Glucosidase Inhibitory Activity from Mangrove Endophytic Fungus *Talaromyces* sp. CY-3

Wencong Yang[a], Qi Tan[a], Yihao Yin[a], Yan Chen[a,b], Yi Zhang[c], Jianying Wu[a], Leyao Gao[a], Bo Wang[a,*], Zhigang She[a,*]

[a] School of Chemistry, Sun Yat–Sen University, Guangzhou, 510275, P.R.China, e–mail:ceswb@mail.sysu.edu.cn (Bo Wang); cesshzhg@mail.sysu.edu.cn (Zhigang She)

[b] National R & D Center for Edible Fungus Processing Technology, Henan University, Kaifeng, 475004, P.R.China

[c] Research Institute for Marine Drugs and Nutrition, College of Food Science and Technology, Guangdong Ocean University, Zhanjiang 524088, P.R.China

ABSTRACT

Eight new compounds including two sambutoxin derivatives (**1**–**2**), two highly oxygenated cyclopentenones (**7**–**8**), four highly oxygenated cyclohexenones (**9**–**12**), together with four known sambutoxin derivatives (**3**–**6**), were isolated from semi-mangrove endophytic fungus *Talaromyces* sp. CY-3, under the guidance of molecular networking. The structures of new isolates were elucidated by analysis of the detailed spectroscopic data, ECD spectra, chemical hydrolysis, ^{13}C NMR calculation and DP4+ analysis. In bioassays, Compounds **1**–**5** displayed better α-glucosidase inhibitory activity than the positive control 1-deoxynojirimycin (IC_{50}=80.8±0.3 μM), the IC_{50} value were in the range of 12.6±0.9 to 57.3±1.3 μM.

Keywords *Talaromyces* sp.; molecular networking; sambutoxin; polyketides; α-glucosidase;

Contents

Figure S1 LC-MS/MS of CY-3……………………………………………………6

Figure S2 ^{1}H NMR of **1**…………………………………………………………6

Figure S3 ^{13}C NMR of **1**…………………………………………………………7

Figure S4 DEPT 135 of **1**……………………………………………………………7

Figure S5 HSQC spectrum of **1**……………………………………………………8

Figure S6 HMBC spectrum of **1**……………………………………………………8

Figure S7 COSY spectrum of **1**……………………………………………………9

Figure S8 NOESY spectrum of **1**…………………………………………………9

Figure S9 HR-ESI-MS spectrum of **1**……………………………………………10

Figure S10 UV spectrum of **1** and ECD spectra of **1** and **3**……………………10

Figure S11 ^{1}H NMR of **2**……………………………………………………..11

Figure S12 ^{13}C NMR of **2**……………………………………………………….11

Figure S13 DEPT 135 of **2**………………………………………………………….12

Figure S14 HSQC spectrum of **2**…………………………………………………12

Figure S15 HMBC spectrum of **2**…………………………………………………13

Figure S16 COSY spectrum of **2**…………………………………………………13

Figure S17 NOESY spectrum of **2**………………………………………………14

Figure S18 HR-ESI-MS spectrum of **2**…………………………………………14

Figure S19 UV spectrum of **2**……………………………………………………15

Figure S20 ^{1}H NMR of **7**……………………………………………………….15

Figure S21 ^{13}C NMR of **7**………………………………………………………16

Figure S22 HSQC spectrum of **7**…………………………………………………16

Figure S23 HMBC spectrum of **7**…………………………………………………17

Figure S24 COSY spectrum of **7**…………………………………………………17

Figure S25 NOESY spectrum of **7**………………………………………………18

Figure S26 HR-ESI-MS spectrum of **7**…………………………………………18

Figure S27 UV spectrum of **7**……………………………………………………..19

Figure S28 ^{1}H NMR of **8**……………………………………………………….19

Figure S29 ^{13}C NMR of **8**………………………………………………………20

Figure S30 HSQC spectrum of **8**……………………………………………………20

Figure S31 HMBC spectrum of **8**……………………………………………………21

Figure S32 COSY spectrum of **8**……………………………………………………21

Figure S33 NOESY spectrum of **8**…………………………………………………22

Figure S34 HR-ESI-MS spectrum of **8**……………………………………………22

Figure S35 UV spectrum of **8**………………………………………………………..23

Figure S36 ^{1}H NMR of **9**……………………………………………………………..23

Figure S37 ^{13}C NMR of **9**…………………………………………………………….24

Figure S38 HSQC spectrum of **9**……………………………………………………..24

Figure S39 HMBC spectrum of **9**……………………………………………………25

Figure S40 COSY spectrum of **9**……………………………………………………25

Figure S41 NOESY spectrum of **9**……………………………………………………26

Figure S42 HR-ESI-MS spectrum of **9**……………………………………………26

Figure S43 UV spectrum of **9**………………………………………………………..27

Figure S44 ^{1}H NMR of **10**………………………………………………………..27

Figure S45 ^{13}C NMR of **10**………………………………………………………..28

Figure S46 HSQC spectrum of **10**……………………………………………………28

Figure S47 HMBC spectrum of **10**…………………………………………………29

Figure S48 COSY spectrum of **10**……………………………………………………29

Figure S49 NOESY spectrum of **10**…………………………………………………30

Figure S50 HR-ESI-MS spectrum of **10**……………………………………………30

Figure S51 UV spectrum of **10**……………………………………………………..31

Figure S52 ^{1}H NMR of **11**…………………………………………………………….31

Figure S53 ^{13}C NMR of **11**…………………………………………………………32

Figure S54 HSQC spectrum of **11**……………………………………………………32

Figure S55 HMBC spectrum of **11**…………………………………………………33

Figure S56 COSY spectrum of **11**……………………………………………………33

Figure S57 NOESY spectrum of **11**…………………………………………………34

Figure S58 HR-ESI-MS spectrum of **11**……………………………………………34

Figure S59 UV of **11**……………………………………………………………………35

Figure S60 ^{1}H NMR of **12**……………………………………………………35
Figure S61 ^{13}C NMR of **12**……………………………………………………36
Figure S62 HSQC spectrum of **12**………………………………………………36
Figure S63 HMBC spectrum of **12**………………………………………………37
Figure S64 COSY spectrum of **12**………………………………………………37
Figure S65 NOESY spectrum of **12**………………………………………………38
Figure S66 HR-ESI-MS spectrum of **12**…………………………………………38
Figure S67 UV spectrum of **12**………………………………………………….39
Figure S68 Comparison of the experimental ^{13}C NMR data and mean absolute error of the 2,4,6-trimethyloct-2-ene side chain for compound **1** and the calculated chemical shifts for four 2,4,6-trimethyloct-2-ene side chain diastereomers (14*R*,16*S*-**1**, 14*R*,16*R*-**1** 14*S*,16*R*-**1**, and 14*S*, 16*S*-**1**). ……………………….…………………39
Figure S69 DP4+ analysis of **1**. …………………………………………………40
Figure S70 Comparison of the experimental ^{13}C NMR data and mean absolute error of the 2,4,6-trimethyloct-2-ene side chain for compound **2** and the calculated chemical shifts for four 2,4,6-trimethyloct-2-ene side chain diastereomers (14*R*,16*S*-**2**, 14*R*,16*R*-**2** 14*S*,16*R*-**2**, and 14*S*, 16*S*-**2**). ………………………..………………..40
Figure S71 DP4+ analysis of **2**. …………………………………………………41
Figure S72 Hydrolysis of **7** and **9** compared with the standard through HPLC chiral column. ……………………………………………………………………..41
Table S1 Gibbs free energies and Boltzmann-population of low-energy conformers of 7*S*, 10*R*, 11*R*, 13*E*, 14*R*, 16*S* -**1**. ………………………………………………41
Table S2 Cartesian coordinates for the low-energy optimized conformers of 7*S*, 10*R*, 11*R*, 13*E*, 14*R*, 16*S* -**1** at B3LYP/6-31+G (d, p) level of theory in MeOH…………42
Table S3 Gibbs free energies and Boltzmann-population of low-energy conformers of 7*S*, 11*R*, 13*E*, 14*R*, 16*S* -**2**. …………………………………………………….55
Table S4 Cartesian coordinates for the low-energy optimized conformers of 7*S*, 11*R*, 13*E*, 14*R*, 16*S* -**2** at B3LYP/6-31+G (d, p) level of theory in MeOH……………….55
Table S5 Gibbs free energies and Boltzmann-population of low-energy conformers of 2*R*, 3*R* -**7**. …………………………………………………………………73

Table S6 Cartesian coordinates for the low-energy optimized conformers of 2*R*, 3*R* **-7** at B3LYP/6-31+G (d, p) level of theory in MeOH…………………………………..74

Table S7 Gibbs free energies and Boltzmann-population of low-energy conformers of 2*S*, 3*R* **-7**. ……………………………………………………………………….84

Table S8 Cartesian coordinates for the low-energy optimized conformers of 2*S*, 3*R* **-7** at B3LYP/6-31+G (d, p) level of theory in MeOH. …………………………………85

Table S9 Gibbs free energies and Boltzmann-population of low-energy conformers of 2*S*, 3*S* **-8**. ………………………………………………………………………95

Table S10 Cartesian coordinates for the low-energy optimized conformers of 2*S*, 3*S* **-8** at B3LYP/6-31+G (d, p) level of theory in MeOH………………………………95

Table S11 Gibbs free energies and Boltzmann-population of low-energy conformers of 2*S*, 3*R* **-8**. …………………………………………………………………….99

Table S12 Cartesian coordinates for the low-energy optimized conformers of 2*S*, 3*R* **-8** at B3LYP/6-31+G (d, p) level of theory in MeOH…………………………….99

Table S13 Gibbs free energies and Boltzmann-population of low-energy conformers of 2*R*, 3*R* **-9**. …………………………………………………………………….100

Table S14 Cartesian coordinates for the low-energy optimized conformers of 2*R*, 3*R* **-9** at B3LYP/6-31+G (d, p) level of theory in MeOH…………………………….101

Table S15 Gibbs free energies and Boltzmann-population of low-energy conformers of 2*S*, 3*R*, 4*R* **-10**. ………………………………………………………….113

Table S16 Cartesian coordinates for the low-energy optimized conformers of 2*S*, 3*R*, 4*R* **-10** at B3LYP/6-31+G (d, p) level of theory in MeOH………………………..113

Table S17 Gibbs free energies and Boltzmann-population of low-energy conformers of 2*R*, 3*R*, 4*R* **-11**. …………………………………………………………..115

Table S18 Cartesian coordinates for the low-energy optimized conformers of 7*S*, 10*R*, 11*R*, 13*E*, 14*R*, 16*S* **-1** at B3LYP/6-31+G (d, p) level of theory in MeOH…………115

Table S19 Gibbs free energies and Boltzmann-population of low-energy conformers of 2*R*, 3*R*, 4*R* **-12**.. ………………………………………………………….117

Table S20 Cartesian coordinates for the low-energy optimized conformers of 2*R*, 3*R*, 4*R* **-12** at B3LYP/6-31+G (d, p) level of theory in MeOH………………………….117

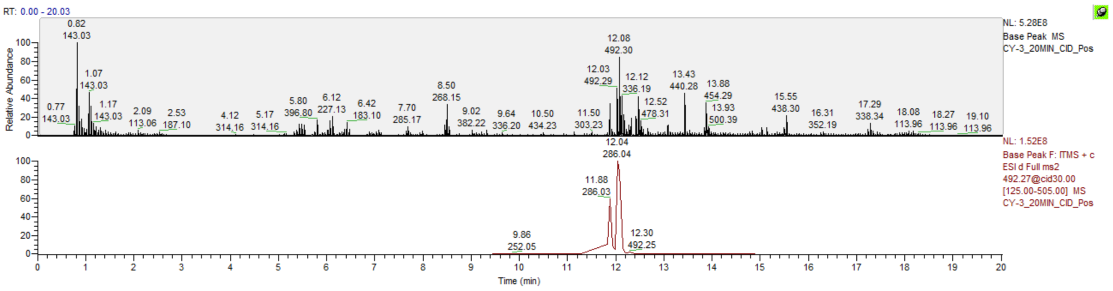

Figure S1 LC-MS/MS of CY-3

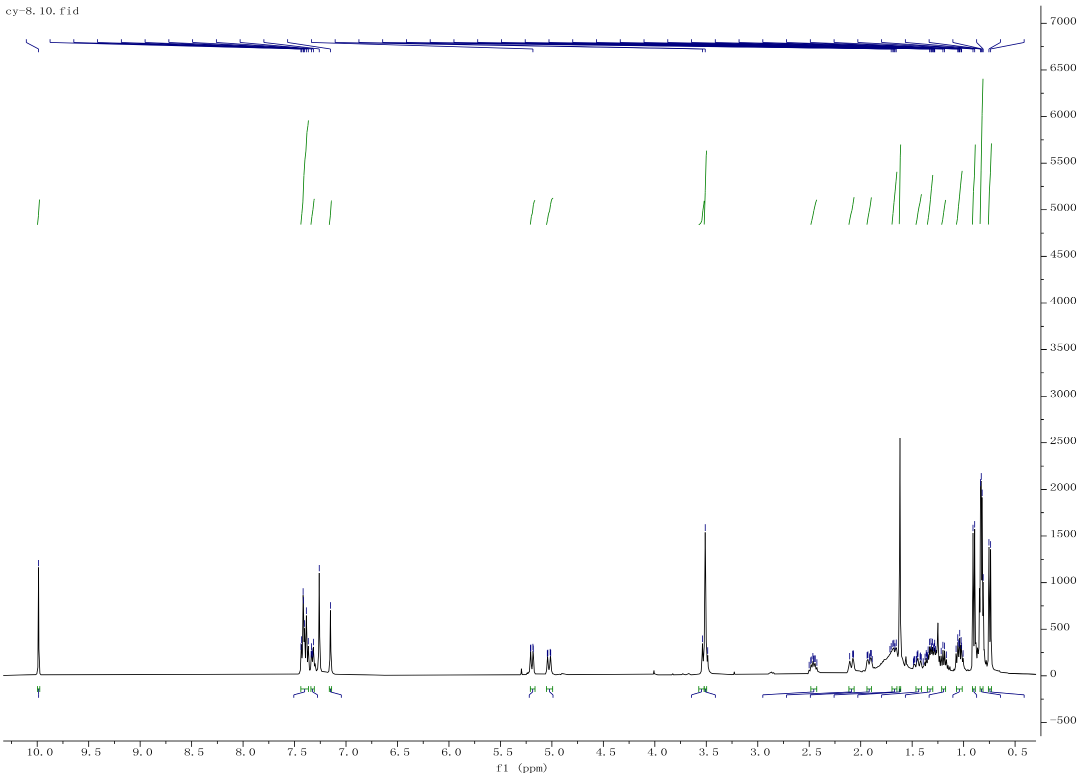

Figure S2 ^{1}H NMR of **1**

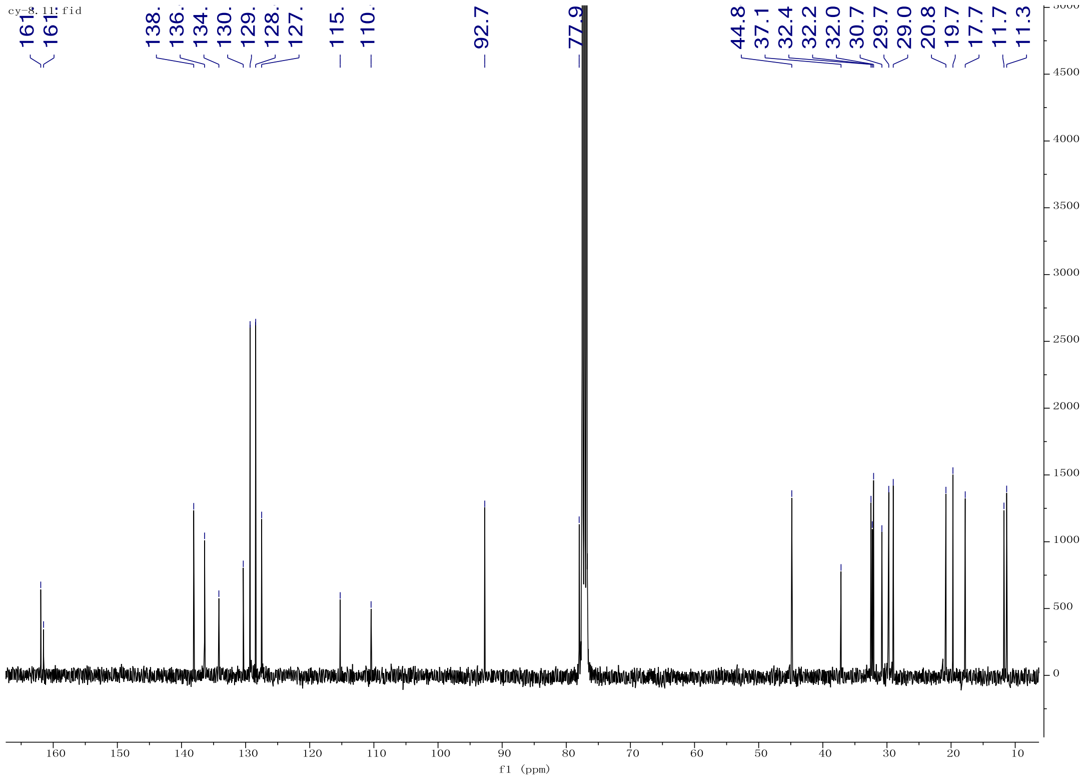

Figure S3 ^{13}C NMR of **1**

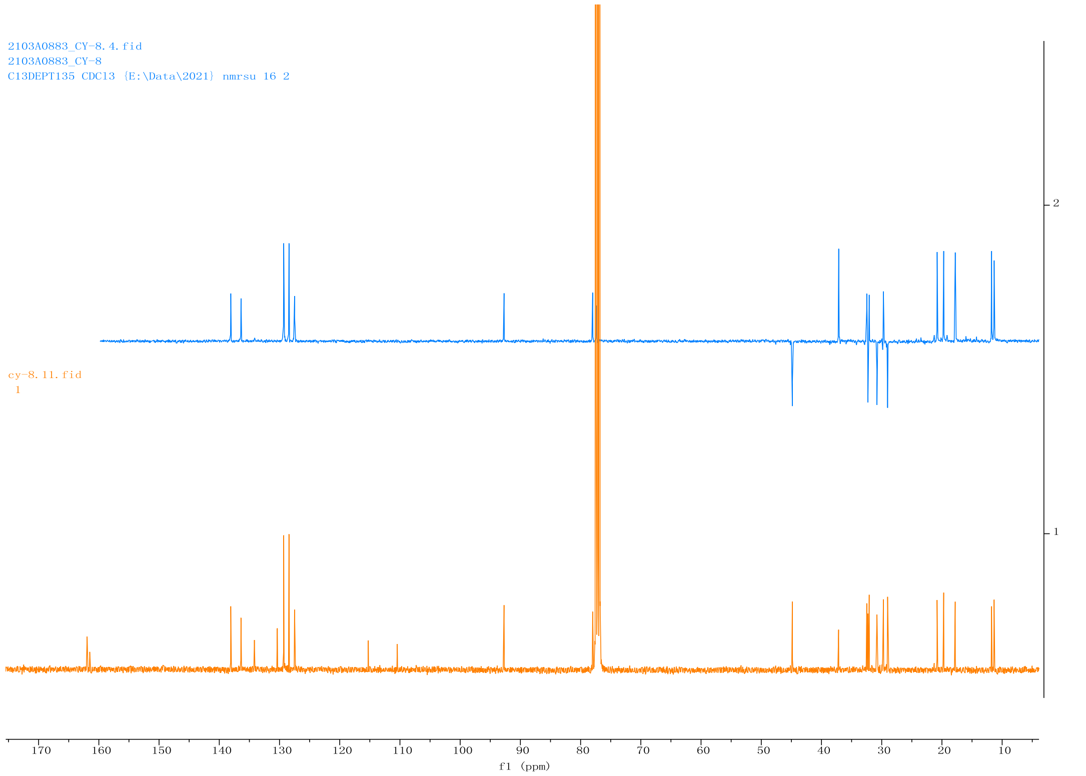

Figure S4 DEPT 135 of **1**

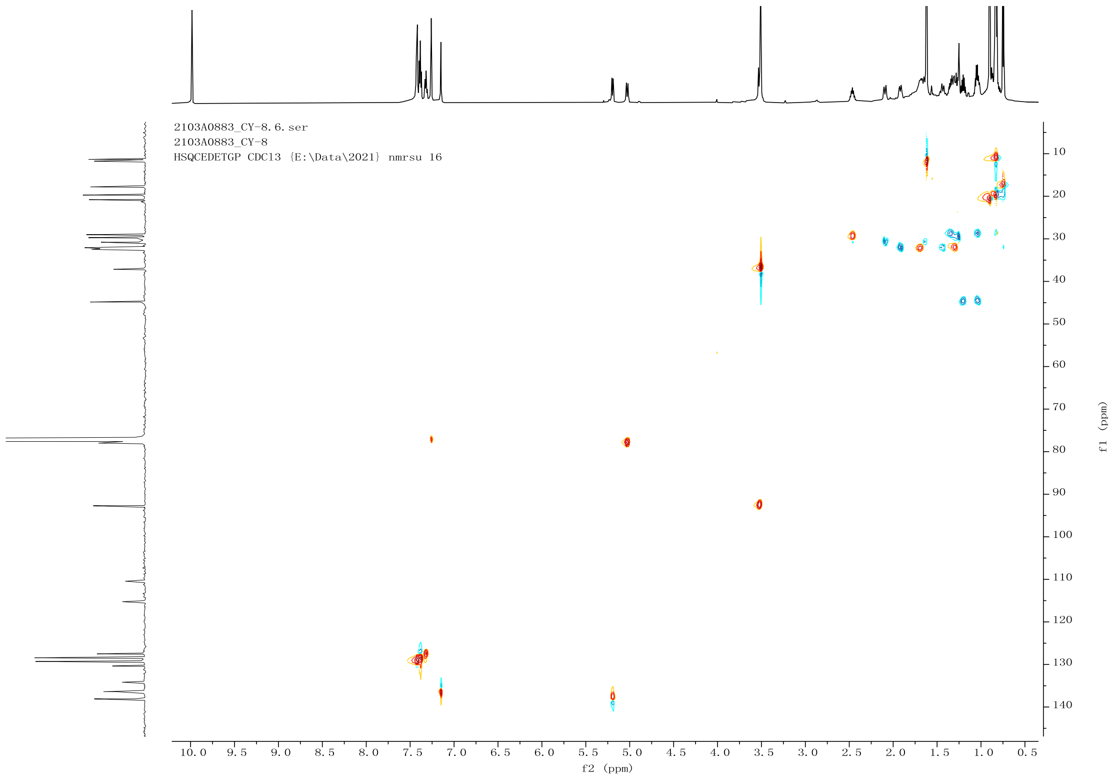

Figure S5 HSQC spectrum of **1**

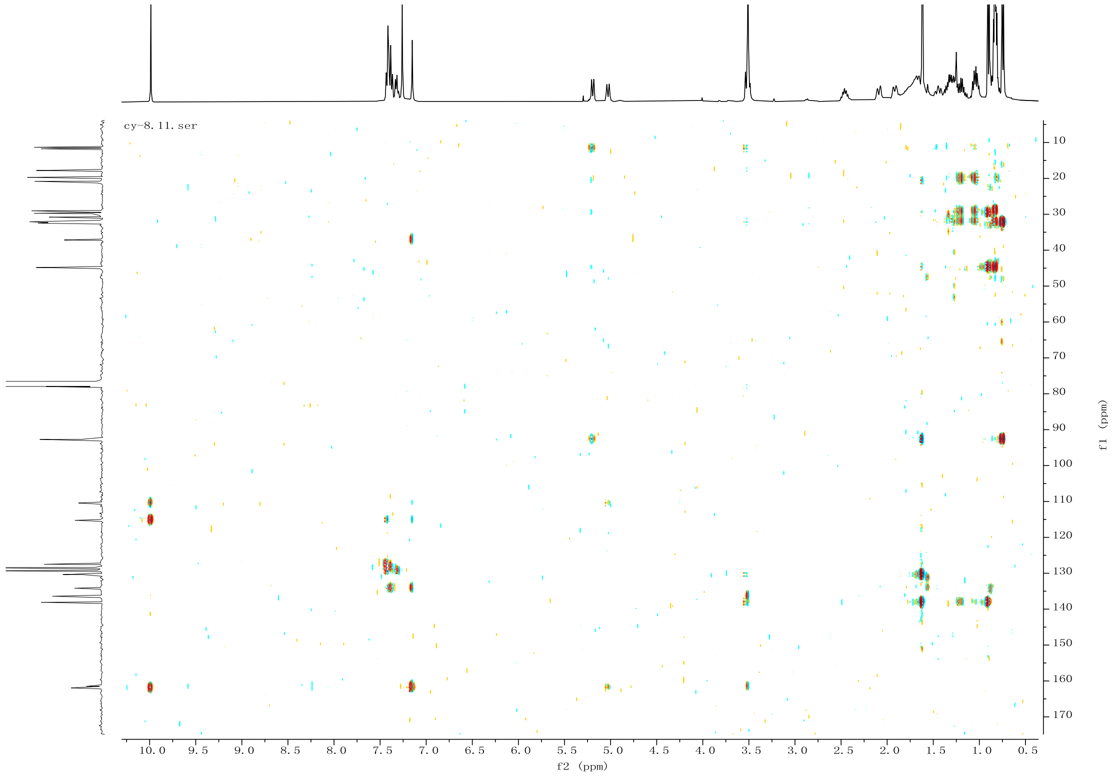

Figure S6 HMBC spectrum of **1**

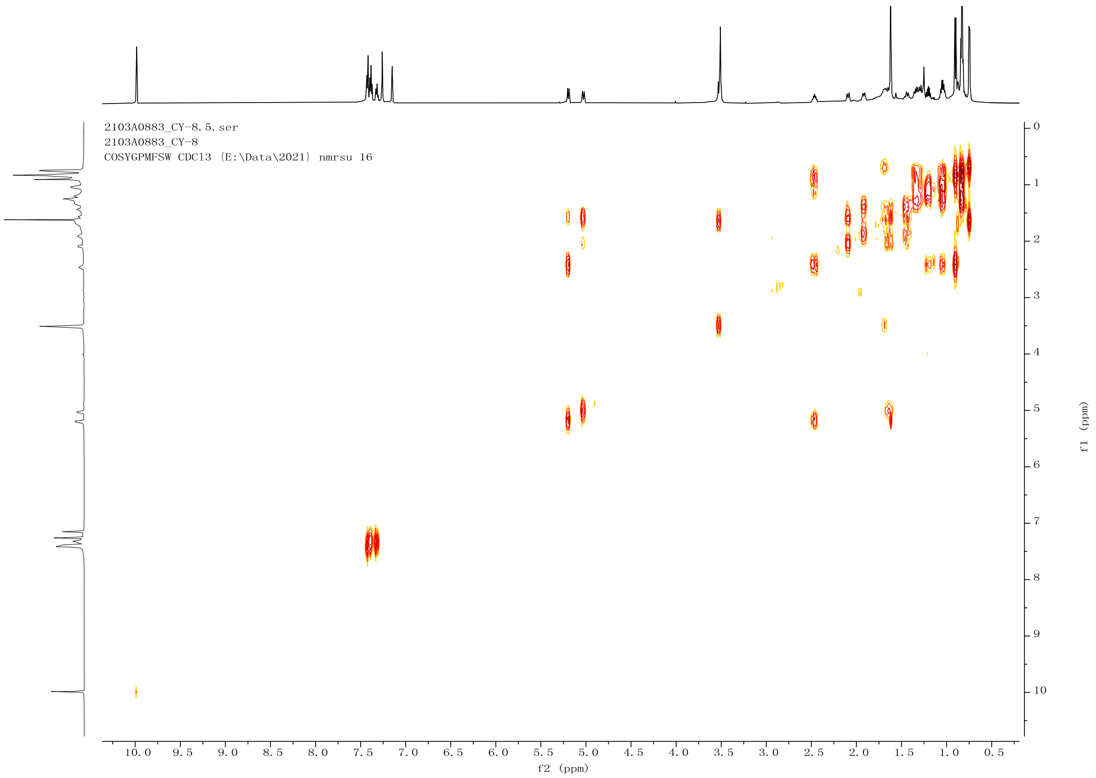

Figure S7 COSY spectrum of **1**

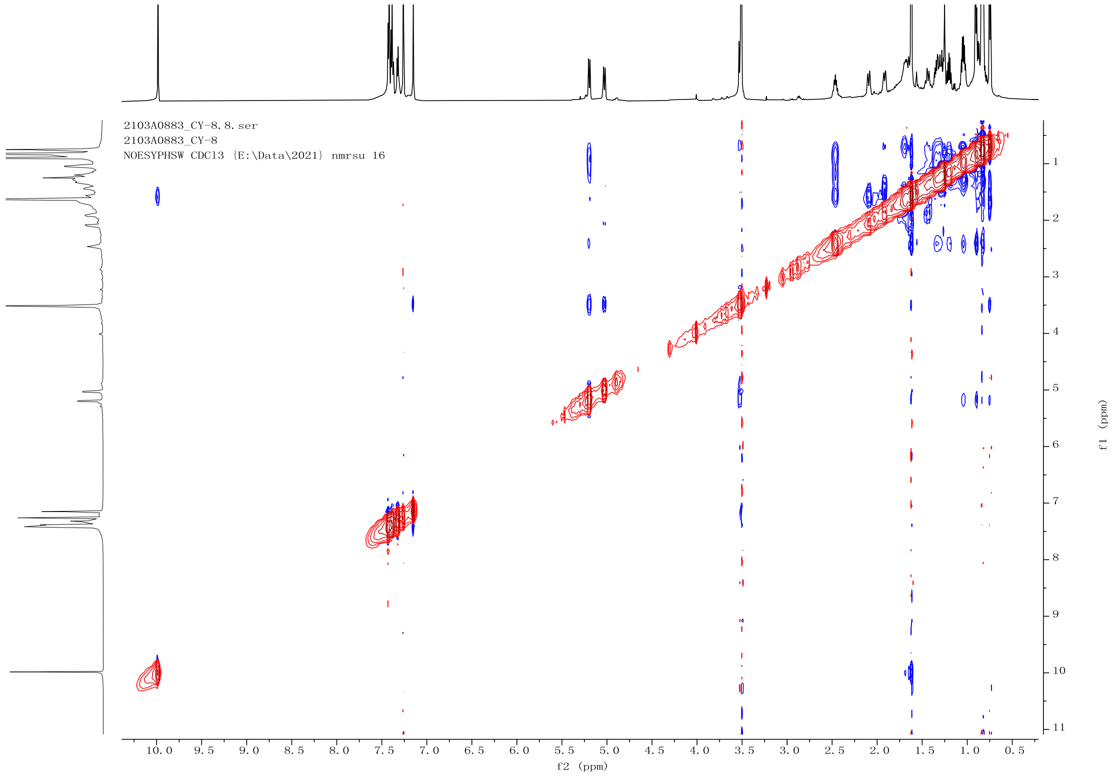

Figure S8 NOESY spectrum of **1**

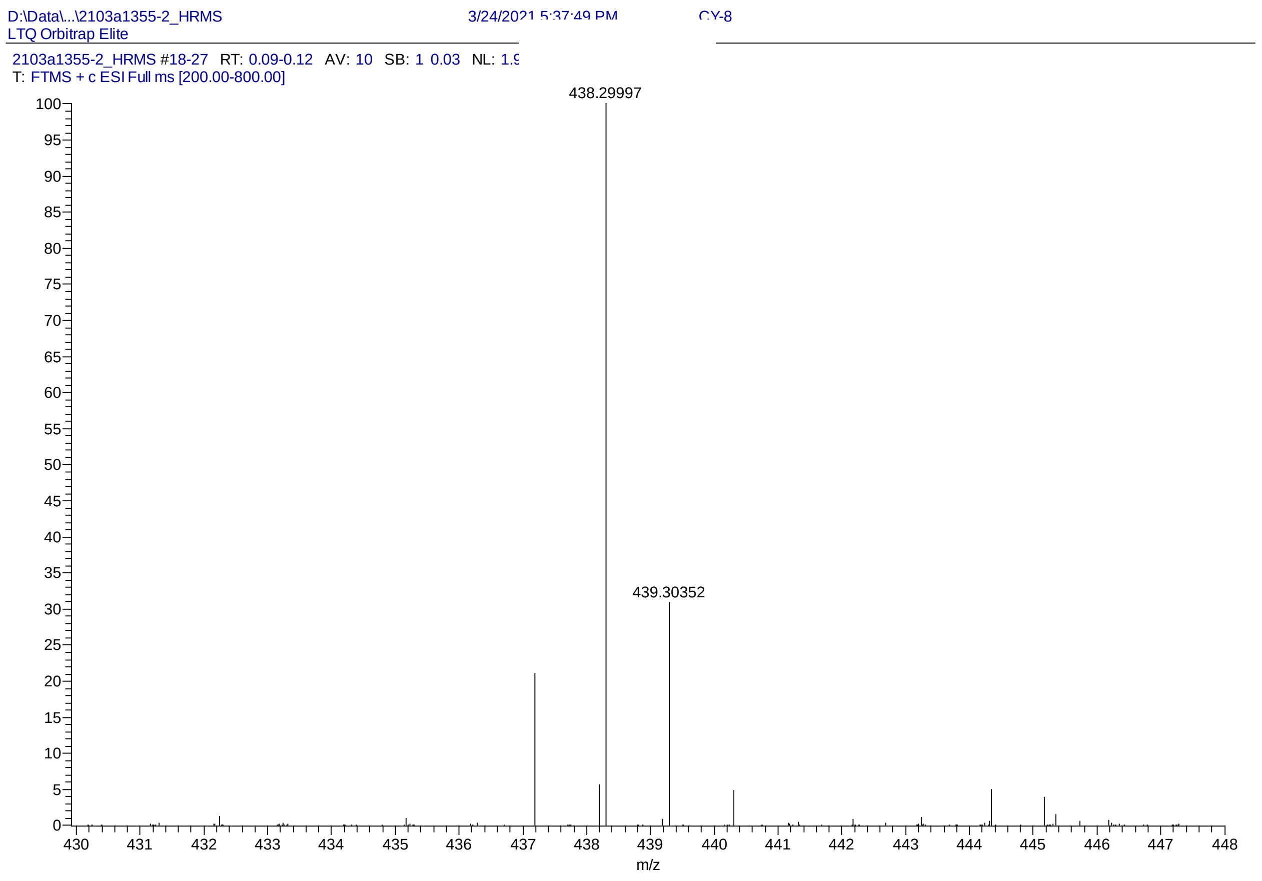

Figure S9 HR-ESI-MS spectrum of **1**

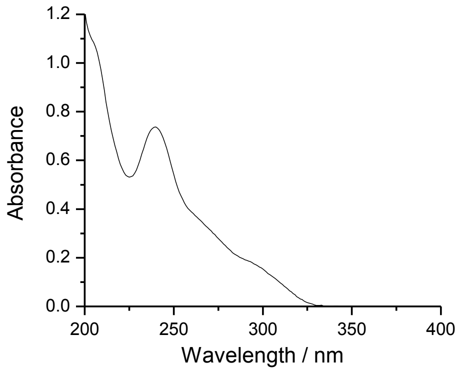

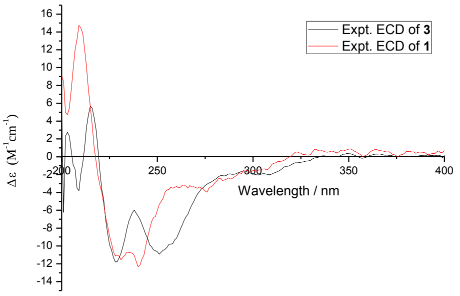

Figure S10 UV spectrum of **1** and ECD spectra of **1** and **3.**

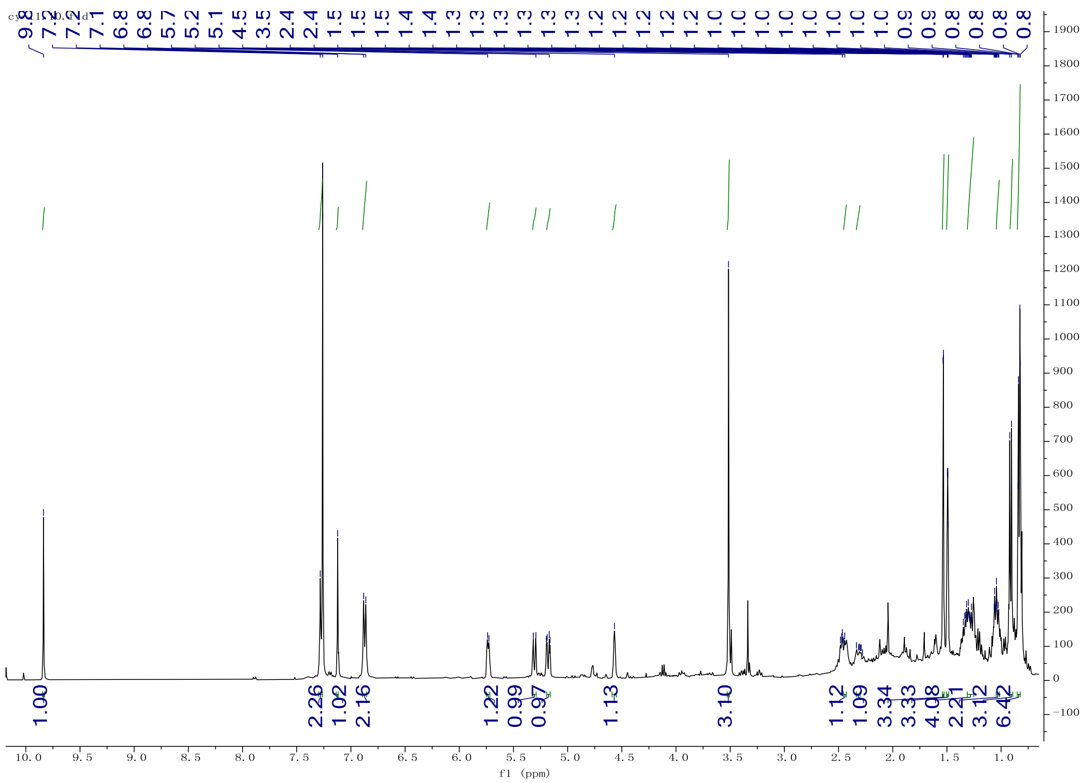

Figure S11 ^{1}H NMR of **2**

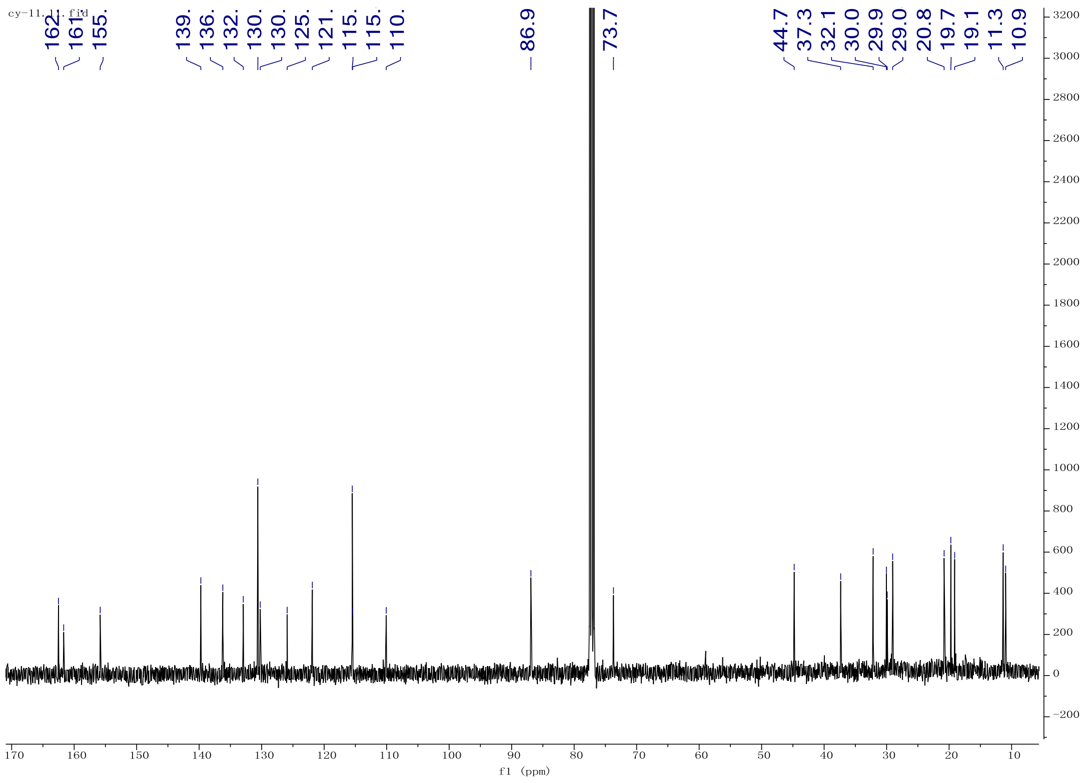

Figure S12 ^{13}C NMR of **2**

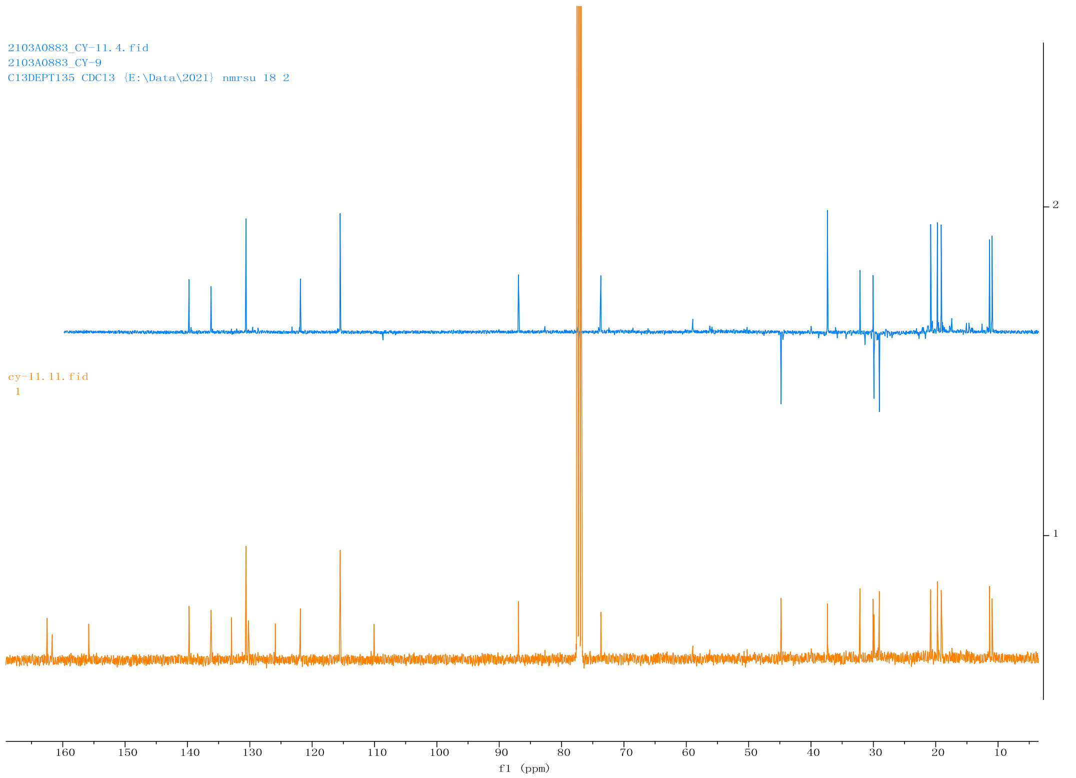

Figure S13 DEPT 135 of **2**

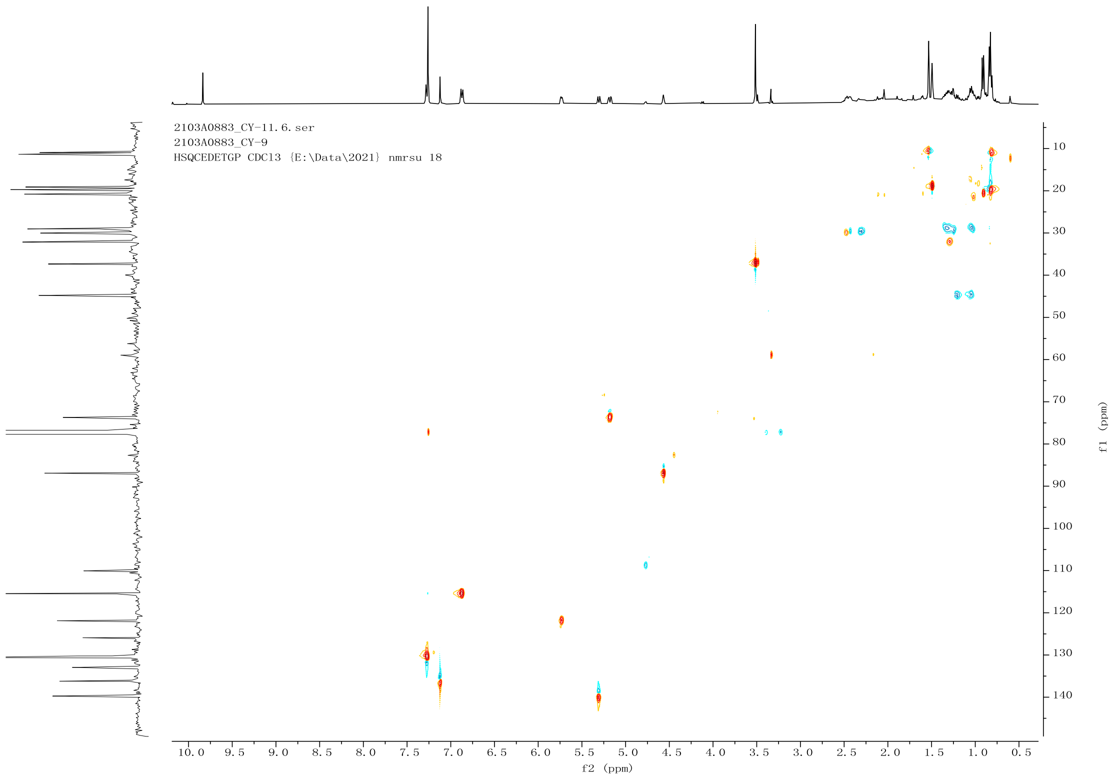

Figure S14 HSQC spectrum of **2**

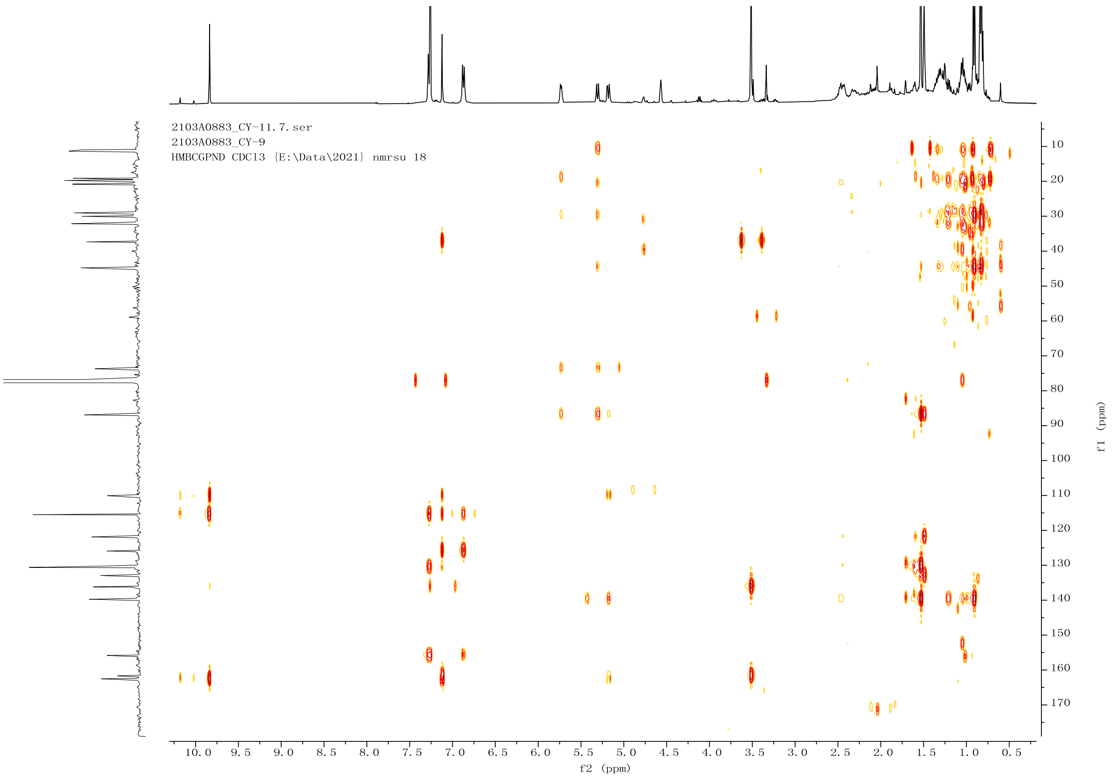

Figure S15 HMBC spectrum of **2**

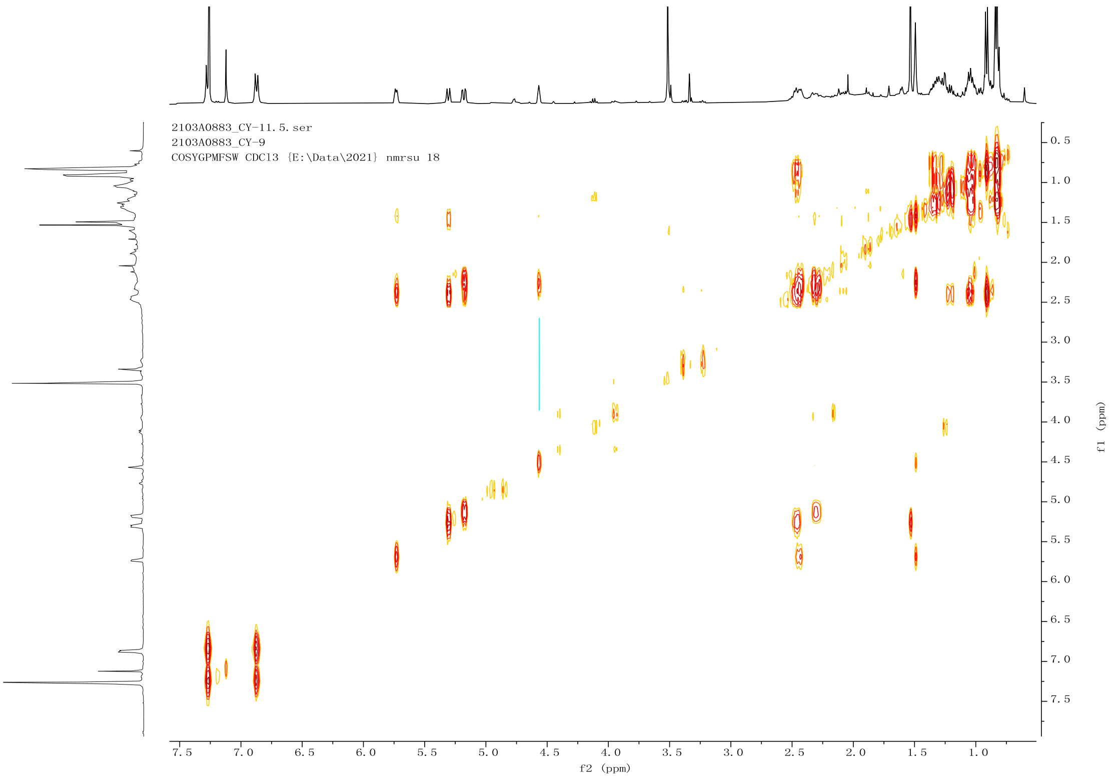

Figure S16 COSY spectrum of **2**

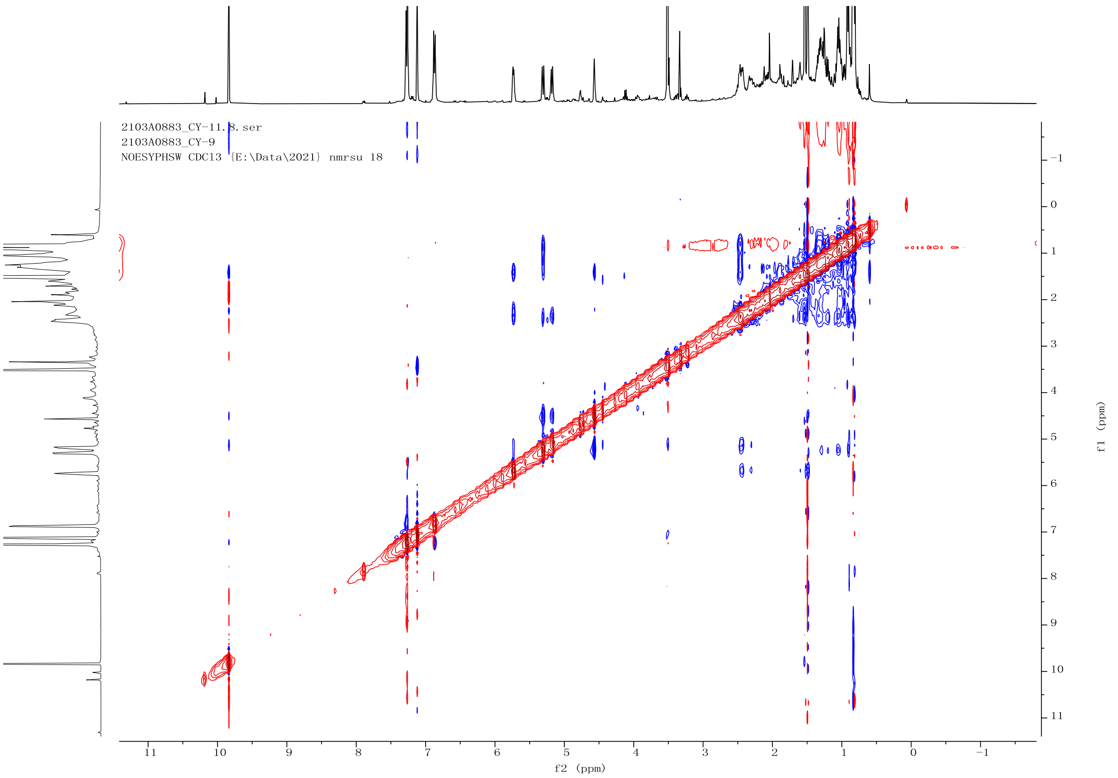

Figure S17 NOESY spectrum of **2**

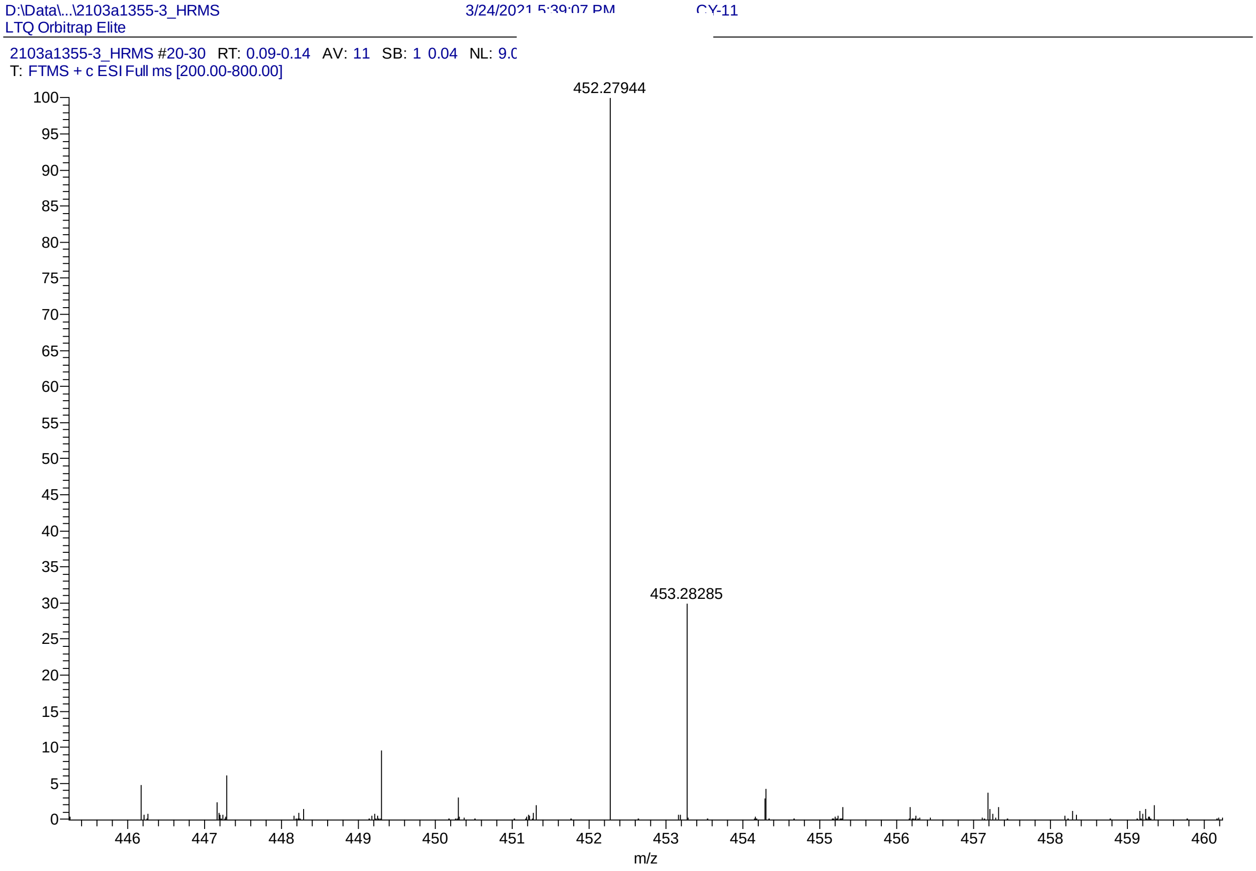

Figure S18 HR-ESI-MS spectrum of **2**

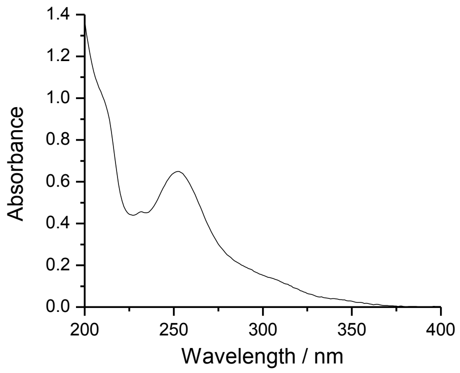

Figure S19 UV spectrum of **2**

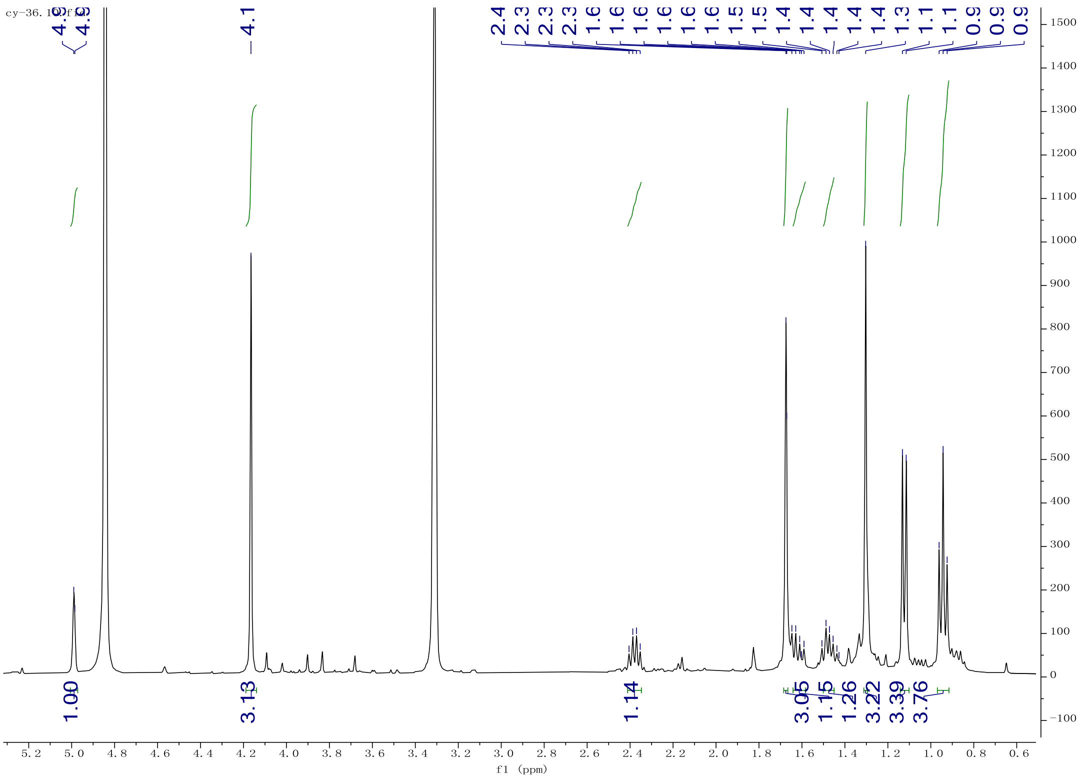

Figure S20 ^{1}H NMR of **7**

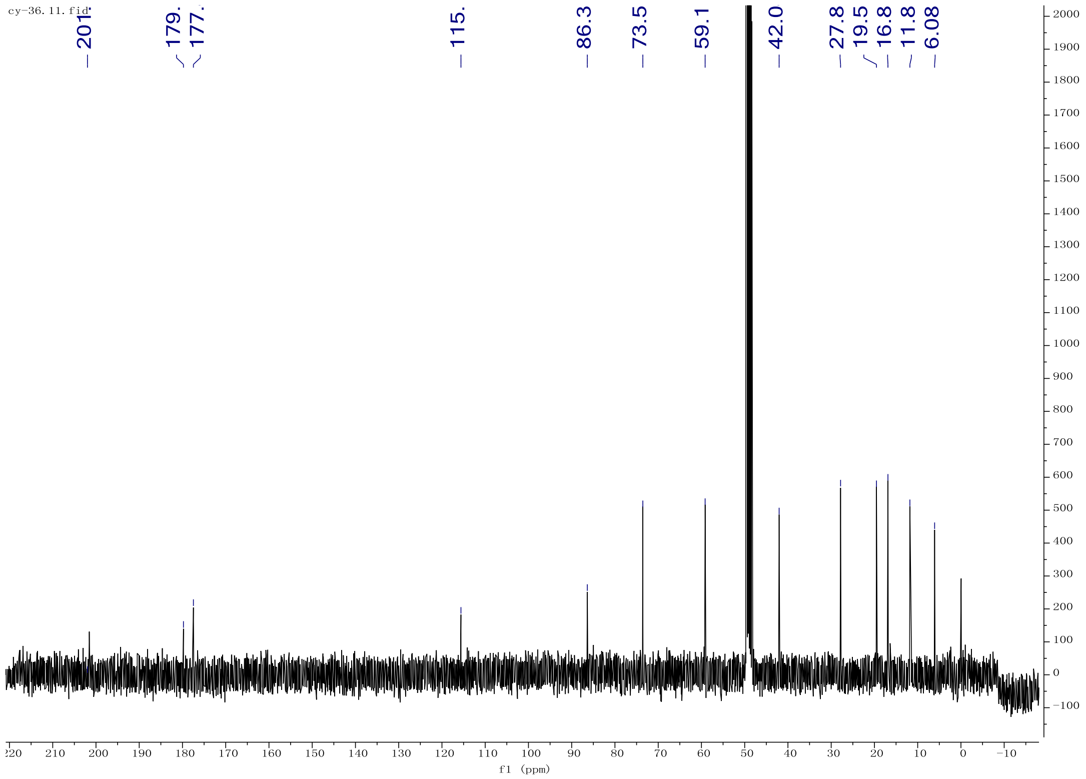

Figure S21 ^{13}C NMR of **7**

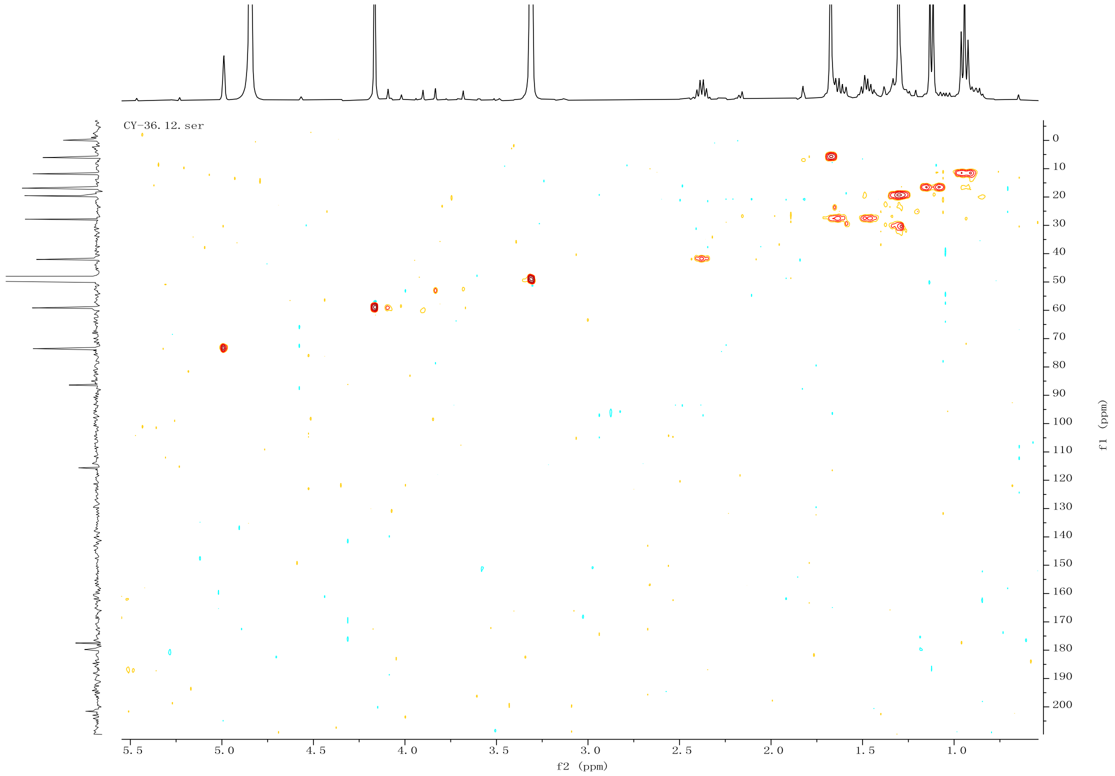

Figure S22 HSQC spectrum of **7**

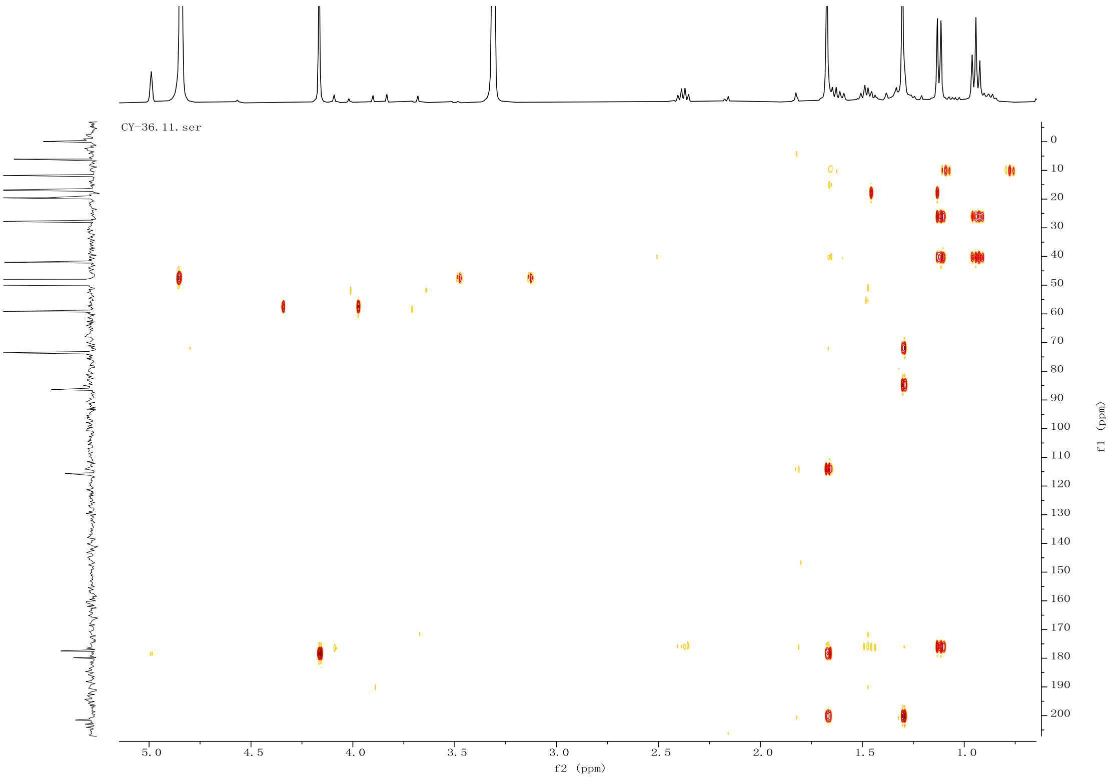

Figure S23 HMBC spectrum of **7**

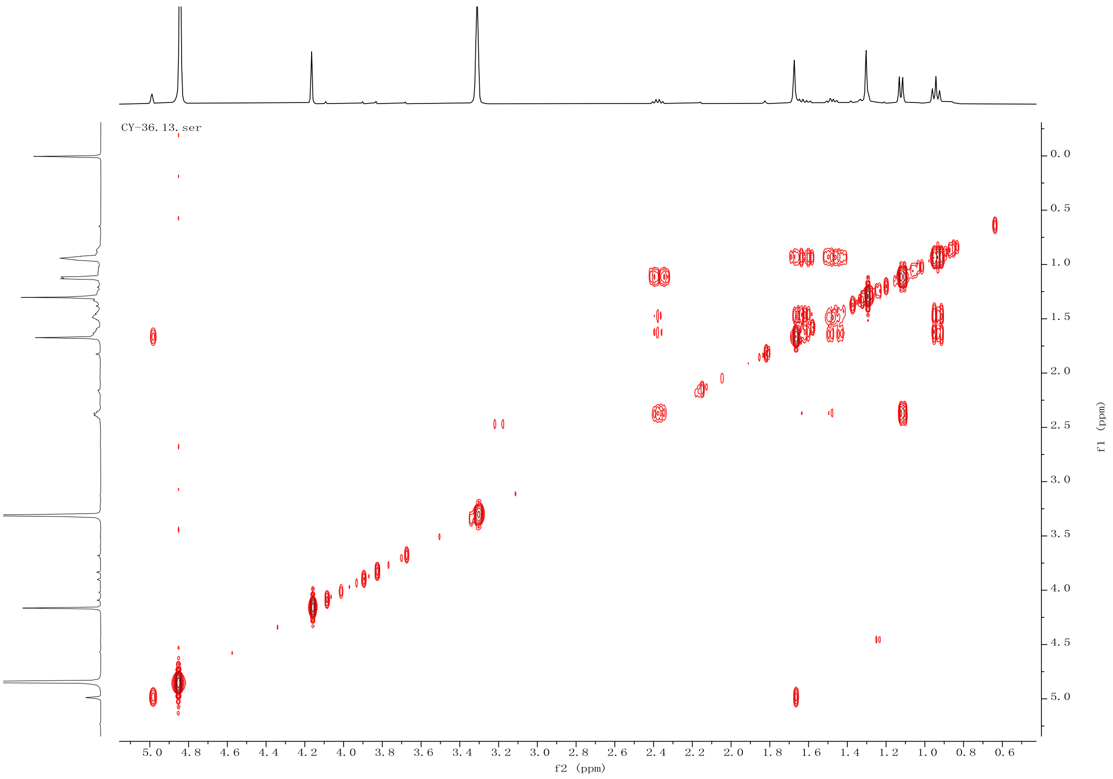

Figure S24 COSY spectrum of **7**

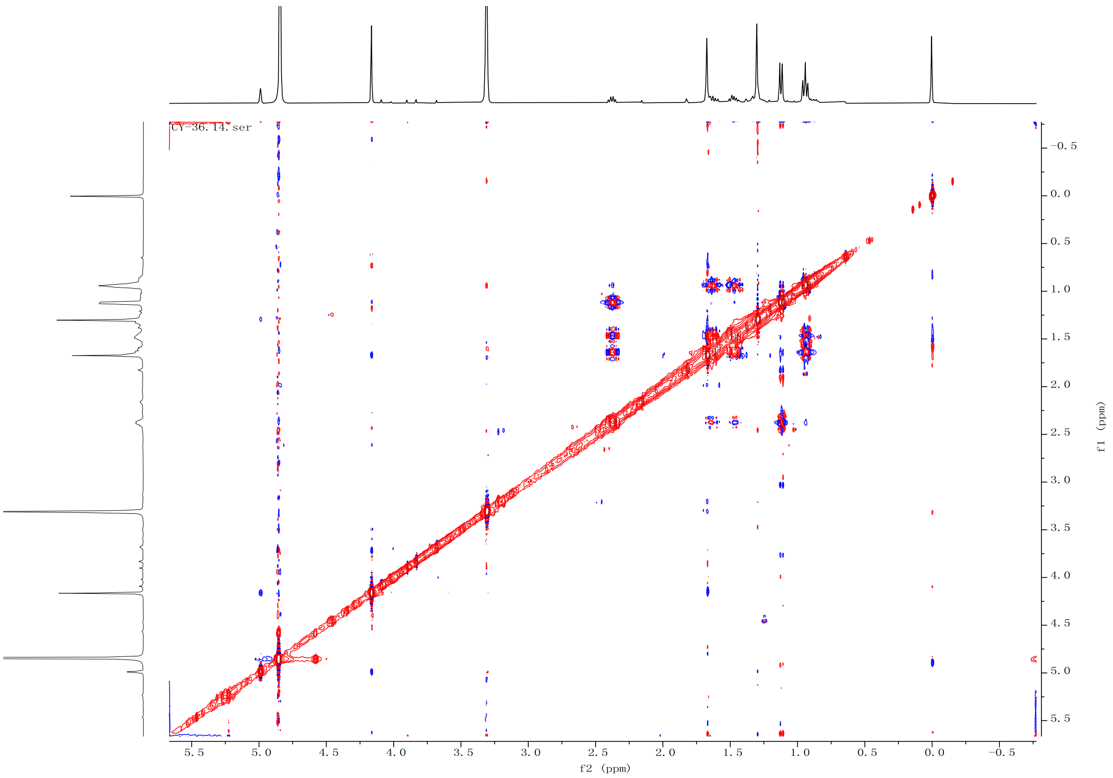

Figure S25 NOESY spectrum of **7**

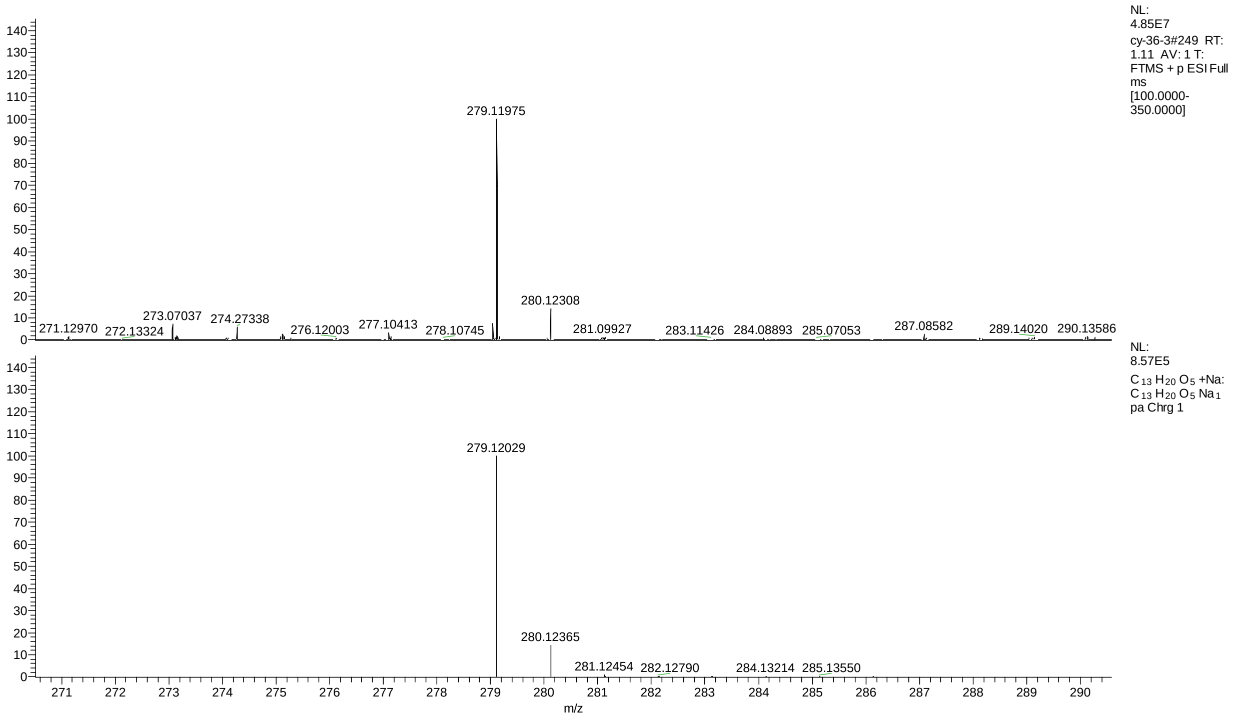

Figure S26 HR-ESI-MS spectrum of **7**

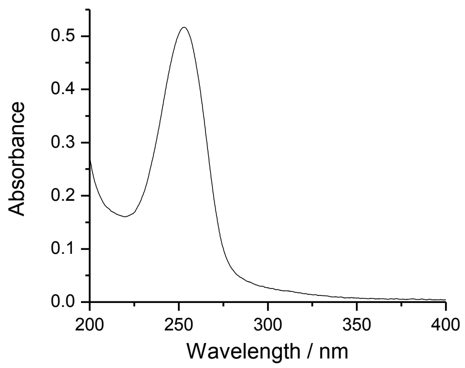

Figure S27 UV spectrum of **7**

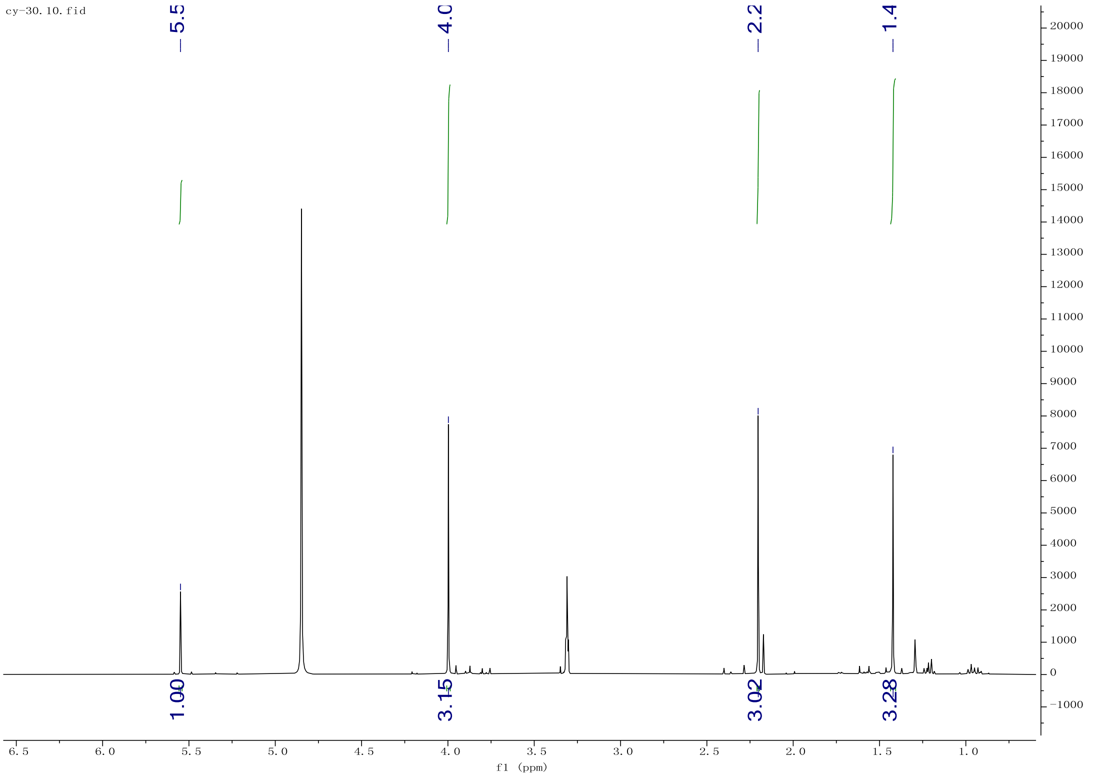

Figure S28 ^{1}H NMR of **8**

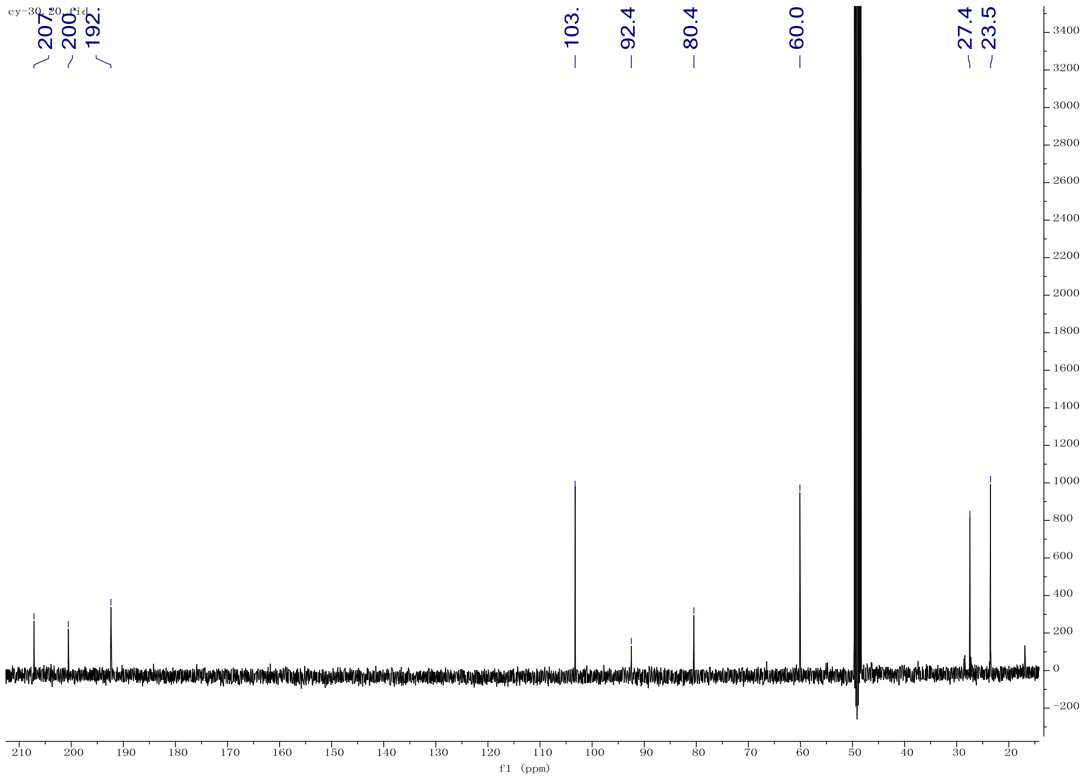

Figure S29 ^{13}C NMR of **8**

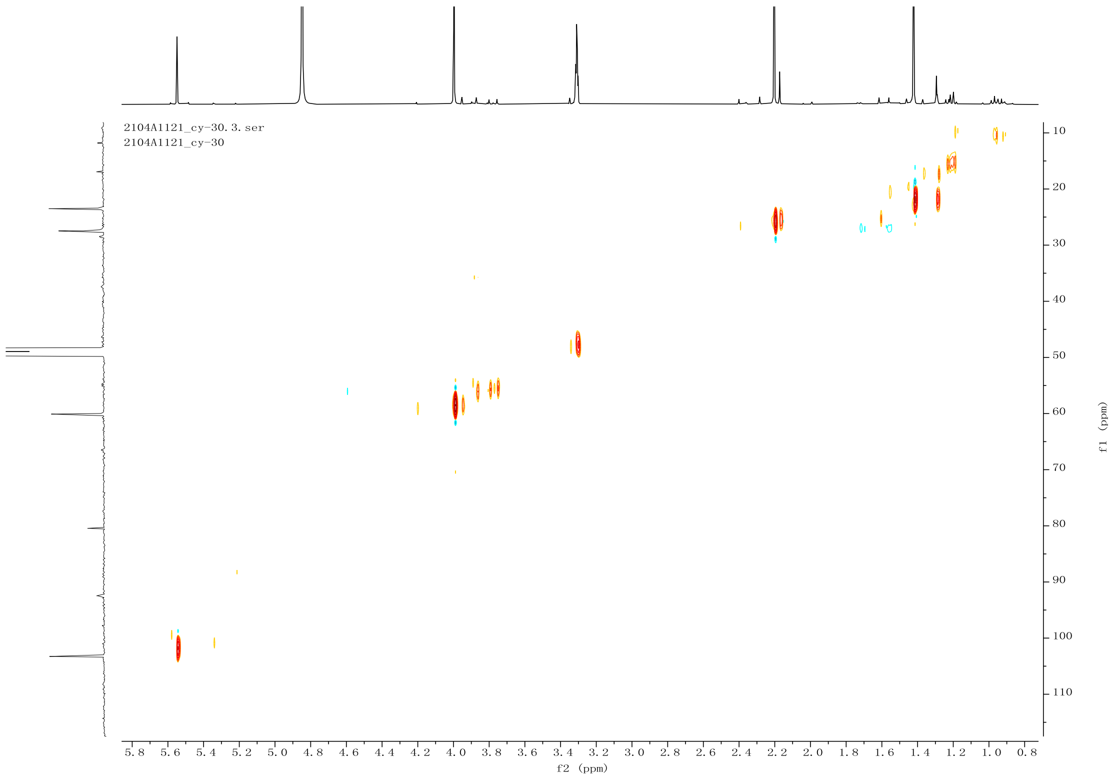

Figure S30 HSQC spectrum of **8**

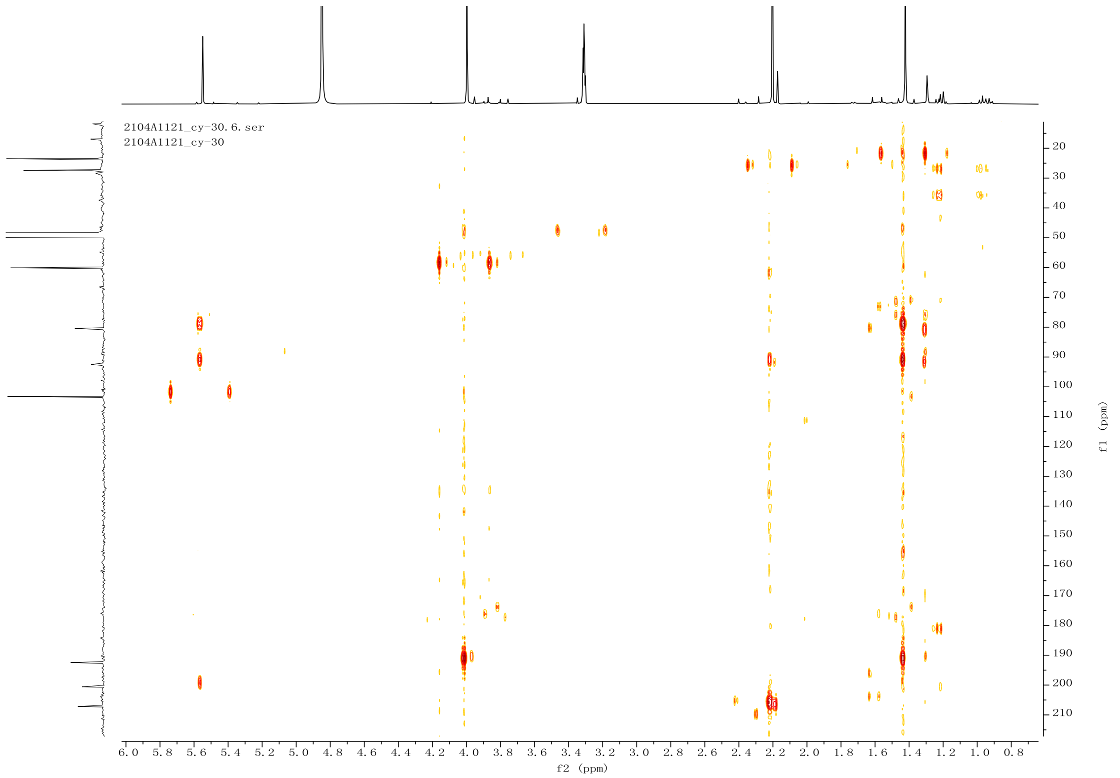

Figure S31 HMBC spectrum of **8**

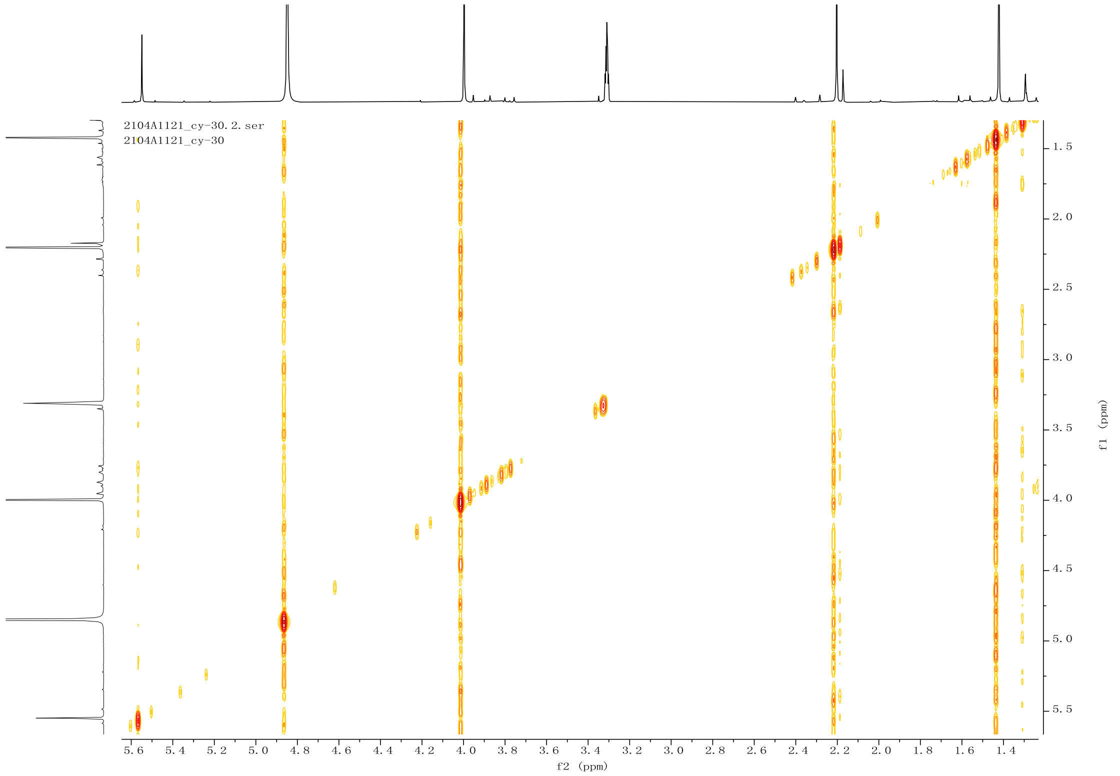

Figure S32 COSY spectrum of **8**

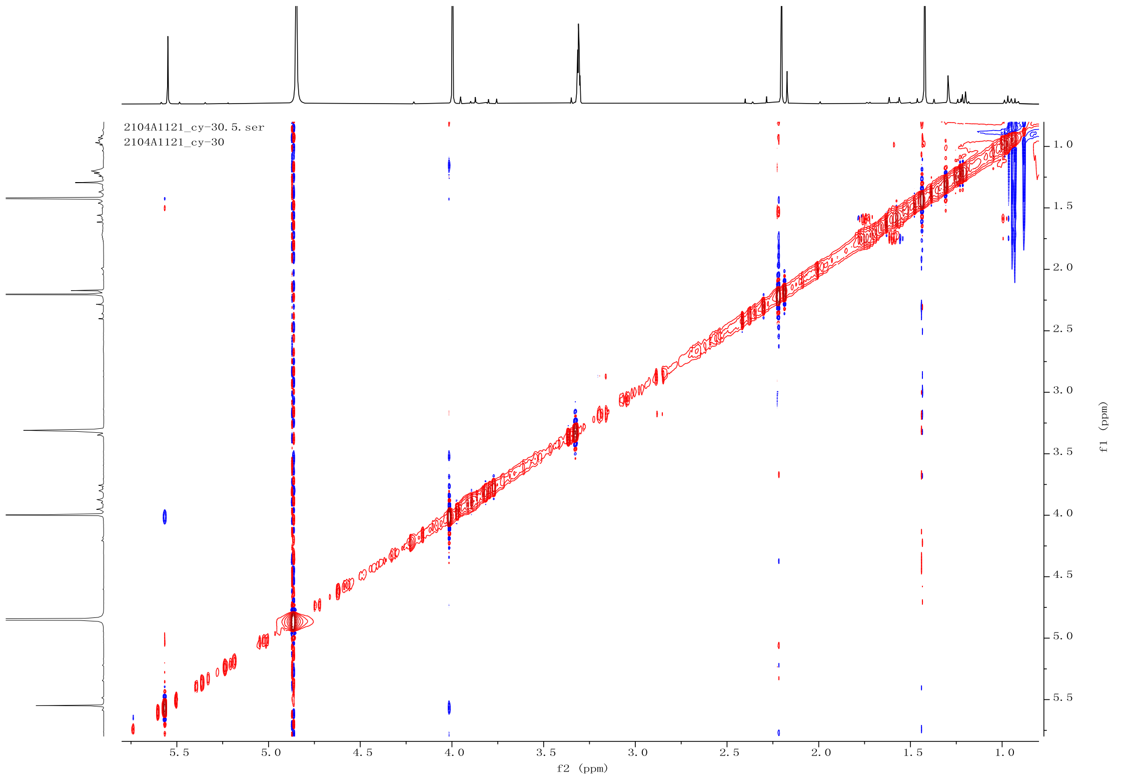

Figure S33 NOESY spectrum of **8**

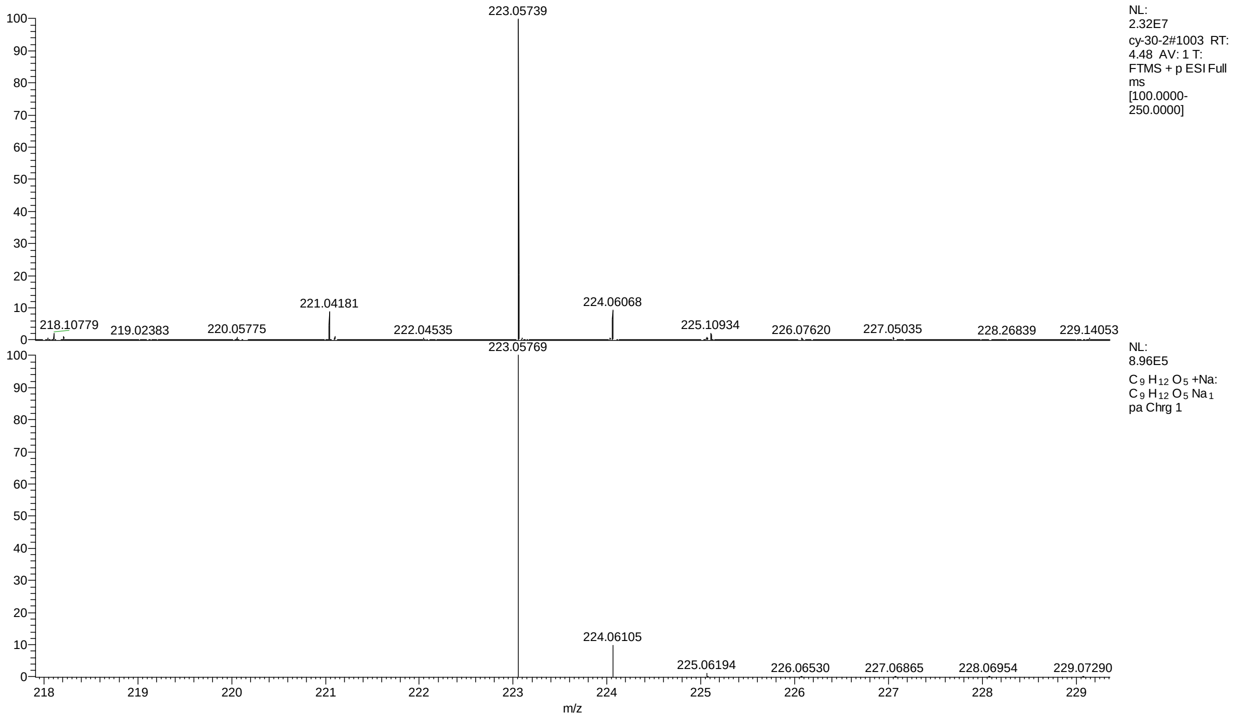

Figure S34 HR-ESI-MS spectrum of **8**

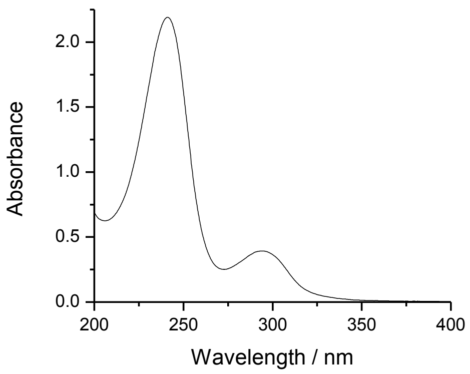

Figure S35 UV spectrum of **8**

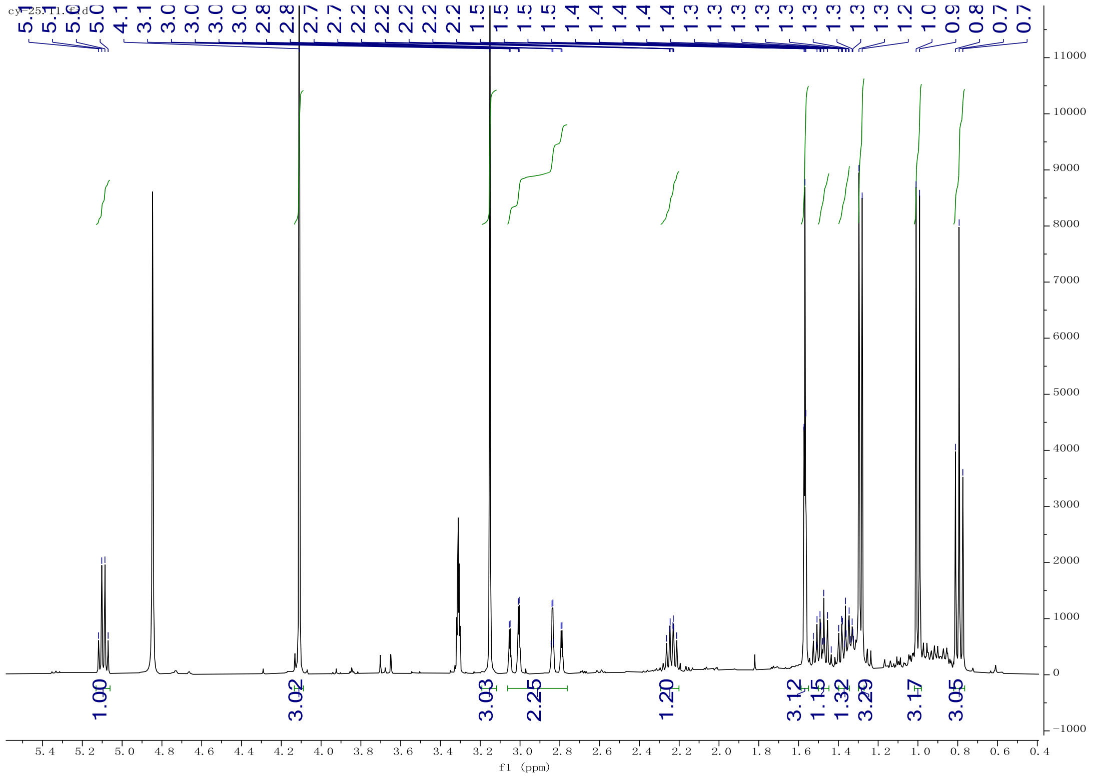

Figure S36 ^{1}H NMR of **9**

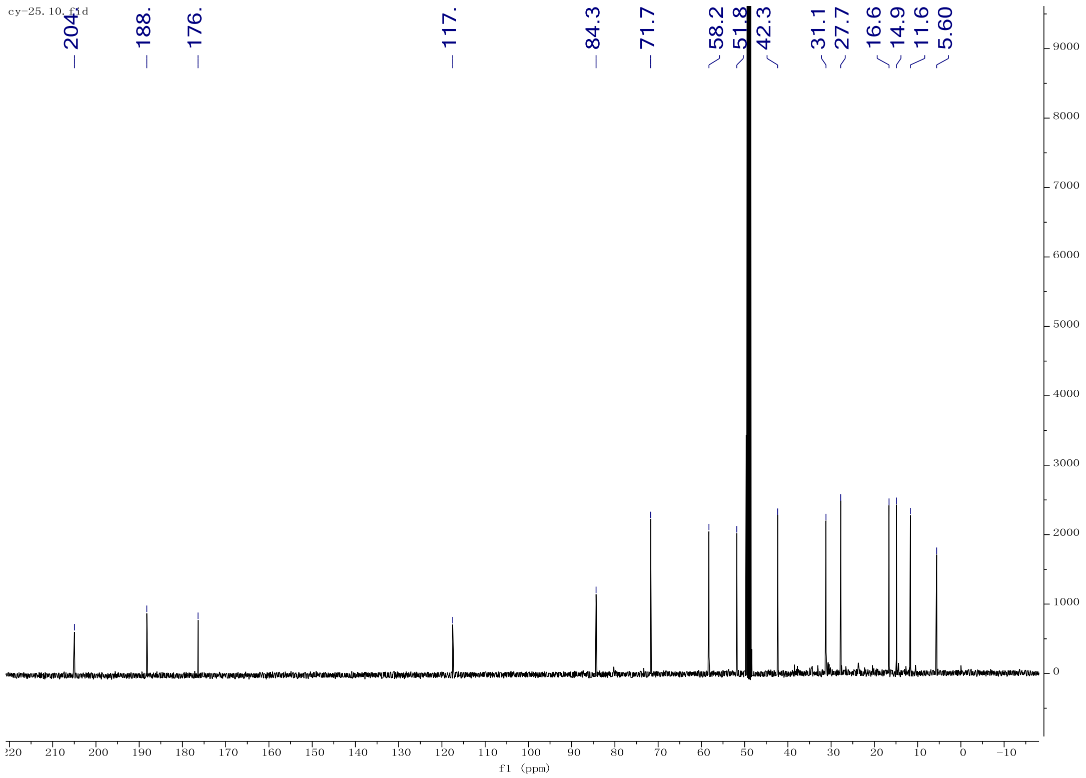

Figure S37 ^{13}C NMR of **9**

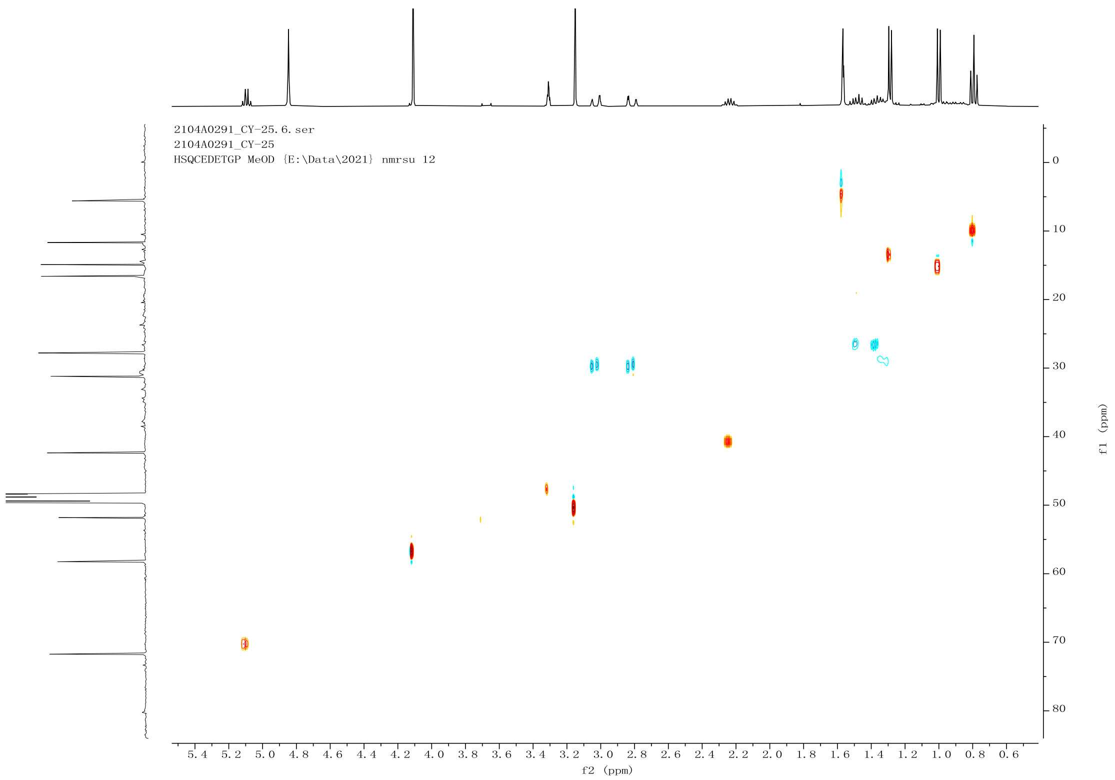

Figure S38 HSQC spectrum of **9**

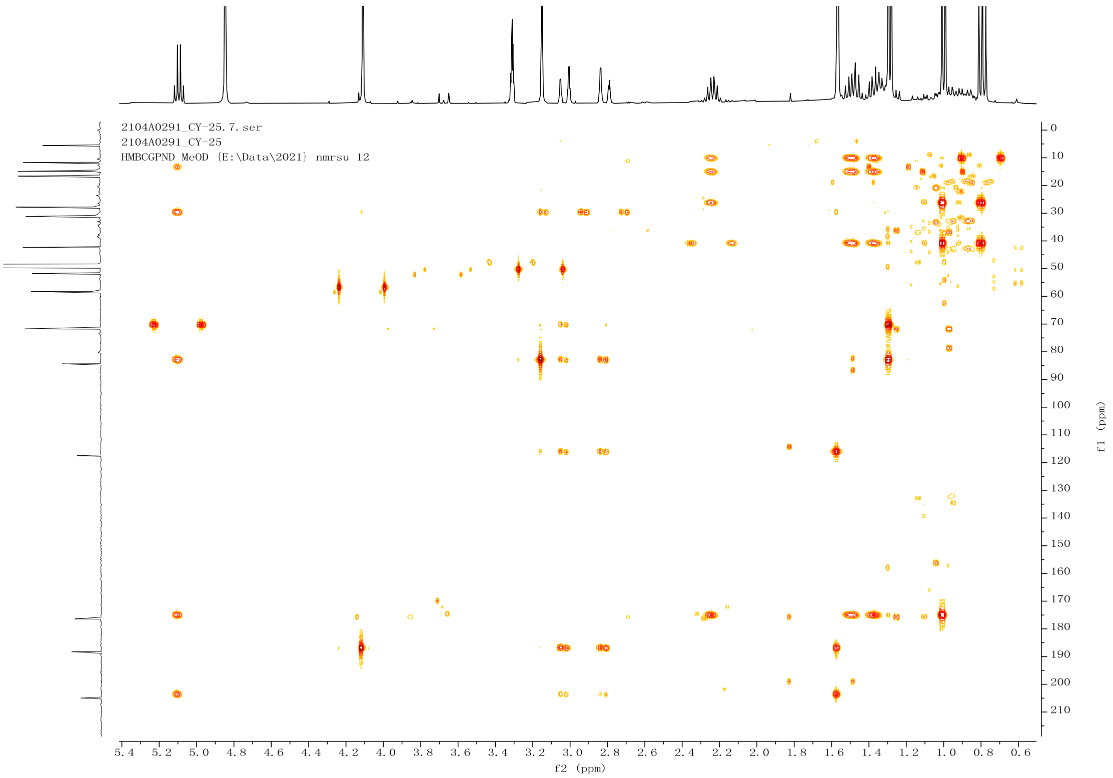

Figure S39 HMBC spectrum of **9**

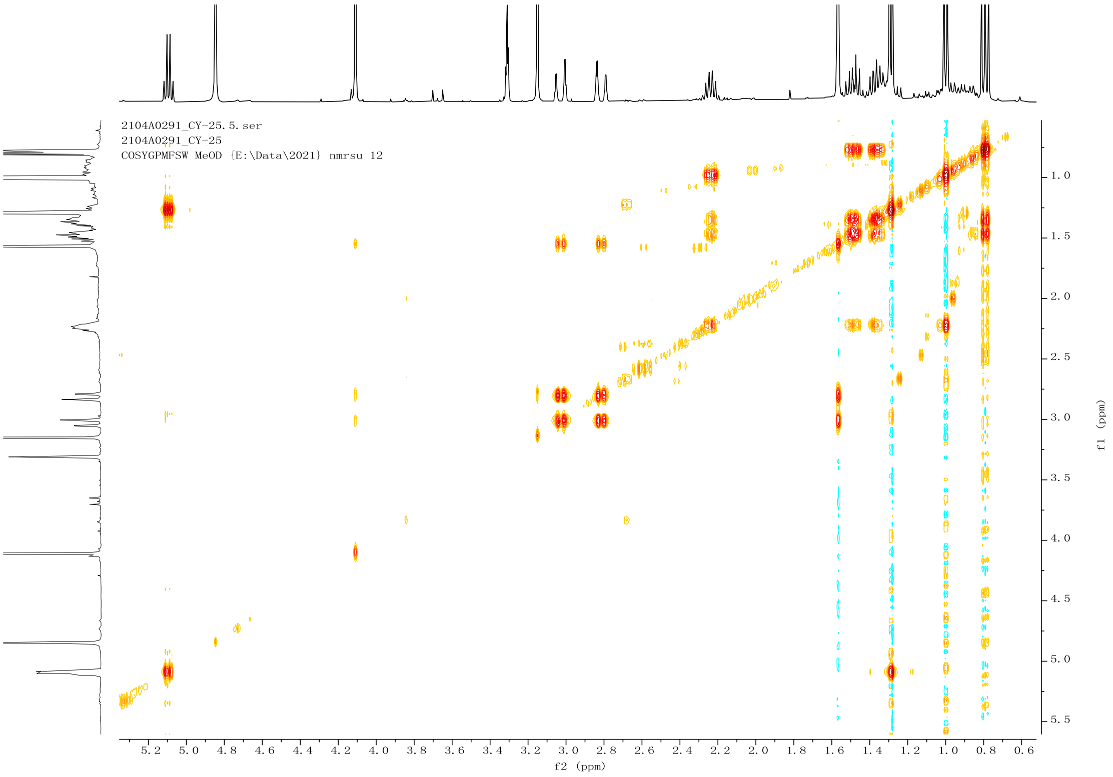

Figure S40 COSY spectrum of **9**

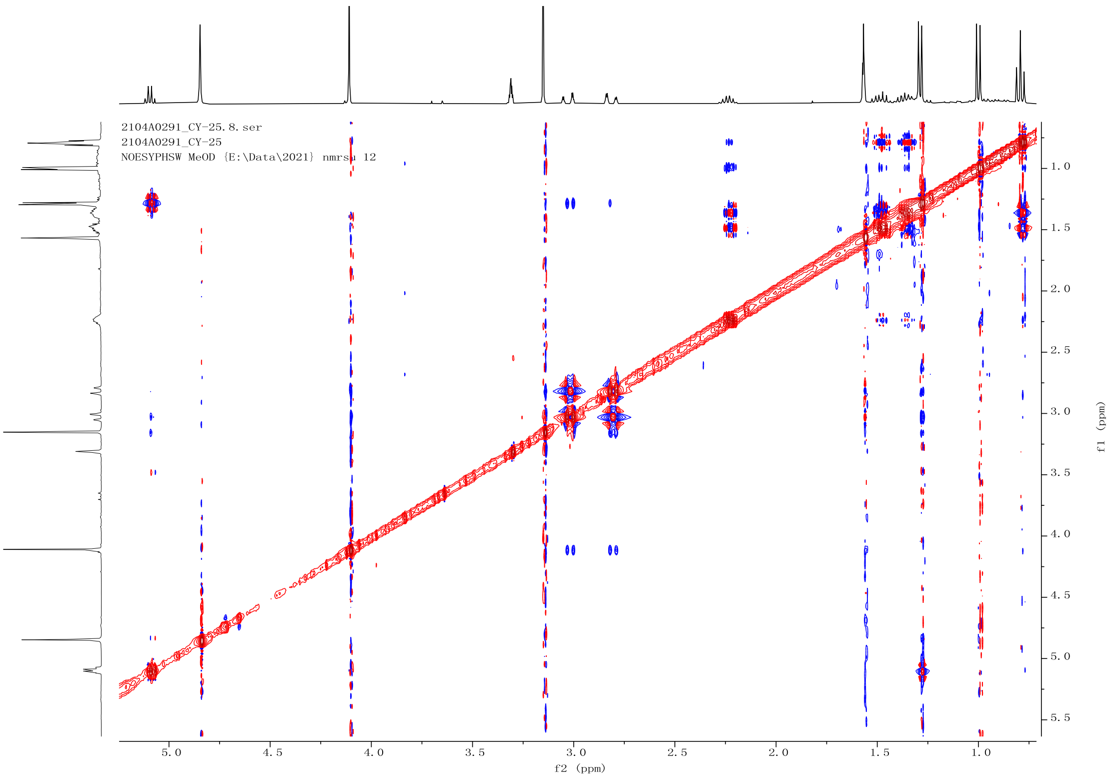

Figure S41 NOESY spectrum of **9**

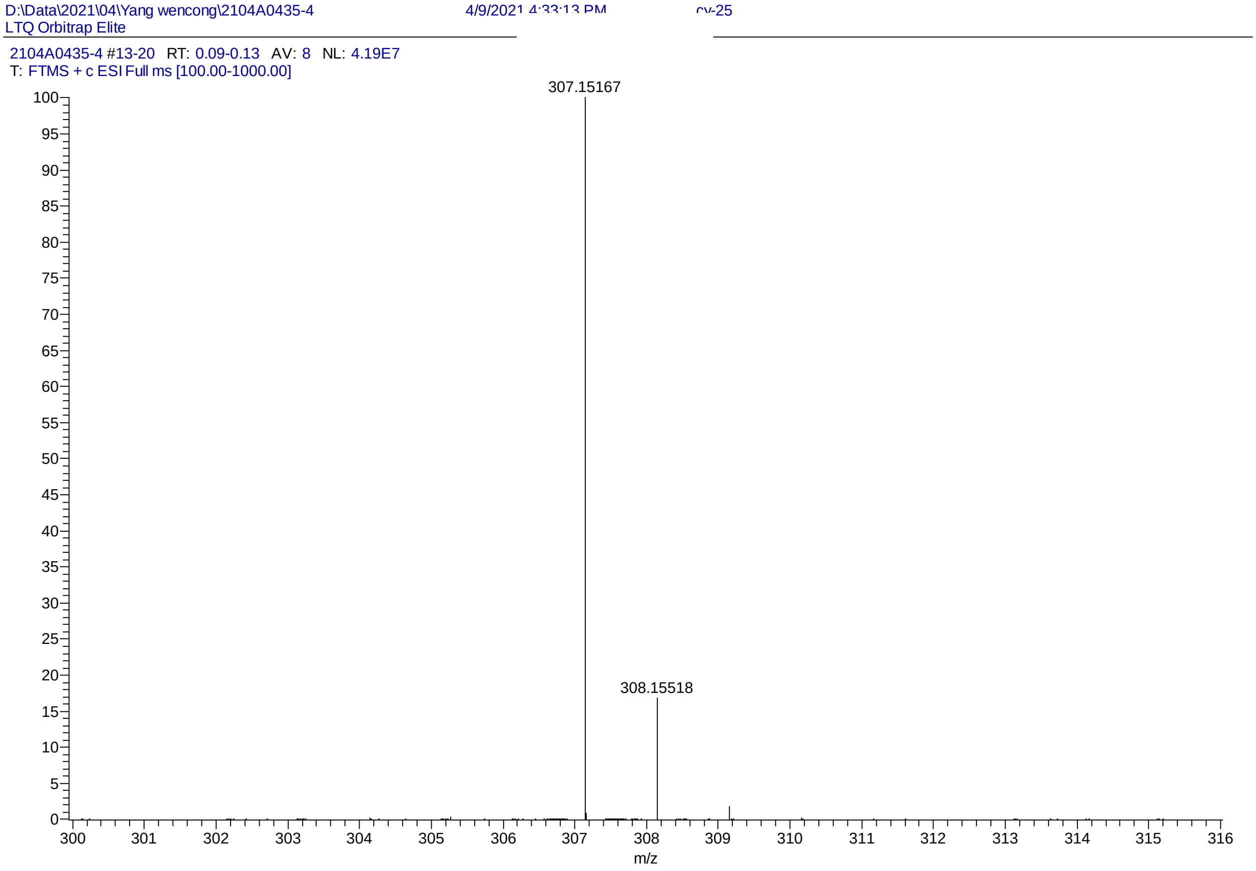

Figure S42 HR-ESI-MS spectrum of **9**

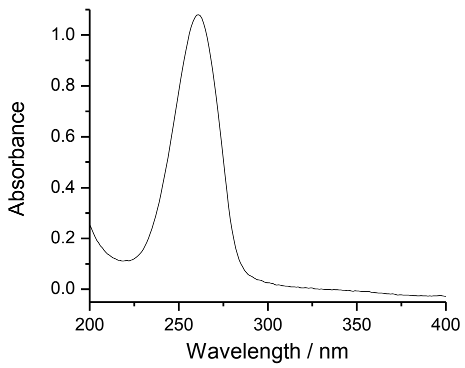

Figure S43 UV spectrum of **9**

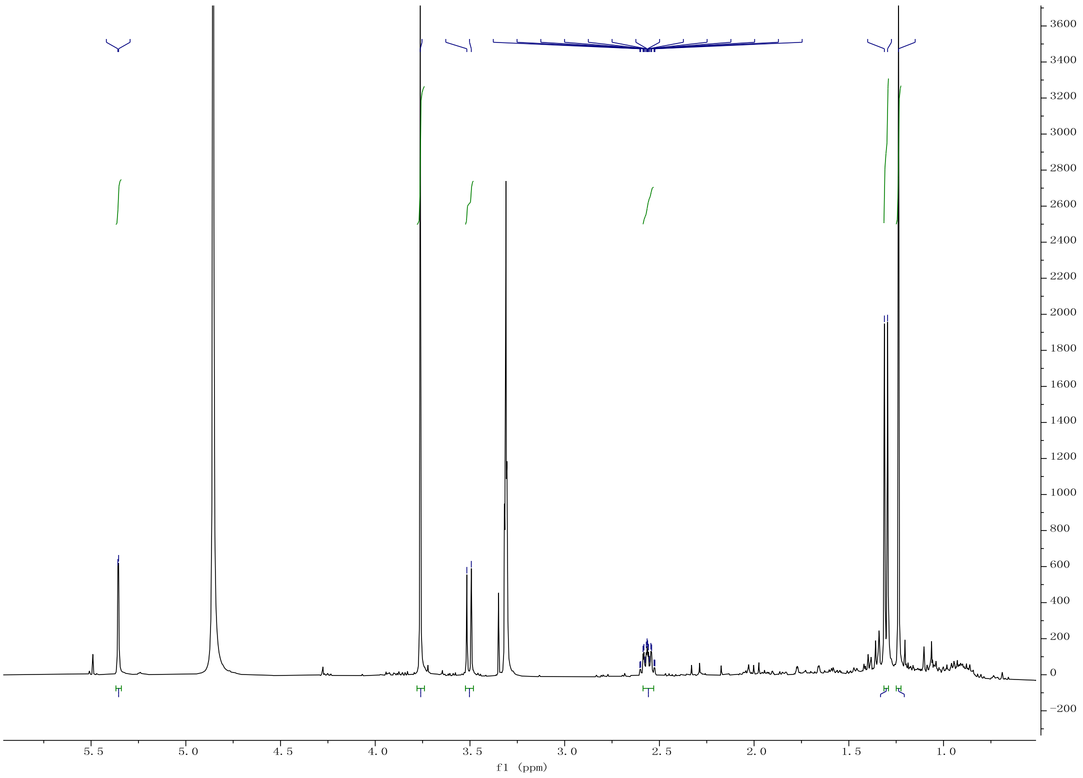

Figure S44 ^{1}H NMR of **10**

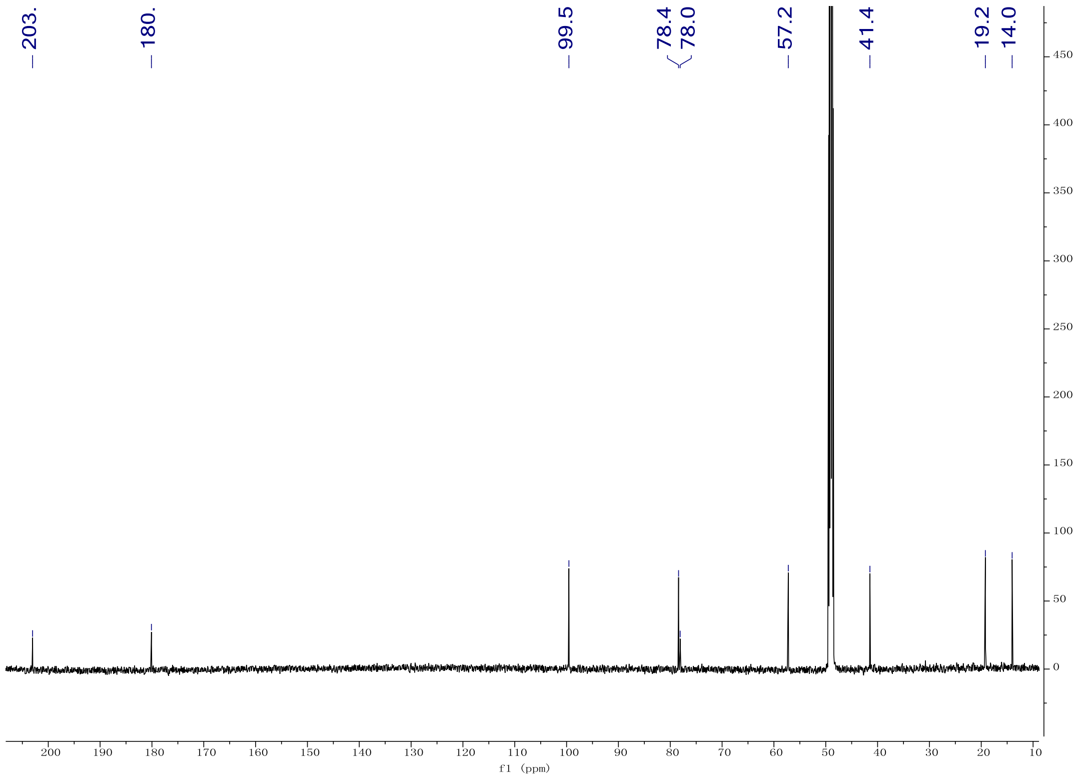

Figure S45 ^{13}C NMR of **10**

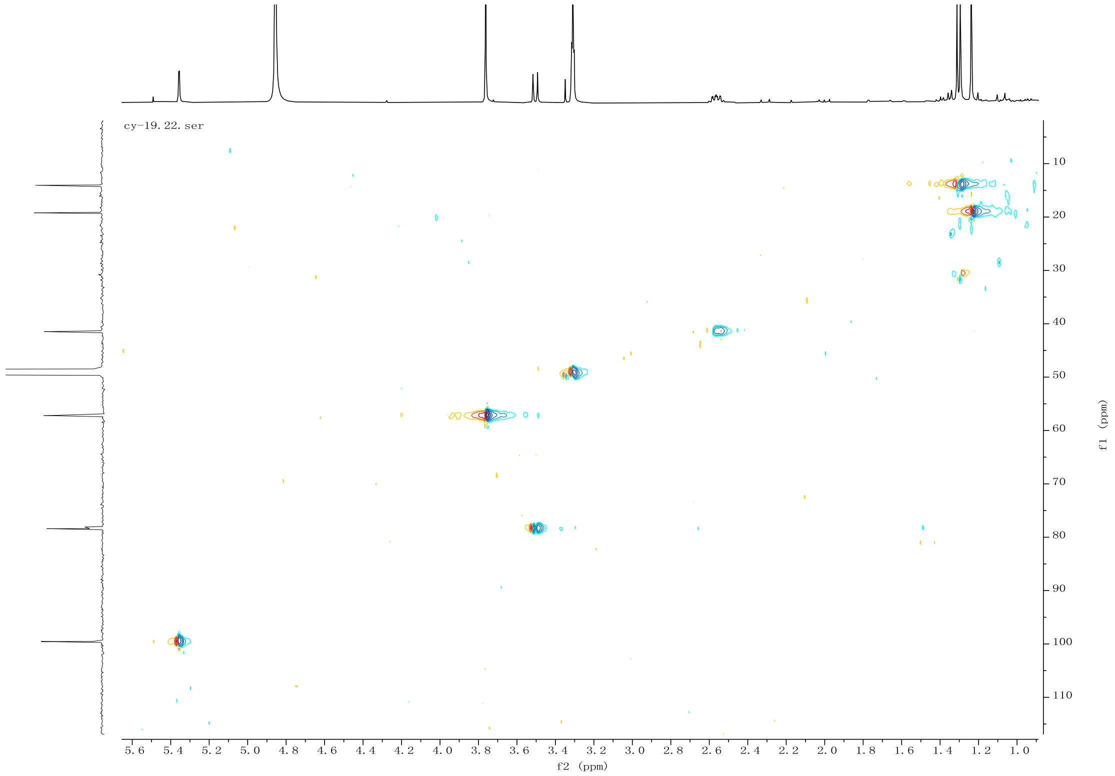

Figure S46 HSQC spectrum of **10**

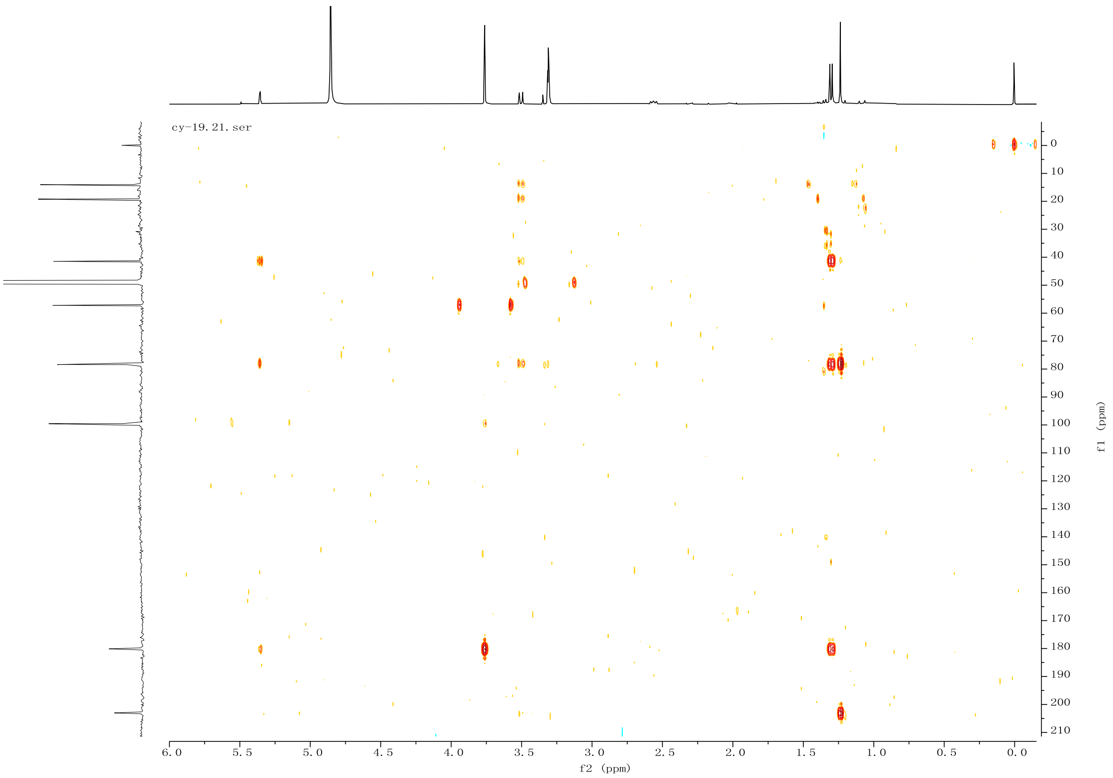

Figure S47 HMBC spectrum of **10**

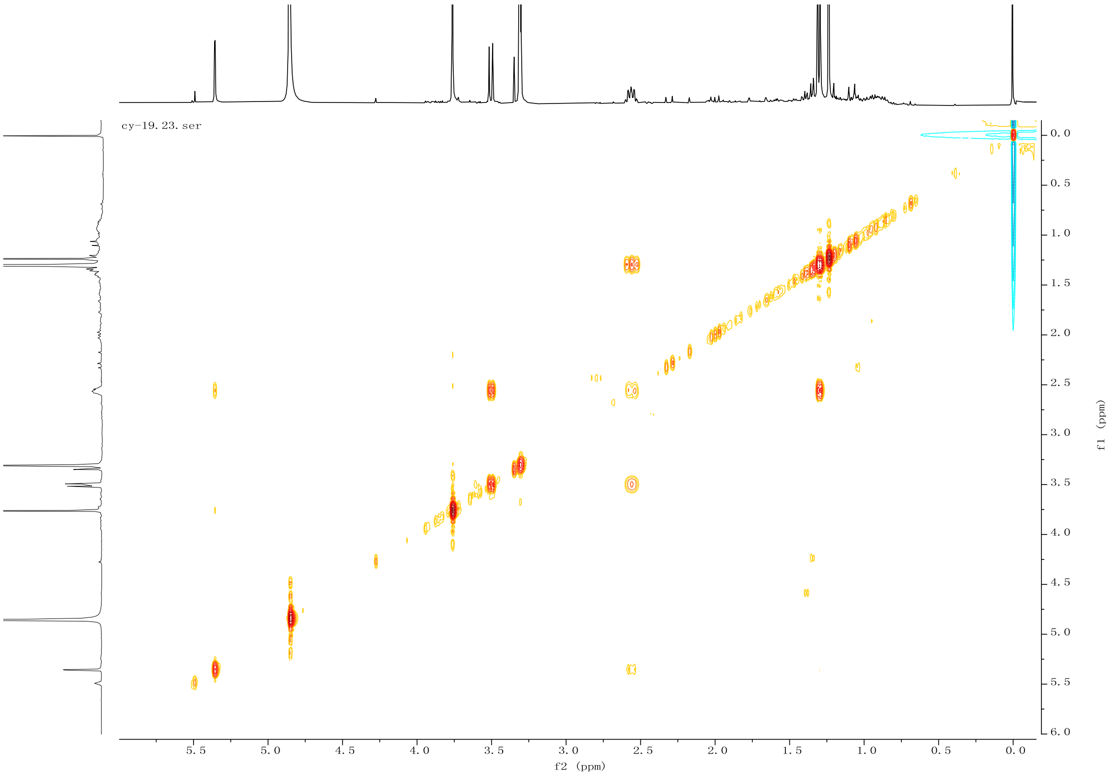

Figure S48 COSY spectrum of **10**

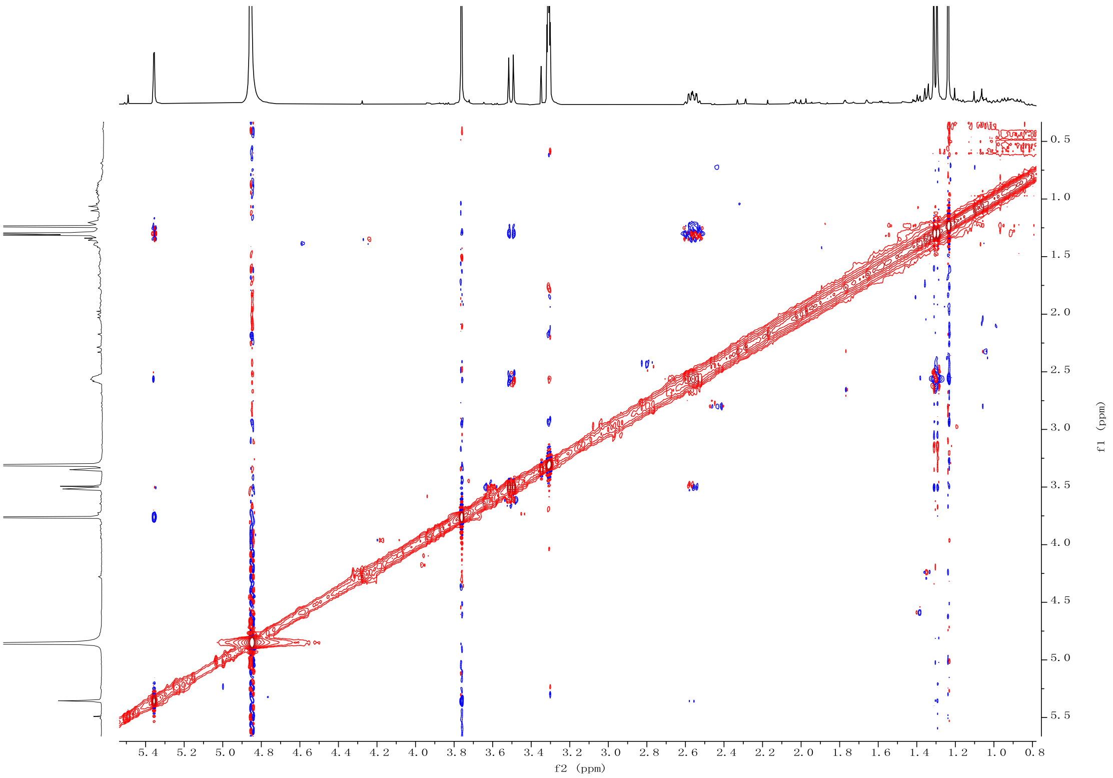

Figure S49 NOESY spectrum of **10**

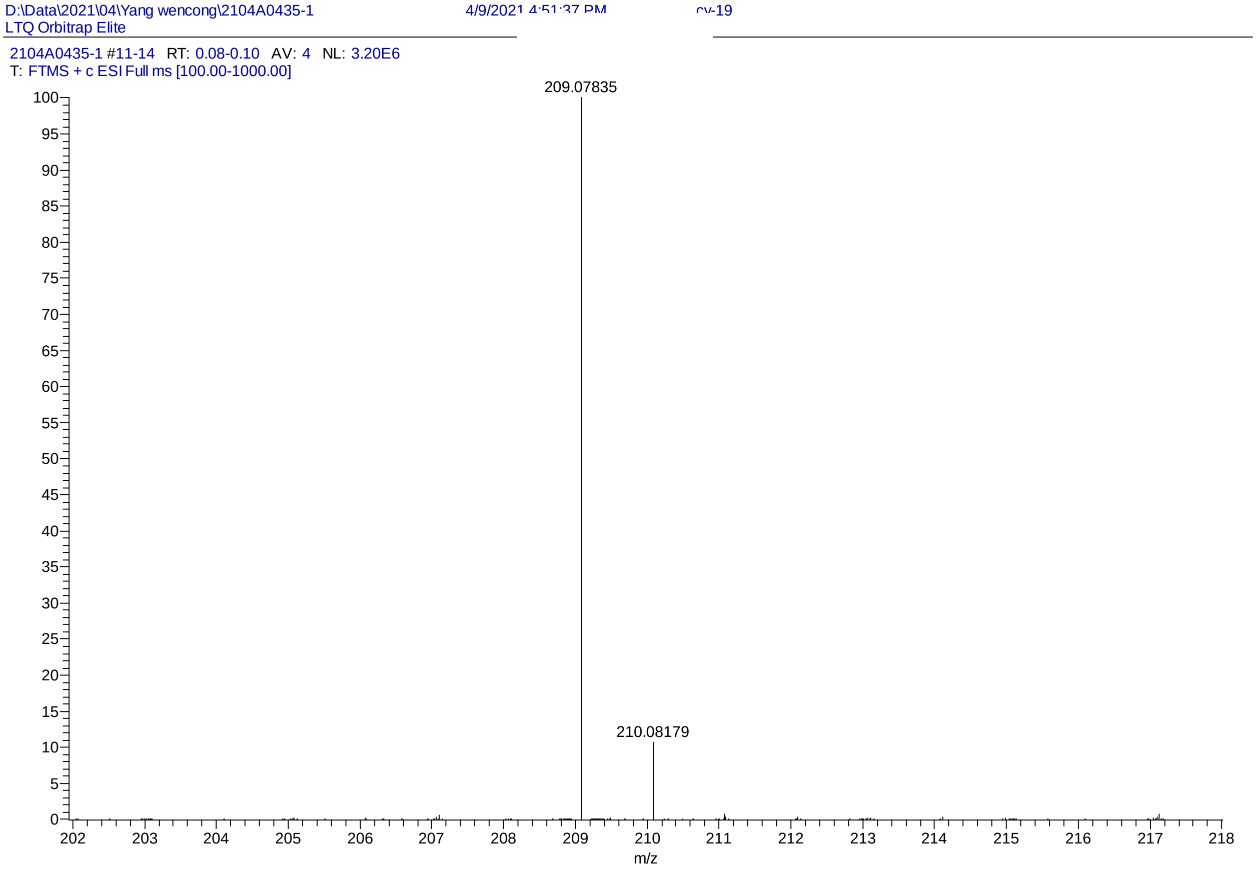

Figure S50 HR-ESI-MS spectrum of **10**

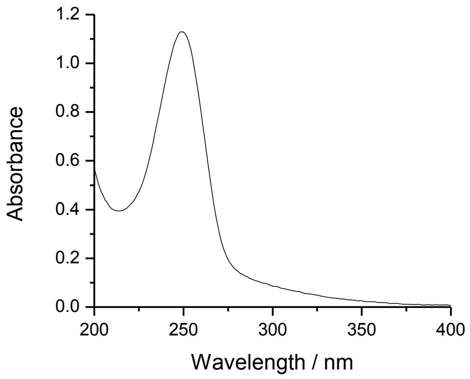

Figure S51 UV spectrum of **10**

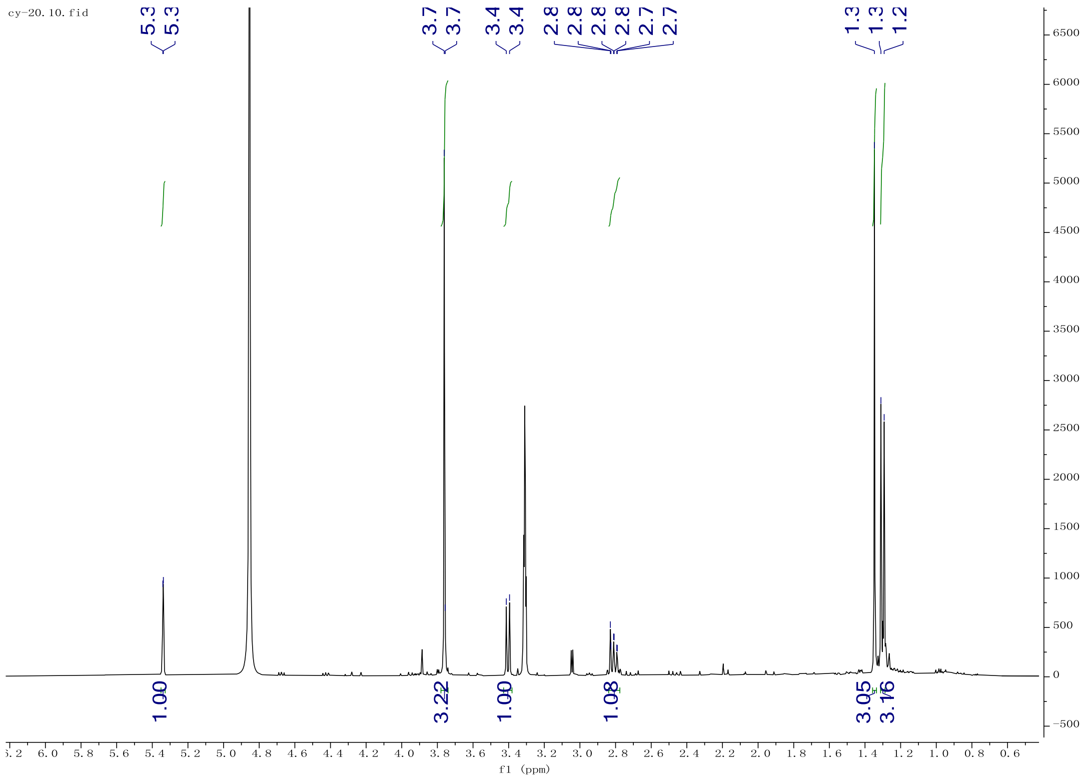

Figure S52 ^{1}H NMR of **11**

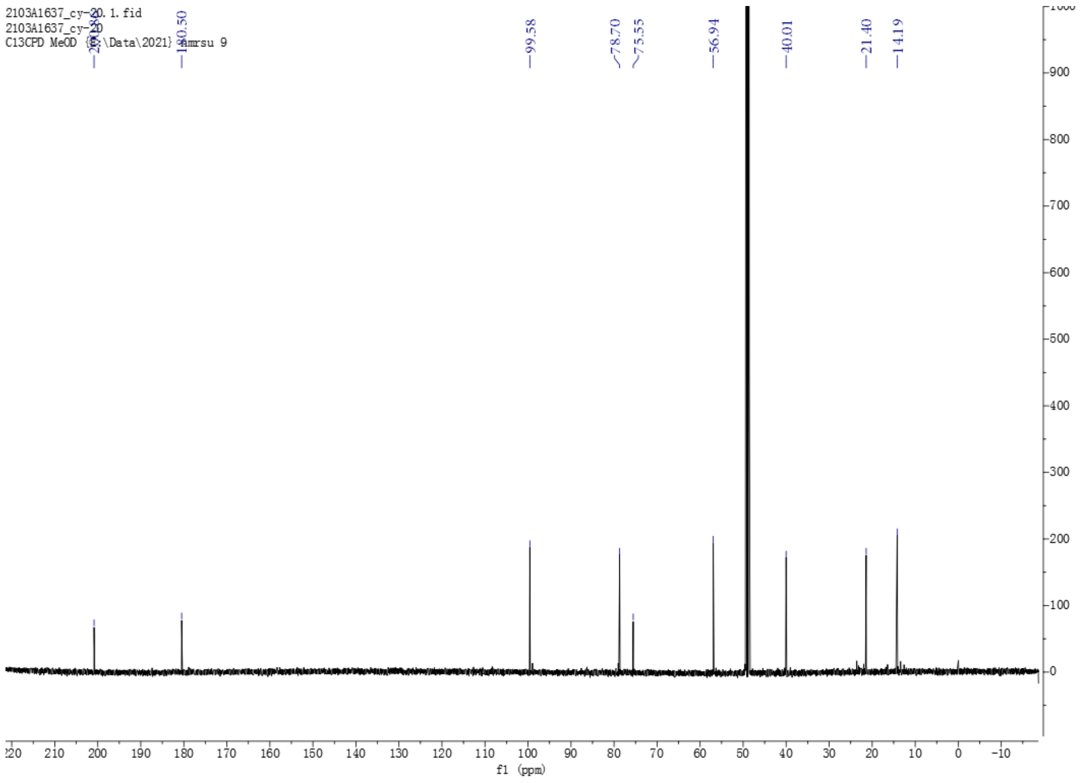

Figure S53 ^{13}C NMR of **11**

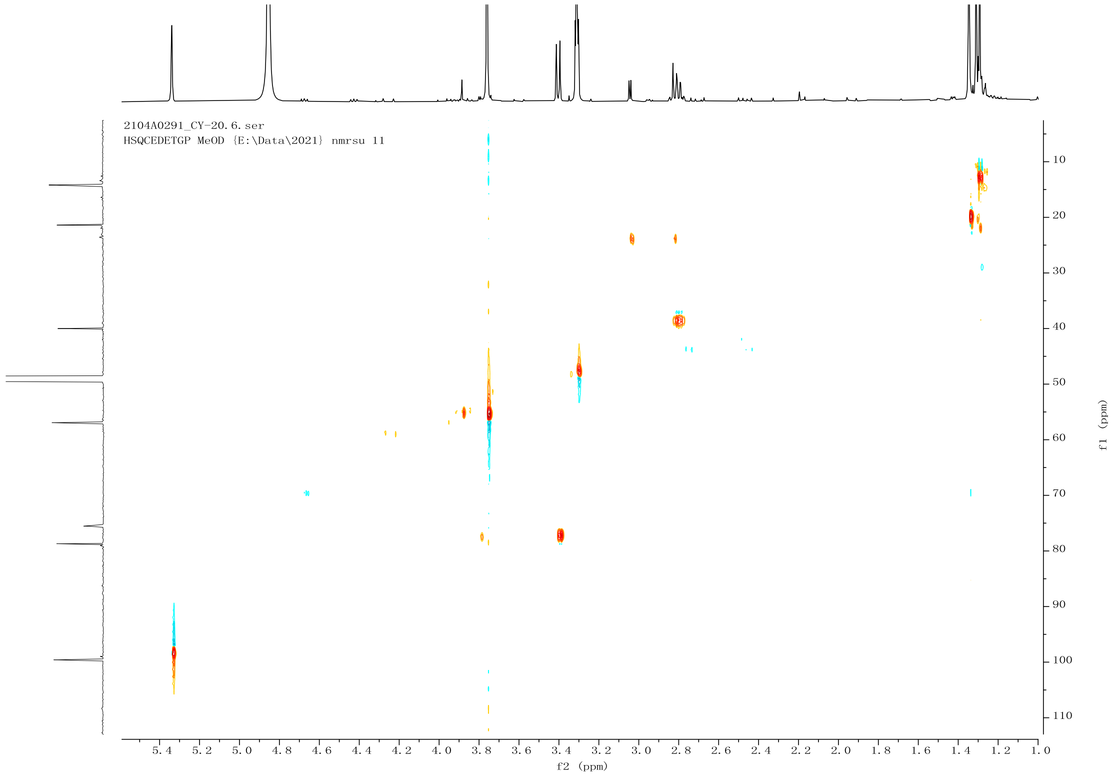

Figure S54 HSQC spectrum of **11**

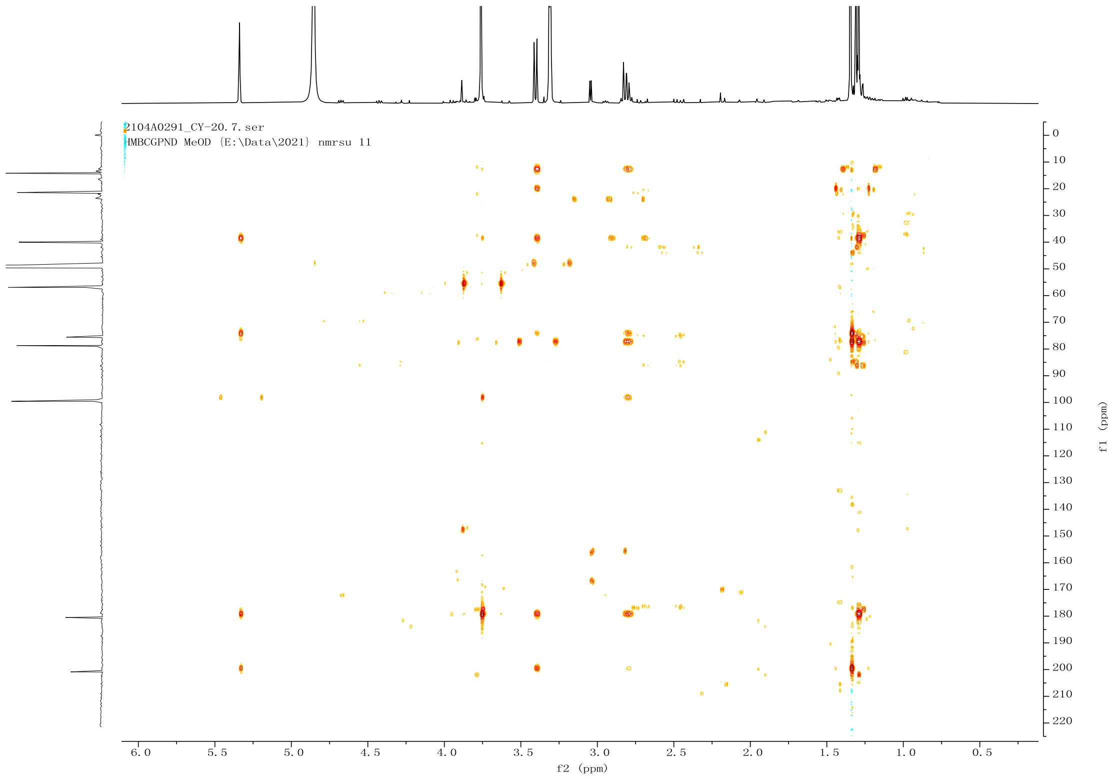

Figure S55 HMBC spectrum of **11**

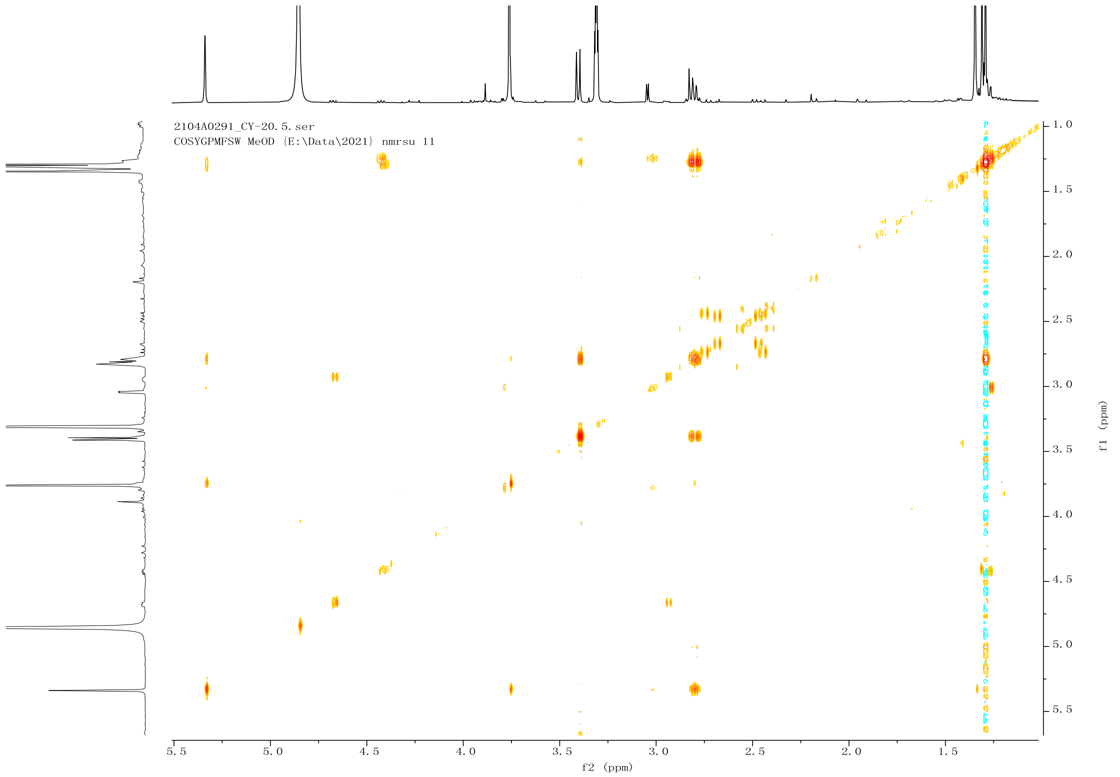

Figure S56 COSY spectrum of **11**

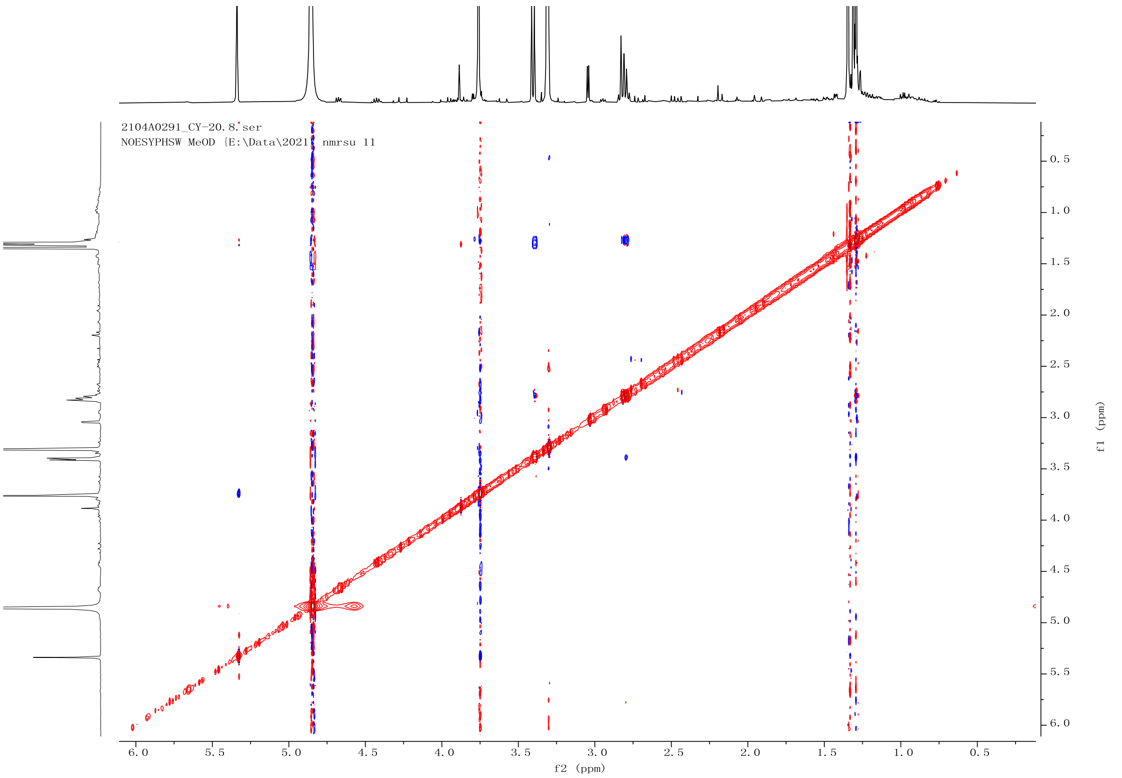

Figure S57 NOESY spectrum of **11**

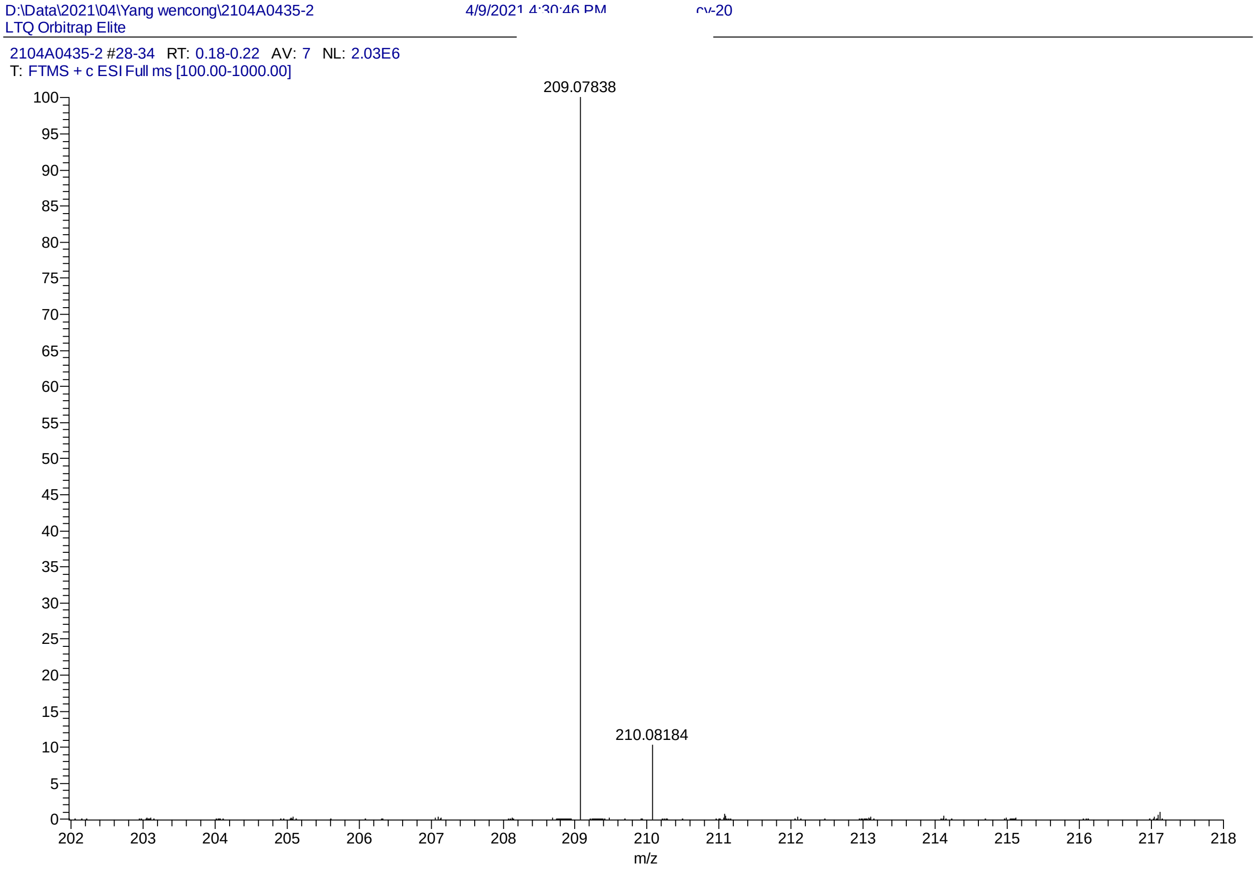

Figure S58 HR-ESI-MS spectrum of **11**

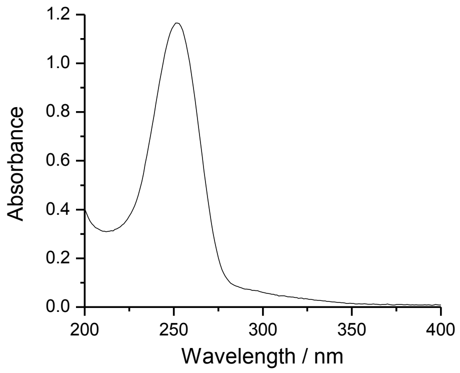

Figure S59 UV of **11**

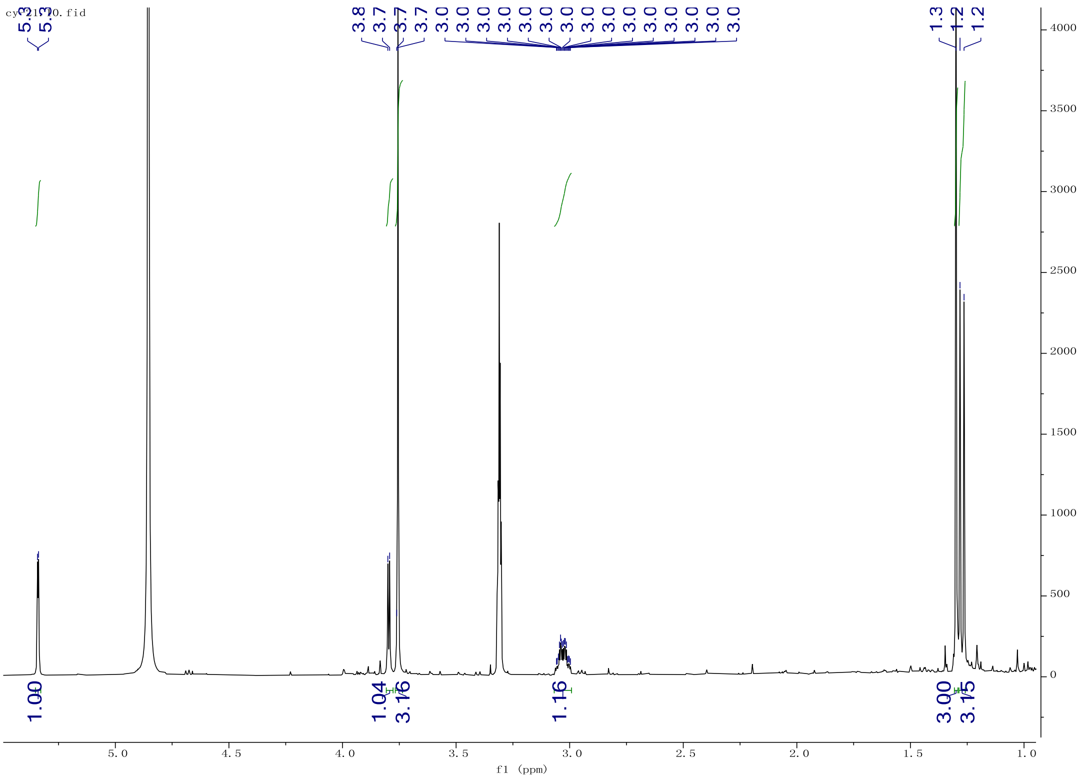

Figure S60 ^{1}H NMR of **12**

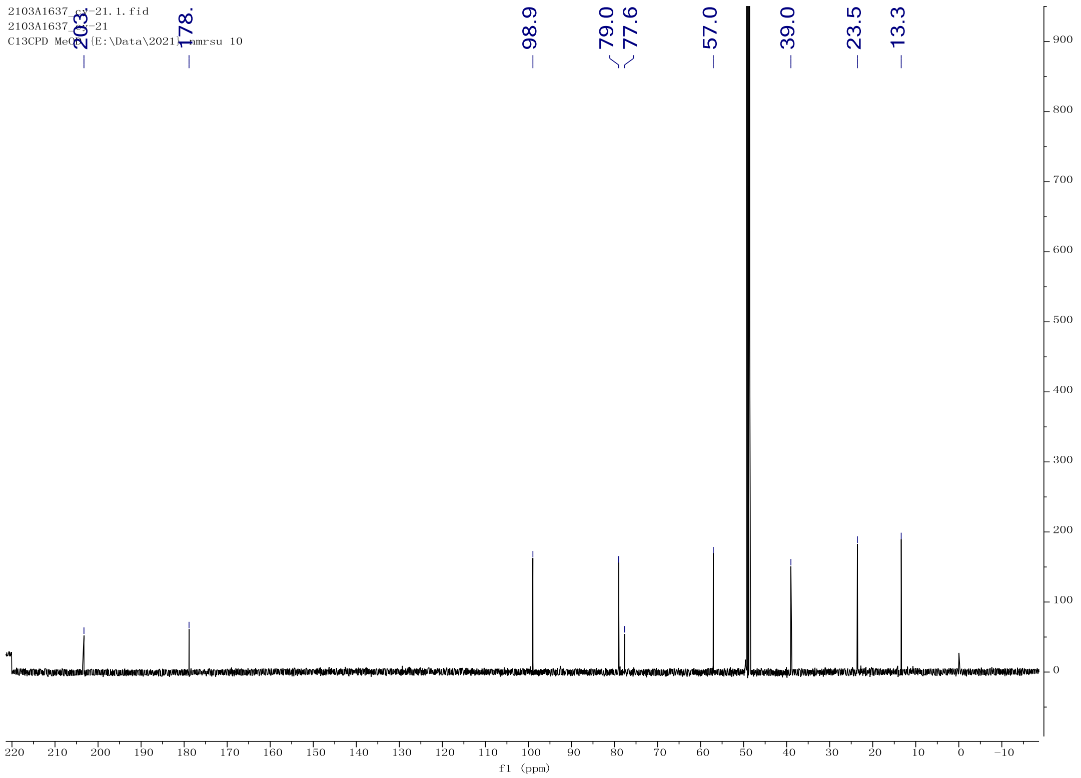

Figure S61 ^{13}C NMR of **12**

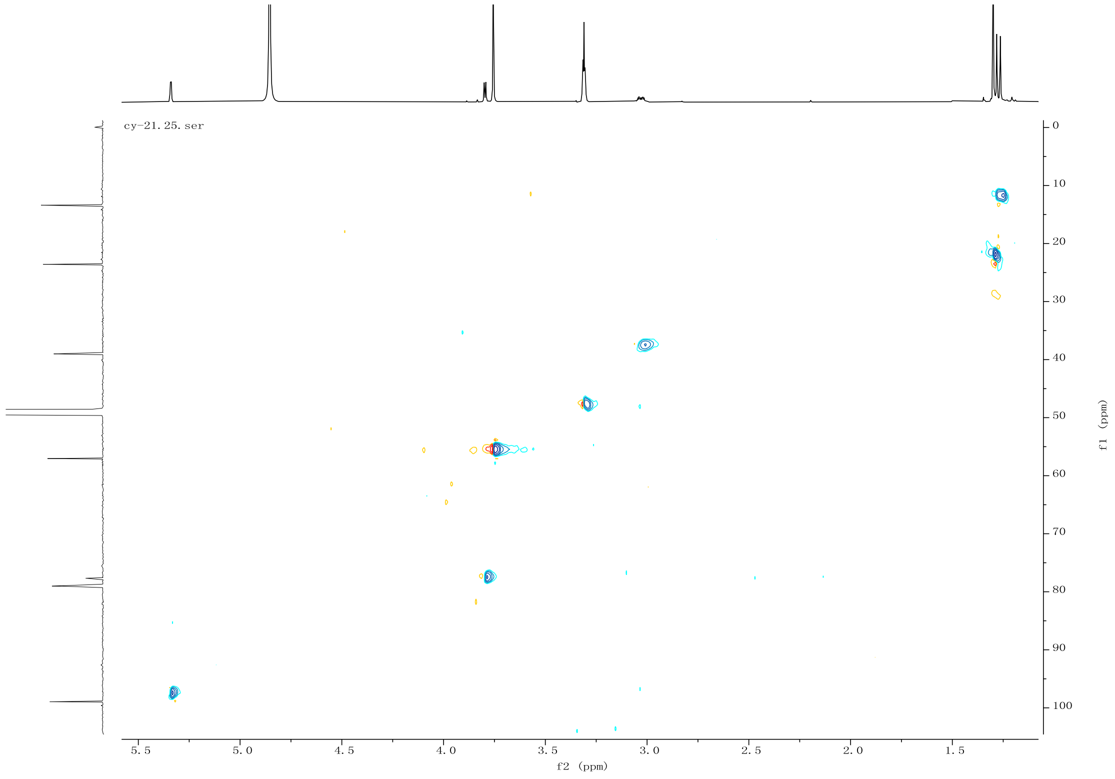

Figure S62 HSQC spectrum of **12**

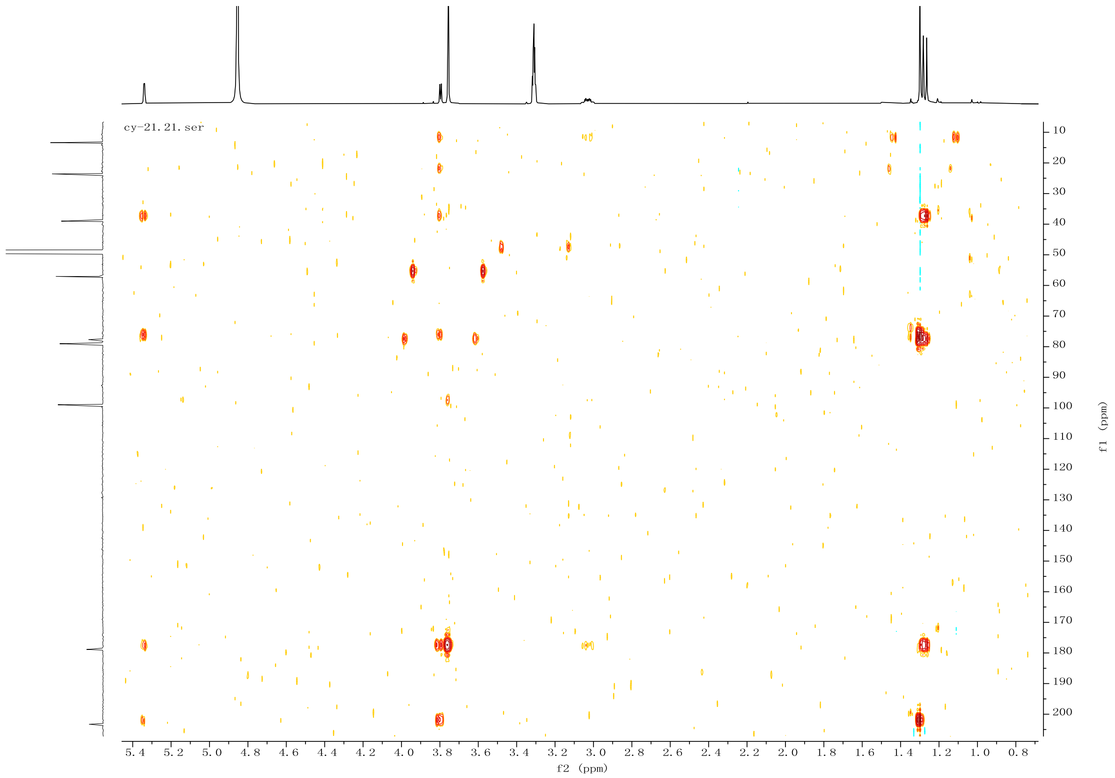

Figure S63 HMBC spectrum of **12**

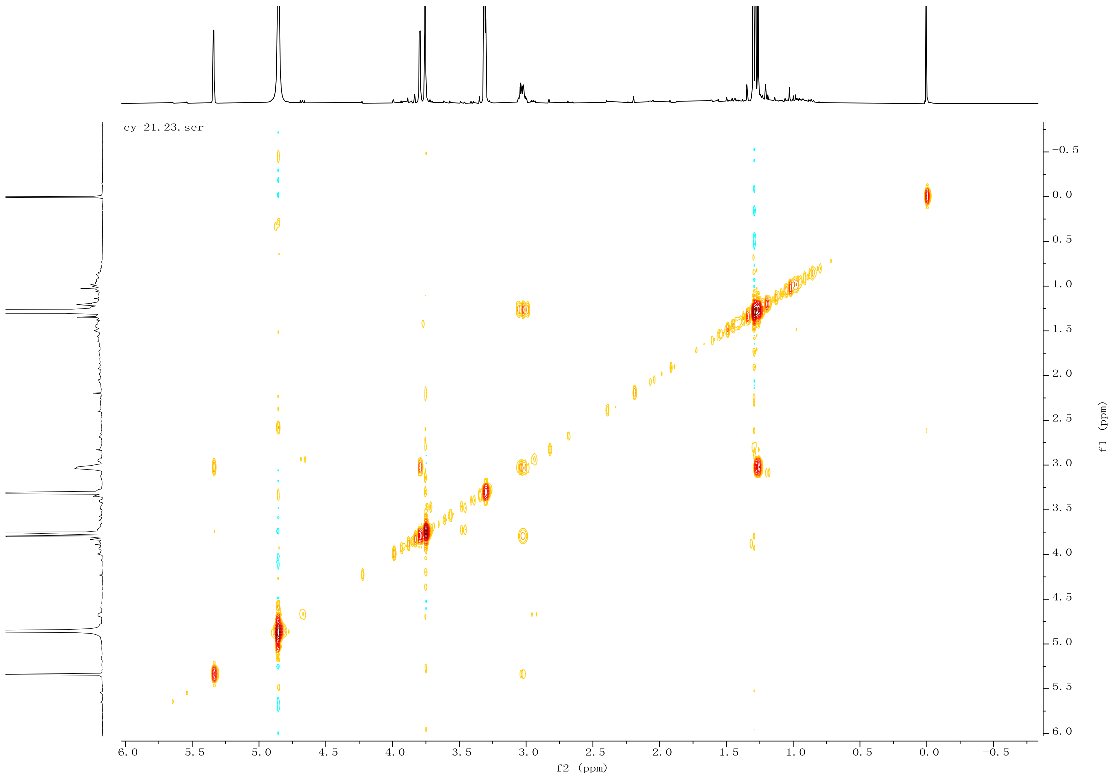

Figure S64 COSY spectrum of **12**

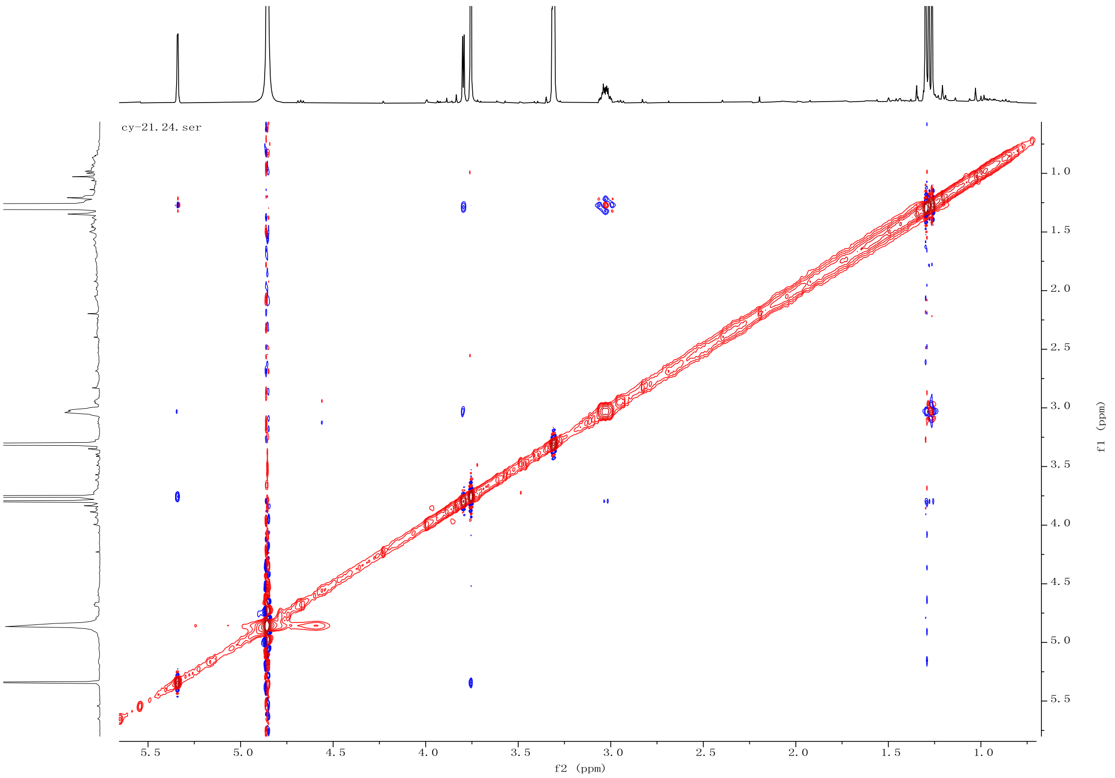

Figure S65 NOESY spectrum of **12**

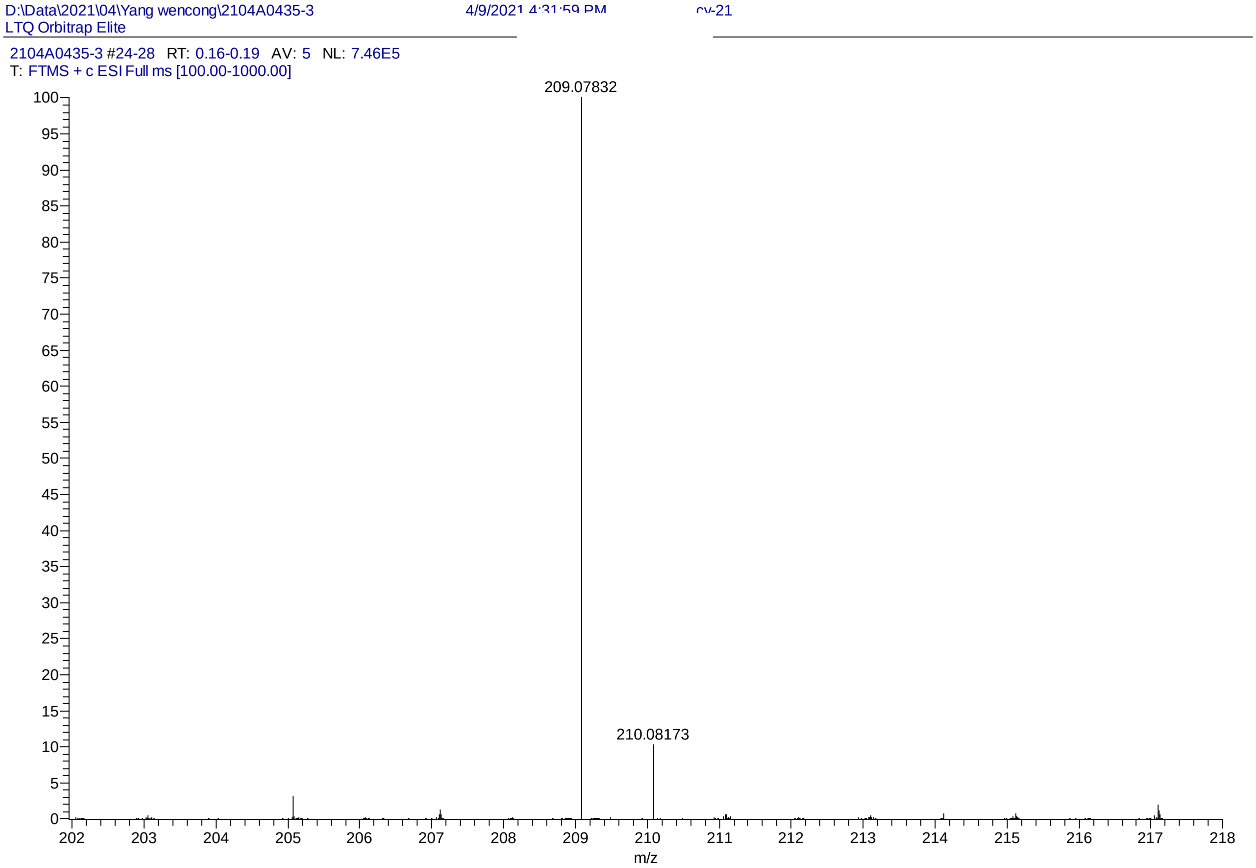

Figure S66 HR-ESI-MS spectrum of **12**

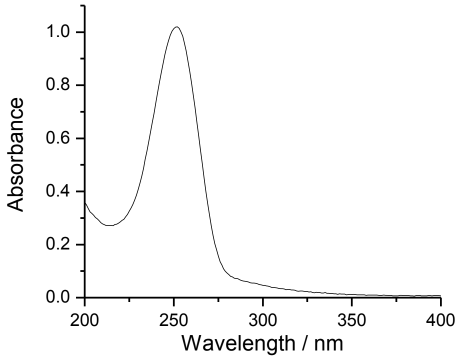

Figure S67 UV spectrum of **12**

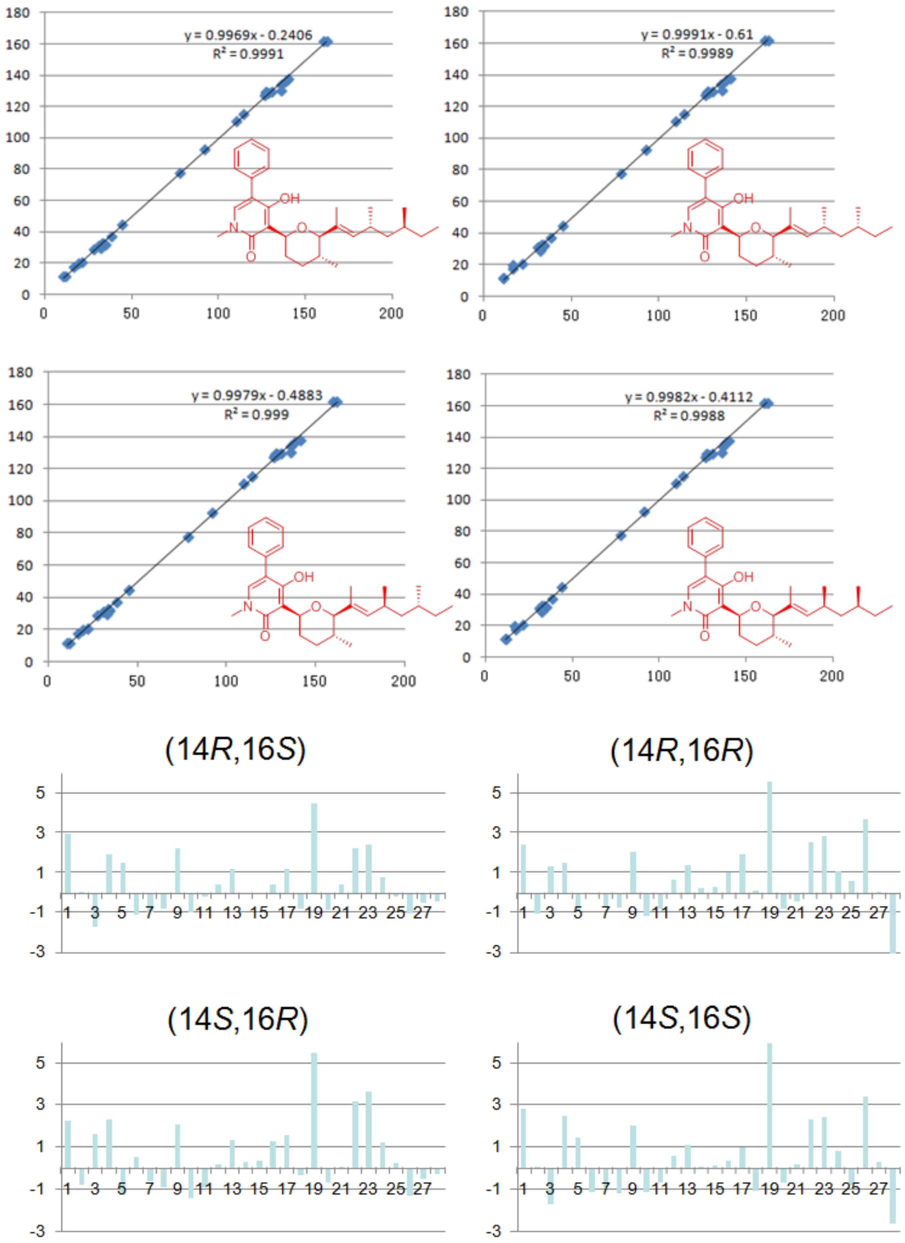

Figure S68 Comparison of the experimental ^{13}C NMR data and mean absolute error

of the 2,4,6-trimethyloct-2-ene side chain for compound **1** and the calculated chemical shifts for four 2,4,6-trimethyloct-2-ene side chain diastereomers (14*R*,16*S*-**1**, 14*R*,16*R*-**1** 14*S*,16*R*-**1**, and 14*S*, 16*S*-**1**).

Functional	Solvent?		Basis Set	
mPW1PW91	PCM		6-31+G(d,p)	
	Isomer 1	Isomer 2	Isomer 3	Isomer 4
sDP4+ (H data)	99.91%	0.06%	0.03%	0.00%
sDP4+ (C data)	5.11%	79.26%	14.07%	1.55%
sDP4+ (all data)	98.96%	0.97%	0.07%	0.00%
uDP4+ (H data)	99.71%	0.21%	0.05%	0.03%
uDP4+ (C data)	63.79%	1.42%	32.49%	2.30%
uDP4+ (all data)	99.97%	0.00%	0.02%	0.00%
DP4+ (H data)	100.00%	0.00%	0.00%	0.00%
DP4+ (C data)	36.25%	12.52%	50.83%	0.40%
DP4+ (all data)	100.00%	0.00%	0.00%	0.00%

Figure S69 DP4+ analysis of **1**.

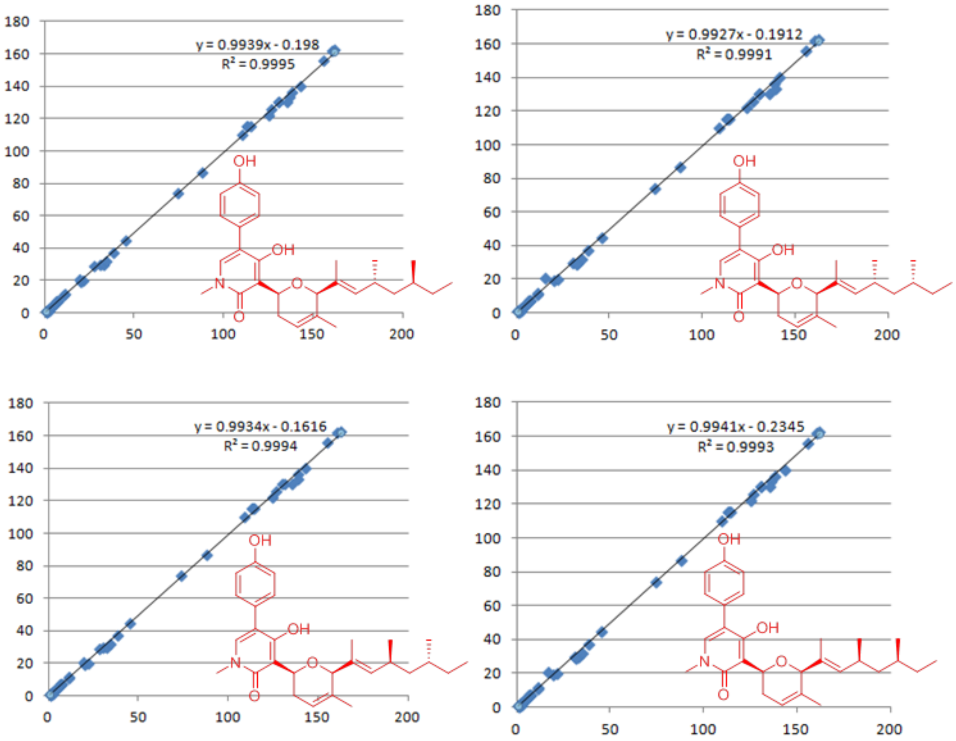

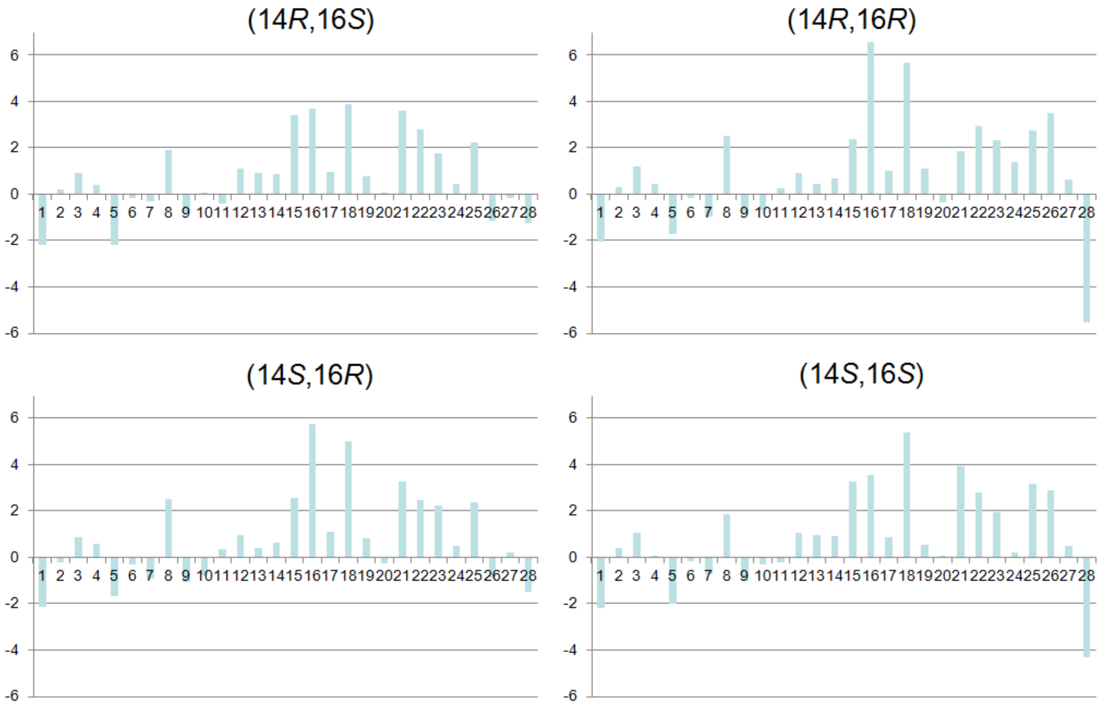

Figure S70 Comparison of the experimental ^{13}C NMR data and mean absolute error of the 2,4,6-trimethyloct-2-ene side chain for compound **2** and the calculated chemical shifts for four 2,4,6-trimethyloct-2-ene side chain diastereomers (14*R*,16*S*-**2**, 14*R*,16*R*-**2** 14*S*,16*R*-**2**, and 14*S*, 16*S*-**2**).

A	B	C	D	E	F
Functional		Solvent?		Basis Set	
mPW1PW91		PCM		6-31+G(d,p)	
		Isomer 1	Isomer 2	Isomer 3	Isomer 4
sDP4+ (H data)		15.04%	28.06%	0.06%	56.84%
sDP4+ (C data)		82.55%	0.13%	15.99%	1.33%
sDP4+ (all data)		93.93%	0.28%	0.08%	5.71%
uDP4+ (H data)		1.41%	97.14%	0.01%	1.44%
uDP4+ (C data)		29.90%	9.50%	5.36%	55.25%
uDP4+ (all data)		4.03%	88.33%	0.01%	7.64%
DP4+ (H data)		0.75%	96.35%	0.00%	2.90%
DP4+ (C data)		93.90%	0.05%	3.26%	2.79%
DP4+ (all data)		84.71%	5.54%	0.00%	9.76%

Figure S71 DP4+ analysis of **2**.

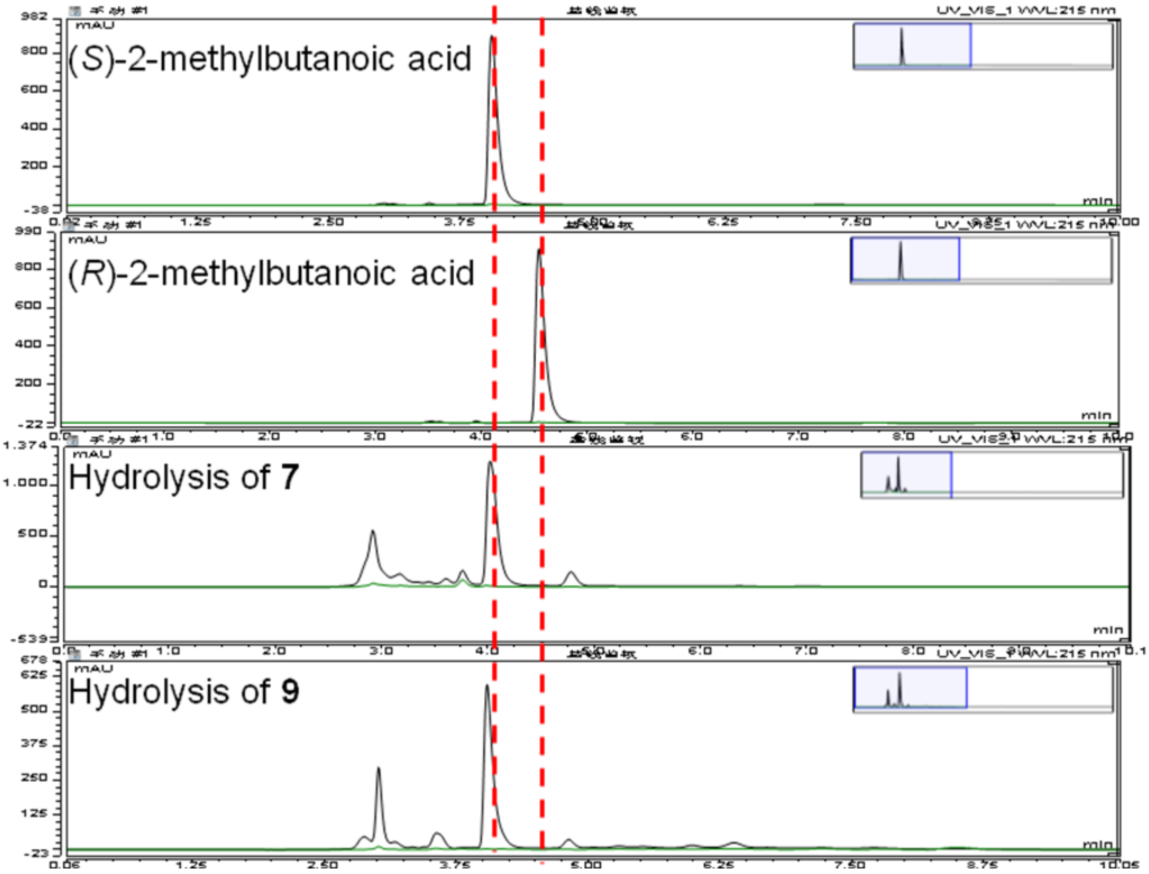

Figure S72 Hydrolysis of **7** and **9** compared with the standard through HPLC chiral column.

Table S1 Gibbs free energies and Boltzmann-population of low-energy conformers of 7*S*, 10*R*, 11*R*, 13*E*, 14*R*, 16*S* -**1**.

Conformers of Compound	In MeOH	
	ΔG[a]	P(%)[b]
**1a**	0.00	67.53%
**1b**	0.66	22.16%
**1c**	1.62	4.35%
**1d**	1.59	4.60%
**1e**	2.31	1.36%

Table S2 Cartesian coordinates for the low-energy optimized conformers of 7*S*, 10*R*, 11*R*, 13*E*, 14*R*, 16*S* -**1** at B3LYP/6-31+G (d, p) level of theory in MeOH.

1a

Standard orientation:

Center Number	Atomic Number	Atomic Type	Coordinates (Angstroms) X	Y	Z
1	6	0	6.171897	0.562445	-0.498946
2	6	0	-5.995176	2.857260	1.677455
3	6	0	-5.417181	1.623282	1.372857
4	6	0	-4.636286	1.472464	0.219231
5	6	0	-4.434777	2.575755	-0.619706
6	6	0	-5.013534	3.809720	-0.313005
7	6	0	-5.795266	3.948797	0.833107
8	6	0	-4.034159	0.165960	-0.115141
9	6	0	-4.857311	-0.780335	-0.596797
10	7	0	-4.398828	-2.035696	-0.951010
11	6	0	-3.057354	-2.394538	-0.848823
12	6	0	-2.123553	-1.365028	-0.280889
13	6	0	-2.622890	-0.153364	0.029706
14	6	0	-5.355163	-2.988531	-1.492371
15	6	0	-0.684868	-1.810504	-0.152125
16	8	0	-1.846787	0.879693	0.515326
17	6	0	-0.494640	-2.774388	1.026060
18	6	0	0.970795	-3.180411	1.156204
19	6	0	1.879854	-1.945168	1.225606
20	6	0	1.554671	-0.997109	0.049802
21	8	0	0.159315	-0.659754	0.060668
22	6	0	2.323166	0.316966	0.082105
23	6	0	3.348424	-2.369456	1.248318
24	6	0	2.130320	1.168676	1.311483
25	6	0	3.114571	0.663381	-0.956087
26	6	0	3.989307	1.889962	-1.090391

27	6	0	3.392133	2.819458	-2.151747
28	6	0	5.437364	1.513282	-1.475313
29	6	0	6.301476	1.172799	0.909906
30	6	0	6.997170	0.262537	1.913845
31	6	0	7.543108	0.210831	-1.091347
32	8	0	-2.620004	-3.491247	-1.197312
33	1	0	5.603616	-0.372228	-0.423478
34	1	0	-6.596428	2.968403	2.575308
35	1	0	-5.571243	0.781771	2.044194
36	1	0	-3.818171	2.479716	-1.509998
37	1	0	-4.848001	4.662145	-0.967274
38	1	0	-6.240701	4.910356	1.071732
39	1	0	-5.919890	-0.596503	-0.735585
40	1	0	-6.199519	-3.085405	-0.801876
41	1	0	-5.712115	-2.620027	-2.459346
42	1	0	-4.909866	-3.977598	-1.636088
43	1	0	-0.373698	-2.279503	-1.094084
44	1	0	-0.911818	0.586355	0.454023
45	1	0	-0.801824	-2.283516	1.959614
46	1	0	-1.109526	-3.673889	0.916011
47	1	0	1.102228	-3.797309	2.053216
48	1	0	1.249960	-3.803866	0.296798
49	1	0	1.651289	-1.437883	2.172523
50	1	0	1.765186	-1.516313	-0.895644
51	1	0	3.533709	-3.080224	2.060544
52	1	0	3.637274	-2.849181	0.307243
53	1	0	4.008430	-1.511685	1.408694
54	1	0	2.405057	2.214207	1.153691
55	1	0	1.080973	1.176930	1.622063
56	1	0	2.728587	0.779591	2.140757

57	1	0	3.161737	-0.009623	-1.812808
58	1	0	4.025961	2.455497	-0.155475
59	1	0	2.376275	3.125314	-1.878983
60	1	0	3.995696	3.728007	-2.254082
61	1	0	3.346304	2.333748	-3.133714
62	1	0	5.427722	1.050903	-2.472086
63	1	0	6.024379	2.436750	-1.576888
64	1	0	6.841186	2.125791	0.857976
65	1	0	5.306338	1.393965	1.309502
66	1	0	8.056263	0.132129	1.672608
67	1	0	6.941896	0.699937	2.916433
68	1	0	6.522420	-0.722899	1.948668
69	1	0	8.040544	-0.571303	-0.510400
70	1	0	7.439899	-0.168462	-2.114350
71	1	0	8.198873	1.087790	-1.119796

1**b**

Standard orientation:

Center Number	Atomic Number	Atomic Type	Coordinates (Angstroms) X	Y	Z
1	6	0	-6.206685	0.218943	0.412042
2	6	0	5.895274	3.069869	-1.548385
3	6	0	5.365993	1.804237	-1.285937
4	6	0	4.577012	1.589332	-0.147334
5	6	0	4.318920	2.660406	0.717448
6	6	0	4.848685	3.924366	0.454013
7	6	0	5.638380	4.127823	-0.677014
8	6	0	4.025471	0.250572	0.142916

9	6	0	4.881890	-0.674083	0.610262
10	7	0	4.471972	-1.956646	0.923381
11	6	0	3.148870	-2.368182	0.793643
12	6	0	2.180176	-1.362900	0.240149
13	6	0	2.632214	-0.122682	-0.029270
14	6	0	5.460520	-2.884084	1.452071
15	6	0	0.763203	-1.863703	0.079789
16	8	0	1.818705	0.889413	-0.498004
17	6	0	0.627429	-2.802529	-1.126912
18	6	0	-0.817650	-3.267014	-1.287552
19	6	0	-1.776833	-2.069176	-1.335573
20	6	0	-1.507045	-1.140266	-0.131541
21	8	0	-0.125819	-0.743923	-0.113784
22	6	0	-2.330256	0.140638	-0.140106
23	6	0	-3.225968	-2.554495	-1.387938
24	6	0	-2.153012	1.035854	-1.340110
25	6	0	-3.153321	0.422173	0.893066
26	6	0	-4.082677	1.605984	1.045541
27	6	0	-3.528848	2.546078	2.120523
28	6	0	-5.511692	1.155988	1.425561
29	6	0	-6.313375	0.786640	-1.016962
30	6	0	-7.047621	2.114855	-1.137035
31	6	0	-7.585516	-0.184411	0.950466
32	8	0	2.752967	-3.490719	1.106122
33	1	0	-5.620025	-0.706167	0.342666
34	1	0	6.502220	3.230405	-2.434836
35	1	0	5.563343	0.989383	-1.977968
36	1	0	3.695921	2.513553	1.597184
37	1	0	4.639857	4.750270	1.127791
38	1	0	6.046566	5.112870	-0.884424

39	1	0	5.934423	-0.450157	0.769065
40	1	0	6.317702	-2.926066	0.771732
41	1	0	5.789239	-2.527570	2.434168
42	1	0	5.055307	-3.893818	1.562900
43	1	0	0.459668	-2.370726	1.004431
44	1	0	0.896107	0.555483	-0.457544
45	1	0	0.925585	-2.273770	-2.042355
46	1	0	1.279164	-3.677634	-1.032227
47	1	0	-0.911736	-3.863535	-2.202261
48	1	0	-1.079707	-3.924278	-0.448930
49	1	0	-1.558773	-1.527375	-2.265730
50	1	0	-1.707124	-1.692833	0.797283
51	1	0	-3.370883	-3.250444	-2.222015
52	1	0	-3.504248	-3.071961	-0.463639
53	1	0	-3.920893	-1.721852	-1.533215
54	1	0	-2.711131	0.640630	-2.194763
55	1	0	-2.485714	2.060765	-1.161278
56	1	0	-1.097971	1.106881	-1.624421
57	1	0	-3.184488	-0.276500	1.730504
58	1	0	-4.147747	2.178618	0.116991
59	1	0	-2.526773	2.899857	1.852327
60	1	0	-4.172886	3.425220	2.233543
61	1	0	-3.462695	2.049534	3.095055
62	1	0	-5.476474	0.647583	2.398942
63	1	0	-6.133696	2.047065	1.581860
64	1	0	-5.309999	0.901954	-1.442108
65	1	0	-6.821116	0.051359	-1.654153
66	1	0	-8.078421	2.038866	-0.779753
67	1	0	-6.542730	2.906974	-0.577215
68	1	0	-7.083715	2.425145	-2.186786

69	1	0	-7.485043	-0.716439	1.902591
70	1	0	-8.224953	0.687242	1.123007
71	1	0	-8.098022	-0.851587	0.250338

1**c**

Standard orientation:

Center	Atomic	Atomic	Coordinates (Angstroms)		
Number	Number	Type	X	Y	Z
1	6	0	-2.448814	2.799775	-0.363496
2	6	0	4.377782	3.245276	-1.518706
3	6	0	4.058952	1.894059	-1.359875
4	6	0	3.439384	1.446722	-0.185034
5	6	0	3.136824	2.372749	0.822475
6	6	0	3.453709	3.722958	0.660611
7	6	0	4.075905	4.157002	-0.509189
8	6	0	3.103882	0.019385	-0.006359
9	6	0	4.123061	-0.832403	0.200150
10	7	0	3.916766	-2.186014	0.391788
11	6	0	2.645366	-2.753589	0.400866
12	6	0	1.492900	-1.825968	0.147806
13	6	0	1.751951	-0.514258	-0.020546
14	6	0	5.077853	-3.030513	0.631796
15	6	0	0.131730	-2.482263	0.140758
16	8	0	0.765517	0.428449	-0.230147
17	6	0	-0.119431	-3.245024	-1.164969
18	6	0	-1.527587	-3.831304	-1.183294
19	6	0	-2.579539	-2.746822	-0.901802
20	6	0	-2.205814	-1.989408	0.393365

21	8	0	-0.875200	-1.464012	0.278895
22	6	0	-3.050318	-0.784748	0.796279
23	6	0	-3.970093	-3.380150	-0.825435
24	6	0	-3.014343	-0.516254	2.281695
25	6	0	-3.708933	-0.012043	-0.095572
26	6	0	-4.500852	1.253118	0.153600
27	6	0	-5.962542	1.012604	-0.238123
28	6	0	-3.927217	2.438944	-0.655703
29	6	0	-2.252811	3.279540	1.088508
30	6	0	-0.801620	3.576396	1.446803
31	6	0	-1.989628	3.861172	-1.371577
32	8	0	2.434394	-3.948662	0.603131
33	1	0	-1.828200	1.911162	-0.526110
34	1	0	4.853596	3.585747	-2.434597
35	1	0	4.286391	1.195596	-2.160441
36	1	0	2.640252	2.045133	1.732898
37	1	0	3.208600	4.433648	1.444705
38	1	0	4.318539	5.209517	-0.635899
39	1	0	5.154994	-0.491575	0.233430
40	1	0	5.800565	-2.888045	-0.177146
41	1	0	5.533320	-2.742384	1.585455
42	1	0	4.810030	-4.089445	0.677553
43	1	0	0.046807	-3.150520	1.007569
44	1	0	-0.098086	-0.020323	-0.100420
45	1	0	-0.018984	-2.562699	-2.019049
46	1	0	0.611107	-4.049266	-1.305462
47	1	0	-1.717459	-4.298248	-2.157177
48	1	0	-1.597002	-4.627235	-0.429711
49	1	0	-2.548975	-2.057205	-1.754370
50	1	0	-2.225321	-2.709603	1.224608

51	1	0	-4.189002	-3.939997	-1.740906
52	1	0	-4.042038	-4.075010	0.018819
53	1	0	-4.752384	-2.625861	-0.708336
54	1	0	-3.345417	-1.404508	2.830890
55	1	0	-1.992655	-0.277007	2.596002
56	1	0	-3.658010	0.303542	2.600996
57	1	0	-3.648022	-0.271591	-1.150883
58	1	0	-4.502252	1.527858	1.210203
59	1	0	-6.394375	0.191430	0.344492
60	1	0	-6.566065	1.907348	-0.048615
61	1	0	-6.061348	0.760608	-1.300333
62	1	0	-4.027684	2.213428	-1.725981
63	1	0	-4.550451	3.325197	-0.473150
64	1	0	-2.856561	4.175814	1.272048
65	1	0	-2.607127	2.512502	1.783757
66	1	0	-0.719777	3.804325	2.513716
67	1	0	-0.162346	2.713845	1.235861
68	1	0	-0.417129	4.439954	0.896286
69	1	0	-2.180734	3.531801	-2.398650
70	1	0	-2.516042	4.808937	-1.217171
71	1	0	-0.914329	4.049306	-1.292596

1d

Standard orientation:

Center Number	Atomic Number	Atomic Type	Coordinates (Angstroms) X	Y	Z
1	6	0	-1.782853	2.991962	0.413738
2	6	0	4.614193	2.728450	-1.870819

3	6	0	4.200771	1.432017	-1.557877
4	6	0	3.438837	1.189233	-0.407230
5	6	0	3.089859	2.265911	0.418497
6	6	0	3.502383	3.561647	0.104249
7	6	0	4.267108	3.792026	-1.038631
8	6	0	3.001814	-0.181807	-0.064242
9	6	0	3.949504	-1.052983	0.320197
10	7	0	3.644546	-2.353047	0.679132
11	6	0	2.339886	-2.838785	0.687610
12	6	0	1.266333	-1.892085	0.236114
13	6	0	1.621305	-0.634523	-0.093187
14	6	0	4.731967	-3.220155	1.109209
15	6	0	-0.133153	-2.462383	0.236313
16	8	0	0.710456	0.325083	-0.489710
17	6	0	-0.364640	-3.404668	-0.950912
18	6	0	-1.803866	-3.915660	-0.958874
19	6	0	-2.803268	-2.751690	-0.902921
20	6	0	-2.442838	-1.815394	0.271534
21	8	0	-1.079545	-1.379904	0.151559
22	6	0	-3.289424	-0.550339	0.336813
23	6	0	-4.232095	-3.286068	-0.812146
24	6	0	-4.204169	-0.479241	1.529079
25	6	0	-3.166352	0.419966	-0.595973
26	6	0	-3.842557	1.770573	-0.649180
27	6	0	-4.782866	1.813535	-1.858105
28	6	0	-2.802153	2.909737	-0.749026
29	6	0	-2.471510	3.303296	1.757043
30	6	0	-1.515750	3.332093	2.942283
31	6	0	-0.717393	4.040657	0.068592
32	8	0	2.039911	-3.977588	1.041982

33	1	0	-1.268640	2.028236	0.505755
34	1	0	5.201774	2.909238	-2.767364
35	1	0	4.467122	0.613112	-2.222495
36	1	0	2.482985	2.098913	1.306404
37	1	0	3.220414	4.390802	0.748106
38	1	0	4.583655	4.802100	-1.285896
39	1	0	4.997738	-0.770363	0.375325
40	1	0	5.517903	-3.219665	0.347051
41	1	0	5.135251	-2.839499	2.052507
42	1	0	4.396075	-4.249702	1.261513
43	1	0	-0.309632	-2.981005	1.188184
44	1	0	-0.184325	-0.049551	-0.337899
45	1	0	-0.184475	-2.867504	-1.891486
46	1	0	0.322230	-4.256228	-0.926552
47	1	0	-1.973690	-4.517047	-1.859914
48	1	0	-1.954420	-4.580150	-0.098527
49	1	0	-2.702235	-2.198918	-1.846394
50	1	0	-2.534798	-2.356480	1.222430
51	1	0	-4.437226	-3.982687	-1.631920
52	1	0	-4.398020	-3.817941	0.131119
53	1	0	-4.965558	-2.475784	-0.880408
54	1	0	-3.614971	-0.403568	2.449170
55	1	0	-4.894898	0.364915	1.502906
56	1	0	-4.817962	-1.384548	1.587246
57	1	0	-2.466555	0.246015	-1.415492
58	1	0	-4.457599	1.945367	0.236377
59	1	0	-5.544282	1.028612	-1.791002
60	1	0	-5.301691	2.776547	-1.909821
61	1	0	-4.237568	1.675224	-2.799382
62	1	0	-2.249132	2.797108	-1.692488

63	1	0	-3.333511	3.868058	-0.823300
64	1	0	-2.996904	4.265147	1.695920
65	1	0	-3.230328	2.543090	1.969168
66	1	0	-0.932760	2.407885	2.999572
67	1	0	-0.825440	4.177850	2.883951
68	1	0	-2.080380	3.433745	3.875860
69	1	0	-0.265889	3.828101	-0.907089
70	1	0	-1.149438	5.046069	0.030237
71	1	0	0.094328	4.043191	0.801171

1**e**

Standard orientation:

Center Number	Atomic Number	Atomic Type	Coordinates (Angstroms) X	Y	Z
1	6	0	-2.133828	2.699193	0.476493
2	6	0	4.281313	3.327150	-1.775445
3	6	0	4.052916	1.983553	-1.469613
4	6	0	3.312579	1.629651	-0.332604
5	6	0	2.797258	2.643853	0.486371
6	6	0	3.023995	3.986178	0.179264
7	6	0	3.767894	4.327661	-0.949995
8	6	0	3.071726	0.209473	0.001517
9	6	0	4.128978	-0.520254	0.393602
10	7	0	4.007803	-1.852365	0.744439
11	6	0	2.785624	-2.518724	0.736125
12	6	0	1.593762	-1.731508	0.276092
13	6	0	1.769181	-0.434759	-0.043438
14	6	0	5.202406	-2.558971	1.182458

15	6	0	0.288574	-2.494658	0.258295
16	8	0	0.735838	0.388184	-0.445127
17	6	0	0.206248	-3.451786	-0.936631
18	6	0	-1.145566	-4.159919	-0.966640
19	6	0	-2.299808	-3.148465	-0.917534
20	6	0	-2.090366	-2.180186	0.267043
21	8	0	-0.800090	-1.557131	0.167963
22	6	0	-3.108330	-1.048285	0.327579
23	6	0	-3.639985	-3.879895	-0.852423
24	6	0	-4.059308	-1.130957	1.491010
25	6	0	-3.096942	-0.051349	-0.585011
26	6	0	-3.953485	1.192313	-0.638255
27	6	0	-4.879182	1.111373	-1.855436
28	6	0	-3.079126	2.463952	-0.723602
29	6	0	-2.848237	2.816491	1.837471
30	6	0	-3.897712	3.914714	1.929485
31	6	0	-1.252106	3.923287	0.205513
32	8	0	2.646986	-3.691578	1.081815
33	1	0	-1.453479	1.841277	0.551584
34	1	0	4.851538	3.593139	-2.660600
35	1	0	4.446631	1.213764	-2.129418
36	1	0	2.204910	2.388818	1.361955
37	1	0	2.614573	4.764212	0.816790
38	1	0	3.940602	5.373352	-1.191260
39	1	0	5.126219	-0.093175	0.461304
40	1	0	5.986625	-2.445472	0.427236
41	1	0	5.540694	-2.127111	2.129751
42	1	0	5.014485	-3.625897	1.330622
43	1	0	0.177759	-3.039869	1.205001
44	1	0	-0.098824	-0.109398	-0.302079

45	1	0	0.321260	-2.887610	-1.871646
46	1	0	1.006873	-4.199046	-0.908119
47	1	0	-1.217733	-4.772611	-1.872982
48	1	0	-1.212047	-4.845606	-0.111615
49	1	0	-2.264806	-2.578819	-1.855792
50	1	0	-2.116469	-2.735772	1.214195
51	1	0	-3.733254	-4.592583	-1.677836
52	1	0	-3.743328	-4.435757	0.086555
53	1	0	-4.478643	-3.181125	-0.928128
54	1	0	-3.514948	-0.989876	2.430266
55	1	0	-4.861533	-0.392361	1.452834
56	1	0	-4.539650	-2.114837	1.516704
57	1	0	-2.358898	-0.110470	-1.387902
58	1	0	-4.591202	1.275217	0.244772
59	1	0	-5.522120	0.226306	-1.800545
60	1	0	-5.529376	1.991752	-1.906711
61	1	0	-4.312159	1.058500	-2.791656
62	1	0	-2.471275	2.415106	-1.639356
63	1	0	-3.730172	3.338345	-0.850913
64	1	0	-3.320075	1.860475	2.088546
65	1	0	-2.096179	2.989907	2.617627
66	1	0	-3.468320	4.900850	1.732632
67	1	0	-4.719498	3.746128	1.227430
68	1	0	-4.325503	3.936022	2.937071
69	1	0	-0.638313	3.764479	-0.688289
70	1	0	-1.848831	4.826093	0.040321
71	1	0	-0.572875	4.109653	1.043240

Table S3 Gibbs free energies and Boltzmann-population of low-energy conformers of 7*S*, 11*R*, 13*E*, 14*R*, 16*S* -**2**.

Conformers of Compound	In MeOH	
	ΔG[a]	P(%)[b]
**2a**	0.03	35.42%
**2b**	0.00	37.45%
**2c**	0.70	11.45%
**2d**	0.66	12.27%
**2e**	2.01	1.25%
**2f**	2.10	1.08%
**2g**	2.10	1.08%

Table S4 Cartesian coordinates for the low-energy optimized conformers of 7*S*, 11*R*, 13*E*, 14*R*, 16*S* **-2** at B3LYP/6-31+G (d, p) level of theory in MeOH.

2a

Standard orientation:

Center Number	Atomic Number	Atomic Type	Coordinates (Angstroms) X	Y	Z
1	6	0	-4.984870	3.248073	-0.626945
2	6	0	-4.341056	2.025107	-0.831953
3	6	0	-4.490613	0.977666	0.089301
4	6	0	-5.289565	1.175454	1.224900
5	6	0	-5.935531	2.396739	1.428664
6	6	0	-5.778843	3.421307	0.501845
7	6	0	-3.815599	-0.316127	-0.140768
8	6	0	-4.592144	-1.364222	-0.463103
9	7	0	-4.063897	-2.617393	-0.707150
10	6	0	-2.697710	-2.875928	-0.647620
11	6	0	-1.810329	-1.726115	-0.266916
12	6	0	-2.378054	-0.522109	-0.057006

13	6	0	-4.979165	-3.690106	-1.069771
14	6	0	-0.337596	-2.058270	-0.188569
15	8	0	-1.652794	0.607691	0.265406
16	6	0	-0.008105	-2.875909	1.075336
17	6	0	1.475294	-3.019781	1.249180
18	6	0	2.335657	-2.210145	0.604507
19	6	0	1.843755	-1.072628	-0.281136
20	8	0	0.429546	-0.838474	-0.142453
21	6	0	2.519869	0.257619	0.027573
22	6	0	3.821654	-2.424563	0.689793
23	6	0	2.268370	0.814082	1.408644
24	6	0	3.278624	0.865805	-0.910663
25	6	0	4.061330	2.154416	-0.791423
26	6	0	3.400059	3.226366	-1.665236
27	6	0	5.534686	1.964377	-1.218524
28	6	0	6.335432	0.899497	-0.430062
29	6	0	6.415335	1.237063	1.072141
30	6	0	7.183618	0.210191	1.894404
31	6	0	7.730960	0.769144	-1.055743
32	8	0	-6.424675	4.597444	0.741478
33	8	0	-2.201275	-3.974735	-0.889979
34	1	0	-4.851373	4.043723	-1.351341
35	1	0	-3.712276	1.891071	-1.709136
36	1	0	-5.406872	0.383530	1.960476
37	1	0	-6.552219	2.550037	2.309558
38	1	0	-5.671614	-1.269951	-0.552480
39	1	0	-4.466331	-4.650505	-1.163148
40	1	0	-5.751375	-3.779943	-0.299715
41	1	0	-5.444949	-3.446274	-2.029917
42	1	0	-0.044791	-2.599896	-1.097964

43	1	0	-0.702319	0.367308	0.206029
44	1	0	-0.407081	-2.385897	1.972043
45	1	0	-0.457669	-3.872852	1.014758
46	1	0	1.828533	-3.822115	1.892008
47	1	0	2.022645	-1.357084	-1.326468
48	1	0	4.071347	-3.344852	1.228862
49	1	0	4.251636	-2.506581	-0.314156
50	1	0	4.309426	-1.596358	1.212490
51	1	0	1.200522	0.800601	1.646002
52	1	0	2.793814	0.219165	2.163204
53	1	0	2.589866	1.851498	1.522037
54	1	0	3.369364	0.384113	-1.885076
55	1	0	4.054511	2.532468	0.233301
56	1	0	3.392656	2.937232	-2.721982
57	1	0	2.363048	3.399053	-1.354801
58	1	0	3.934924	4.177730	-1.581328
59	1	0	5.560087	1.700836	-2.285413
60	1	0	6.051070	2.930025	-1.133992
61	1	0	5.838188	-0.071662	-0.542706
62	1	0	6.875763	2.223053	1.211593
63	1	0	5.406202	1.297168	1.490536
64	1	0	6.792191	-0.798740	1.730838
65	1	0	8.250365	0.216085	1.650705
66	1	0	7.090794	0.440957	2.961441
67	1	0	7.658179	0.586889	-2.133309
68	1	0	8.319525	1.680755	-0.905676
69	1	0	8.283997	-0.071705	-0.626403
70	1	0	-6.191693	5.225449	0.038460

2b

Standard orientation:

Center Number	Atomic Number	Atomic Type	Coordinates (Angstroms) X	Y	Z
1	6	0	-5.739236	2.871343	-0.792752
2	6	0	-5.089559	1.653775	-1.003377
3	6	0	-4.490914	0.976310	0.068688
4	6	0	-4.543227	1.542156	1.350532
5	6	0	-5.191364	2.762293	1.561341
6	6	0	-5.785230	3.414972	0.485557
7	6	0	-3.813362	-0.320605	-0.142238
8	6	0	-4.590747	-1.378081	-0.427296
9	7	0	-4.061660	-2.637960	-0.642167
10	6	0	-2.694563	-2.891198	-0.586550
11	6	0	-1.807511	-1.732494	-0.235008
12	6	0	-2.375956	-0.525186	-0.049484
13	6	0	-4.977139	-3.721056	-0.969207
14	6	0	-0.333620	-2.060100	-0.153651
15	8	0	-1.652125	0.612362	0.244821
16	6	0	0.000745	-2.851002	1.124928
17	6	0	1.485435	-2.988826	1.297911
18	6	0	2.342372	-2.191630	0.633312
19	6	0	1.845604	-1.073862	-0.274778
20	8	0	0.432019	-0.838285	-0.135355
21	6	0	2.520554	0.263824	0.003631
22	6	0	3.829561	-2.402082	0.717865
23	6	0	2.272421	0.849260	1.372127
24	6	0	3.275814	0.853003	-0.950034
25	6	0	4.056465	2.144988	-0.863039

26	6	0	3.390789	3.195606	-1.758251
27	6	0	5.527684	1.948025	-1.289708
28	6	0	6.333684	0.902712	-0.478816
29	6	0	6.416752	1.273780	1.015350
30	6	0	7.189031	0.267053	1.857547
31	6	0	7.727029	0.760487	-1.105487
32	8	0	-6.428142	4.607179	0.645893
33	8	0	-2.197855	-3.994754	-0.805530
34	1	0	-6.200690	3.396099	-1.624551
35	1	0	-5.045663	1.240946	-2.008816
36	1	0	-4.069355	1.036007	2.188299
37	1	0	-5.216340	3.182874	2.561220
38	1	0	-5.671921	-1.290852	-0.501623
39	1	0	-4.462368	-4.681588	-1.048002
40	1	0	-5.738913	-3.796592	-0.186491
41	1	0	-5.458144	-3.500559	-1.927707
42	1	0	-0.043797	-2.620819	-1.052863
43	1	0	-0.701673	0.378592	0.164610
44	1	0	-0.395784	-2.343154	2.013207
45	1	0	-0.447426	-3.849365	1.087902
46	1	0	1.841938	-3.776169	1.955932
47	1	0	2.020790	-1.380336	-1.313929
48	1	0	4.081290	-3.309879	1.276050
49	1	0	4.254790	-2.505443	-0.285774
50	1	0	4.316637	-1.561991	1.220717
51	1	0	1.204711	0.842916	1.611151
52	1	0	2.797839	0.270257	2.138414
53	1	0	2.596151	1.888279	1.463583
54	1	0	3.363423	0.349980	-1.915025
55	1	0	4.051689	2.546034	0.153703

56	1	0	3.380092	2.882095	-2.807936
57	1	0	2.354026	3.373170	-1.448873
58	1	0	3.923977	4.149501	-1.697149
59	1	0	5.551935	1.660656	-2.349845
60	1	0	6.043546	2.916512	-1.227656
61	1	0	5.838686	-0.071597	-0.568810
62	1	0	6.876124	2.262926	1.130871
63	1	0	5.407688	1.341260	1.434378
64	1	0	6.798711	-0.746529	1.717761
65	1	0	8.255277	0.268575	1.611134
66	1	0	7.098752	0.520630	2.919800
67	1	0	7.651848	0.554247	-2.178829
68	1	0	8.314552	1.676252	-0.977222
69	1	0	8.282791	-0.069504	-0.659411
70	1	0	-6.343805	4.879483	1.573627

2c

Standard orientation:

Center	Atomic	Atomic	Coordinates (Angstroms)		
Number	Number	Type	X	Y	Z
1	6	0	-4.836761	3.355184	-0.741496
2	6	0	-4.244442	2.099757	-0.905939
3	6	0	-4.442491	1.089653	0.046249
4	6	0	-5.237512	1.355862	1.170441
5	6	0	-5.830197	2.609061	1.333935
6	6	0	-5.625975	3.597170	0.377261
7	6	0	-3.821917	-0.238076	-0.141669
8	6	0	-4.642168	-1.260871	-0.436331

9	7	0	-4.167214	-2.543472	-0.639091
10	6	0	-2.813663	-2.857380	-0.564979
11	6	0	-1.879724	-1.736244	-0.213274
12	6	0	-2.396631	-0.503803	-0.043010
13	6	0	-5.125733	-3.586829	-0.975289
14	6	0	-0.422403	-2.128195	-0.116178
15	8	0	-1.624318	0.603057	0.248779
16	6	0	-0.134051	-2.920397	1.172175
17	6	0	1.341576	-3.123884	1.358172
18	6	0	2.238850	-2.371946	0.694387
19	6	0	1.799508	-1.240844	-0.226660
20	8	0	0.396314	-0.941816	-0.103112
21	6	0	2.532791	0.067642	0.048270
22	6	0	3.713635	-2.648524	0.791988
23	6	0	2.292518	0.677616	1.407796
24	6	0	3.327985	0.609671	-0.900000
25	6	0	4.169381	1.863746	-0.812249
26	6	0	3.556986	2.942657	-1.710213
27	6	0	5.630301	1.594349	-1.234546
28	6	0	6.384081	0.540080	-0.392331
29	6	0	6.433614	0.844459	1.117542
30	6	0	7.067349	2.177282	1.493030
31	6	0	7.793161	0.341347	-0.963799
32	8	0	-6.223234	4.807216	0.577510
33	8	0	-2.363634	-3.983367	-0.770749
34	1	0	-4.665338	4.122993	-1.489343
35	1	0	-3.617895	1.913547	-1.775284
36	1	0	-5.390523	0.592325	1.929593
37	1	0	-6.443288	2.815308	2.205816
38	1	0	-5.717001	-1.124020	-0.534520

39	1	0	-5.905292	-3.619223	-0.206809
40	1	0	-5.576804	-3.352258	-1.944625
41	1	0	-4.654758	-4.571039	-1.036180
42	1	0	-0.149403	-2.709351	-1.008705
43	1	0	-0.685085	0.321609	0.201463
44	1	0	-0.514109	-2.387401	2.052735
45	1	0	-0.626738	-3.898619	1.140489
46	1	0	1.656820	-3.921943	2.024402
47	1	0	1.970168	-1.562982	-1.262233
48	1	0	3.920840	-3.562273	1.359604
49	1	0	4.141732	-2.781347	-0.207158
50	1	0	4.236850	-1.827227	1.290388
51	1	0	2.674272	1.696401	1.497873
52	1	0	1.221801	0.735423	1.625647
53	1	0	2.768123	0.074973	2.188576
54	1	0	3.405088	0.093301	-1.858348
55	1	0	4.182389	2.262519	0.205122
56	1	0	3.537182	2.630140	-2.761046
57	1	0	2.529030	3.168841	-1.406860
58	1	0	4.133970	3.872062	-1.647078
59	1	0	5.642269	1.269811	-2.285254
60	1	0	6.186474	2.540781	-1.212859
61	1	0	5.866594	-0.421512	-0.506233
62	1	0	5.421300	0.808029	1.534266
63	1	0	6.989714	0.043204	1.622010
64	1	0	6.510315	3.020044	1.074889
65	1	0	7.070620	2.293265	2.581881
66	1	0	8.103968	2.240945	1.150539
67	1	0	8.367749	1.272527	-0.961918
68	1	0	8.348048	-0.404785	-0.384859

69	1	0	7.741365	-0.014209	-1.998106
70	1	0	-5.959363	5.402468	-0.143363

2d

Standard orientation:

Center Number	Atomic Number	Atomic Type	Coordinates (Angstroms) X	Y	Z
1	6	0	-5.604820	3.006567	-0.898379
2	6	0	-5.007247	1.757038	-1.069596
3	6	0	-4.442298	1.086457	0.025439
4	6	0	-4.477331	1.691561	1.289948
5	6	0	-5.073571	2.943454	1.460368
6	6	0	-5.634601	3.589095	0.363173
7	6	0	-3.819860	-0.243457	-0.144313
8	6	0	-4.640550	-1.275719	-0.403318
9	7	0	-4.164543	-2.561497	-0.579308
10	6	0	-2.809759	-2.871097	-0.507707
11	6	0	-1.876297	-1.740779	-0.183236
12	6	0	-2.394268	-0.506400	-0.036677
13	6	0	-5.124642	-3.613677	-0.879734
14	6	0	-0.418715	-2.129241	-0.083144
15	8	0	-1.622973	0.607681	0.229243
16	6	0	-0.124217	-2.897102	1.219463
17	6	0	1.352688	-3.093497	1.404573
18	6	0	2.245718	-2.353219	0.722687
19	6	0	1.801733	-1.240197	-0.217882
20	8	0	0.398907	-0.941126	-0.094891
21	6	0	2.533735	0.073586	0.027468

22	6	0	3.722229	-2.625452	0.820221
23	6	0	2.296728	0.710258	1.376381
24	6	0	3.325376	0.598290	-0.933986
25	6	0	4.164134	1.855331	-0.874064
26	6	0	3.546117	2.915015	-1.792254
27	6	0	5.624425	1.580377	-1.296221
28	6	0	6.382619	0.544021	-0.435665
29	6	0	6.437565	0.879429	1.068004
30	6	0	7.069143	2.220389	1.414142
31	6	0	7.790678	0.337033	-1.007199
32	8	0	-6.226235	4.811778	0.484772
33	8	0	-2.359971	-4.000694	-0.691005
34	1	0	-6.039375	3.526501	-1.747623
35	1	0	-4.976285	1.313493	-2.062461
36	1	0	-4.029759	1.190709	2.144796
37	1	0	-5.086121	3.393470	2.448027
38	1	0	-5.716998	-1.144003	-0.486515
39	1	0	-4.650406	-4.597938	-0.927328
40	1	0	-5.892200	-3.634350	-0.099713
41	1	0	-5.589348	-3.401818	-1.847429
42	1	0	-0.147711	-2.727238	-0.964214
43	1	0	-0.683296	0.331308	0.161433
44	1	0	-0.503054	-2.346604	2.090392
45	1	0	-0.615041	-3.875393	1.208332
46	1	0	1.671761	-3.877531	2.085338
47	1	0	1.969090	-1.582864	-1.247975
48	1	0	3.932501	-3.527720	1.404893
49	1	0	4.146250	-2.776390	-0.177907
50	1	0	4.245163	-1.793760	1.301164
51	1	0	1.226667	0.773219	1.594417

52	1	0	2.773904	0.122730	2.166732
53	1	0	2.680562	1.730486	1.445791
54	1	0	3.399524	0.063705	-1.881151
55	1	0	4.179804	2.275361	0.134628
56	1	0	3.523449	2.581332	-2.835140
57	1	0	2.518983	3.145071	-1.488624
58	1	0	4.121432	3.846109	-1.749585
59	1	0	5.633678	1.235116	-2.340106
60	1	0	6.178777	2.527771	-1.295514
61	1	0	5.867270	-0.419835	-0.527859
62	1	0	5.426229	0.849877	1.488936
63	1	0	6.996420	0.089085	1.586701
64	1	0	8.105064	2.279567	1.067026
65	1	0	6.508706	3.053934	0.980791
66	1	0	7.076475	2.358202	2.500446
67	1	0	7.735559	-0.039229	-2.034922
68	1	0	8.364274	1.268689	-1.025985
69	1	0	8.348783	-0.397122	-0.415946
70	1	0	-6.135025	5.107035	1.405398

2e

Standard orientation:

Center Number	Atomic Number	Atomic Type	Coordinates (Angstroms) X	Y	Z
1	6	0	-5.065800	-1.647449	1.004429
2	6	0	-4.334874	-0.458275	1.025056
3	6	0	-3.462126	-0.143671	-0.026950
4	6	0	-3.328893	-1.044581	-1.093889

5	6	0	-4.056604	-2.237377	-1.113776
6	6	0	-4.921390	-2.525823	-0.062927
7	6	0	-2.679097	1.111684	-0.011460
8	6	0	-3.356040	2.268484	-0.100679
9	7	0	-2.714745	3.493575	-0.090554
10	6	0	-1.331845	3.612428	0.021296
11	6	0	-0.550045	2.333902	0.083520
12	6	0	-1.228582	1.169581	0.081271
13	6	0	-3.530213	4.696870	-0.166623
14	6	0	0.950439	2.499869	0.169194
15	8	0	-0.612607	-0.062096	0.163470
16	6	0	1.549581	2.929008	-1.182341
17	6	0	3.048833	2.861201	-1.157147
18	6	0	3.699854	2.148421	-0.219671
19	6	0	2.947408	1.334848	0.824303
20	8	0	1.539876	1.240895	0.546684
21	6	0	3.413738	-0.112288	0.934500
22	6	0	5.201328	2.162189	-0.129165
23	6	0	3.774481	-0.529972	2.334245
24	6	0	3.454852	-0.926343	-0.144989
25	6	0	3.827464	-2.389252	-0.204905
26	6	0	5.218999	-2.526334	-0.832093
27	6	0	2.801548	-3.207984	-1.023052
28	6	0	1.335108	-3.143793	-0.529954
29	6	0	1.185764	-3.675049	0.908293
30	6	0	-0.241341	-3.628231	1.440081
31	6	0	0.447077	-3.913735	-1.516471
32	8	0	-5.651295	-3.678329	-0.040935
33	8	0	-0.741257	4.690355	0.066569
34	1	0	-5.738514	-1.891536	1.821685

35	1	0	-4.440483	0.214293	1.873238
36	1	0	-2.642148	-0.825547	-1.908227
37	1	0	-3.930921	-2.920557	-1.947044
38	1	0	-4.439962	2.292258	-0.183573
39	1	0	-2.919830	5.601185	-0.230083
40	1	0	-4.167631	4.643462	-1.056467
41	1	0	-4.156416	4.757002	0.729454
42	1	0	1.189268	3.226844	0.957580
43	1	0	0.343617	0.097872	0.322076
44	1	0	1.198047	2.275275	-1.990195
45	1	0	1.242820	3.950847	-1.429537
46	1	0	3.588556	3.430267	-1.908746
47	1	0	3.058369	1.840706	1.791401
48	1	0	5.645419	2.876438	-0.830390
49	1	0	5.515982	2.452987	0.878831
50	1	0	5.618808	1.176735	-0.355305
51	1	0	4.534380	0.144953	2.741908
52	1	0	2.890511	-0.485110	2.978038
53	1	0	4.183646	-1.539644	2.398762
54	1	0	3.168074	-0.499537	-1.107769
55	1	0	3.881819	-2.825598	0.796588
56	1	0	5.241414	-2.132036	-1.853987
57	1	0	5.968377	-1.983709	-0.245120
58	1	0	5.524548	-3.577750	-0.870431
59	1	0	2.832904	-2.867694	-2.067043
60	1	0	3.123929	-4.258293	-1.037193
61	1	0	1.006254	-2.098886	-0.551977
62	1	0	1.554647	-4.705725	0.966623
63	1	0	1.805574	-3.077959	1.586916
64	1	0	-0.664363	-2.623680	1.348531

65	1	0	-0.891832	-4.331066	0.912371
66	1	0	-0.253507	-3.903991	2.500337
67	1	0	0.607947	-3.564466	-2.541325
68	1	0	0.660937	-4.987227	-1.486130
69	1	0	-0.614817	-3.768332	-1.294253
70	1	0	-5.417696	-4.204371	-0.822561

2f

Standard orientation:

Center Number	Atomic Number	Atomic Type	Coordinates (Angstroms) X	Y	Z
1	6	0	-4.191882	-2.249128	0.973645
2	6	0	-3.465164	-1.057102	1.031692
3	6	0	-3.471288	-0.163100	-0.049484
4	6	0	-4.214766	-0.484767	-1.195174
5	6	0	-4.944400	-1.673219	-1.252371
6	6	0	-4.927172	-2.544454	-0.170020
7	6	0	-2.694539	1.093208	0.020469
8	6	0	-3.381794	2.247105	0.048449
9	7	0	-2.747959	3.474567	0.118862
10	6	0	-1.362331	3.596329	0.174128
11	6	0	-0.571581	2.322731	0.129227
12	6	0	-1.242342	1.156203	0.074734
13	6	0	-3.574351	4.671994	0.159446
14	6	0	0.929349	2.497411	0.175143
15	8	0	-0.615760	-0.072453	0.054161
16	6	0	1.482485	2.988411	-1.175232
17	6	0	2.982050	2.927244	-1.201062

18	6	0	3.668745	2.188278	-0.308941
19	6	0	2.954316	1.340700	0.733526
20	8	0	1.542127	1.229588	0.481236
21	6	0	3.457616	-0.096619	0.808632
22	6	0	5.171515	2.207419	-0.270899
23	6	0	3.944716	-0.500702	2.173643
24	6	0	3.431709	-0.911147	-0.270145
25	6	0	3.837902	-2.364561	-0.360425
26	6	0	5.158922	-2.468751	-1.128198
27	6	0	2.756563	-3.220122	-1.059014
28	6	0	1.343699	-3.176696	-0.427216
29	6	0	1.343833	-3.678741	1.029829
30	6	0	-0.017726	-3.595873	1.707049
31	6	0	0.381298	-3.984957	-1.304895
32	8	0	-5.650403	-3.696160	-0.270341
33	8	0	-0.776740	4.675009	0.256437
34	1	0	-4.165862	-2.926930	1.819894
35	1	0	-2.879322	-0.831324	1.919923
36	1	0	-4.220117	0.182517	-2.053345
37	1	0	-5.516434	-1.922624	-2.140943
38	1	0	-4.468745	2.266925	0.022382
39	1	0	-2.971809	5.584483	0.158759
40	1	0	-4.230212	4.685816	-0.716497
41	1	0	-4.180399	4.653283	1.070695
42	1	0	1.186337	3.192599	0.986153
43	1	0	0.346123	0.086810	0.179304
44	1	0	1.107382	2.367299	-1.998193
45	1	0	1.162729	4.018059	-1.366436
46	1	0	3.493278	3.521456	-1.952893
47	1	0	3.074312	1.828871	1.709772

48	1	0	5.587494	2.947216	-0.964024
49	1	0	5.521904	2.465924	0.733177
50	1	0	5.584908	1.232475	-0.546044
51	1	0	4.715406	0.196892	2.518007
52	1	0	3.115289	-0.478961	2.888577
53	1	0	4.386694	-1.497255	2.206162
54	1	0	3.057342	-0.491780	-1.206945
55	1	0	4.010140	-2.788791	0.631678
56	1	0	5.063431	-2.082828	-2.150057
57	1	0	5.949602	-1.899346	-0.626856
58	1	0	5.488892	-3.511129	-1.191890
59	1	0	2.677335	-2.899603	-2.107704
60	1	0	3.099702	-4.263662	-1.086772
61	1	0	0.993222	-2.139143	-0.437092
62	1	0	1.701790	-4.714794	1.068459
63	1	0	2.039345	-3.080894	1.627442
64	1	0	-0.426596	-2.582453	1.644241
65	1	0	-0.734460	-4.290498	1.260020
66	1	0	0.074866	-3.857145	2.766561
67	1	0	0.432107	-3.653288	-2.347339
68	1	0	0.621814	-5.053673	-1.277657
69	1	0	-0.656784	-3.858470	-0.981363
70	1	0	-5.511077	-4.218435	0.537658

2g

Standard orientation:

Center	Atomic	Atomic	Coordinates (Angstroms)		
Number	Number	Type	X	Y	Z

1	6	0	-4.074458	-2.398948	0.626318
2	6	0	-3.363557	-1.208384	0.797963
3	6	0	-3.447188	-0.182449	-0.155256
4	6	0	-4.250313	-0.369486	-1.289663
5	6	0	-4.963706	-1.558232	-1.460610
6	6	0	-4.870356	-2.562049	-0.503105
7	6	0	-2.687565	1.072490	0.035446
8	6	0	-3.393083	2.199057	0.238469
9	7	0	-2.777148	3.422002	0.436684
10	6	0	-1.392661	3.567602	0.448366
11	6	0	-0.583819	2.326569	0.214622
12	6	0	-1.236645	1.161439	0.037846
13	6	0	-3.621157	4.586689	0.659306
14	6	0	0.914523	2.525164	0.223799
15	8	0	-0.589663	-0.040699	-0.168604
16	6	0	1.402437	3.166425	-1.087182
17	6	0	2.898379	3.112837	-1.198898
18	6	0	3.632487	2.292855	-0.423565
19	6	0	2.977221	1.346674	0.573032
20	8	0	1.554232	1.243712	0.370089
21	6	0	3.504219	-0.081913	0.486871
22	6	0	5.134524	2.311021	-0.478401
23	6	0	4.310215	-0.511623	1.681152
24	6	0	3.214121	-0.869921	-0.573068
25	6	0	3.573329	-2.319751	-0.801623
26	6	0	4.488712	-2.420539	-2.026054
27	6	0	2.305276	-3.177919	-1.019760
28	6	0	1.303719	-3.199311	0.161000
29	6	0	1.903656	-3.880980	1.405295
30	6	0	0.980483	-3.864580	2.616920

31	6	0	0.012045	-3.891013	-0.294441
32	8	0	-5.580235	-3.706881	-0.711472
33	8	0	-0.821327	4.640818	0.635389
34	1	0	-3.988415	-3.179023	1.374434
35	1	0	-2.730494	-1.086093	1.674545
36	1	0	-4.316880	0.402021	-2.051965
37	1	0	-5.583738	-1.703017	-2.340961
38	1	0	-4.479601	2.197731	0.262476
39	1	0	-4.306019	4.700335	-0.187349
40	1	0	-4.196152	4.436981	1.579210
41	1	0	-3.033884	5.504226	0.758661
42	1	0	1.195561	3.136331	1.093182
43	1	0	0.370787	0.120690	-0.040900
44	1	0	0.984723	2.642207	-1.956002
45	1	0	1.075258	4.210054	-1.144527
46	1	0	3.368126	3.777231	-1.918452
47	1	0	3.129930	1.740535	1.586467
48	1	0	5.510428	3.105375	-1.132206
49	1	0	5.549287	2.485124	0.519943
50	1	0	5.523890	1.361615	-0.860232
51	1	0	4.770502	-1.494964	1.569311
52	1	0	5.125360	0.197447	1.861883
53	1	0	3.674636	-0.540204	2.572095
54	1	0	2.619253	-0.429483	-1.375472
55	1	0	4.130305	-2.732831	0.042129
56	1	0	3.994148	-2.053621	-2.932256
57	1	0	5.403579	-1.834651	-1.880519
58	1	0	4.785308	-3.460897	-2.202511
59	1	0	1.786278	-2.810757	-1.916373
60	1	0	2.608291	-4.208766	-1.245383

61	1	0	1.041601	-2.168689	0.424298
62	1	0	2.172197	-4.917814	1.173952
63	1	0	2.828691	-3.373206	1.696139
64	1	0	0.099432	-4.494925	2.461485
65	1	0	1.508200	-4.250683	3.495143
66	1	0	0.646432	-2.846273	2.843246
67	1	0	0.183493	-4.950786	-0.505941
68	1	0	-0.771951	-3.814288	0.465740
69	1	0	-0.380366	-3.422484	-1.203957
70	1	0	-5.387244	-4.324401	0.011982

Table S5 Gibbs free energies and Boltzmann-population of low-energy conformers of 2*R*, 3*R* -**7**.

Conformers of Compound	In MeOH	
	ΔG[a]	P(%)[b]
**7a**	0.00	28.61%
**7b**	0.29	17.45%
**7c**	0.77	7.81%
**7d**	1.04	4.91%
**7e**	0.99	5.32%
**7f**	0.27	17.87%
**7g**	0.27	18.02%

Table S6 Cartesian coordinates for the low-energy optimized conformers of 2*R*, 3*R* -**7** at B3LYP/6-31+G (d, p) level of theory in MeOH.

7a

Standard orientation:

Center	Atomic	Atomic	Coordinates (Angstroms)		
Number	Number	Type	X	Y	Z

1	6	0	-0.385768	0.156118	1.195915
2	6	0	-1.406500	-0.893125	0.673242
3	6	0	-2.368687	-0.082995	-0.165954
4	6	0	-2.010895	1.190702	-0.370922
5	6	0	-0.743841	1.383059	0.358326
6	8	0	-0.095407	2.419866	0.312579
7	6	0	-2.672862	2.279298	-1.120903
8	8	0	-3.572306	-0.577517	-0.614931
9	6	0	-3.608045	-1.971549	-0.901889
10	8	0	0.994360	-0.227794	1.168725
11	6	0	-0.695117	0.515999	2.656556
12	6	0	1.588729	-0.357600	-0.047764
13	6	0	3.074342	-0.616231	0.115727
14	8	0	0.994785	-0.318228	-1.118037
15	6	0	3.309514	-2.122294	0.043187
16	6	0	3.888161	0.123000	-0.955534
17	6	0	3.819140	1.636439	-0.800257
18	8	0	-0.825768	-1.928551	-0.113692
19	1	0	-1.936351	-1.371710	1.504048
20	1	0	-2.023664	2.638867	-1.926010
21	1	0	-2.898144	3.120416	-0.456647
22	1	0	-3.612290	1.948152	-1.572099
23	1	0	-4.599551	-2.204593	-1.300505
24	1	0	-2.870449	-2.232481	-1.667796
25	1	0	-3.466046	-2.567417	0.004610
26	1	0	-0.023090	1.305971	3.011181
27	1	0	-0.533514	-0.349094	3.309114
28	1	0	-1.724559	0.866852	2.790604
29	1	0	3.388955	-0.254545	1.102643

30	1	0	3.010791	-2.524813	-0.931642
31	1	0	2.725144	-2.645615	0.807267
32	1	0	4.366179	-2.360691	0.201333
33	1	0	4.939357	-0.183679	-0.889919
34	1	0	3.539654	-0.148597	-1.959720
35	1	0	4.177405	1.945520	0.187265
36	1	0	4.448257	2.120500	-1.553594
37	1	0	2.797792	2.007734	-0.928321
38	1	0	-0.400215	-1.497913	-0.887367

7b

Standard orientation:

Center Number	Atomic Number	Atomic Type	Coordinates (Angstroms) X	Y	Z
1	6	0	0.251660	-0.506428	0.958734
2	6	0	1.095846	0.788541	0.795883
3	6	0	2.324500	0.331912	0.041604
4	6	0	2.278866	-0.925483	-0.412356
5	6	0	0.983266	-1.474062	0.029880
6	8	0	0.582551	-2.585095	-0.290467
7	6	0	3.262807	-1.721610	-1.176742
8	8	0	3.452089	1.110667	-0.094821
9	6	0	3.236573	2.516955	-0.165400
10	8	0	-1.157443	-0.384958	0.733621
11	6	0	0.404602	-1.064544	2.381640
12	6	0	-1.579096	-0.147140	-0.535137
13	6	0	-3.089266	-0.224771	-0.649060
14	8	0	-0.837056	0.124354	-1.472606

15	6	0	-3.560777	-1.633482	-0.293659
16	6	0	-3.784655	0.830930	0.220741
17	6	0	-3.481503	2.254133	-0.230004
18	8	0	0.438860	1.825269	0.073274
19	1	0	1.377517	1.197445	1.772287
20	1	0	2.837196	-2.039873	-2.132669
21	1	0	3.554738	-2.614551	-0.614186
22	1	0	4.171412	-1.147969	-1.389458
23	1	0	4.206153	2.994140	-0.332959
24	1	0	2.588327	2.770618	-1.010023
25	1	0	2.833121	2.904200	0.774623
26	1	0	-0.131501	-2.015390	2.484535
27	1	0	-0.033081	-0.376612	3.113445
28	1	0	1.451895	-1.238826	2.650692
29	1	0	-3.341677	-0.043236	-1.701117
30	1	0	-3.384112	-1.864219	0.763555
31	1	0	-3.028106	-2.384086	-0.887879
32	1	0	-4.631747	-1.745451	-0.488729
33	1	0	-4.869969	0.679617	0.176492
34	1	0	-3.492031	0.721179	1.271804
35	1	0	-3.770278	2.404639	-1.274724
36	1	0	-4.039911	2.969299	0.382401
37	1	0	-2.417558	2.487945	-0.129628
38	1	0	0.230640	1.469522	-0.817214

7c

Standard orientation:

Center Number	Atomic Number	Atomic Type	Coordinates (Angstroms) X	Y	Z

1	6	0	-0.353861	0.303619	1.129275
2	6	0	-1.477457	-0.731430	0.846759
3	6	0	-2.398782	-0.018990	-0.117871
4	6	0	-1.943940	1.153001	-0.576731
5	6	0	-0.639300	1.370917	0.074620
6	8	0	0.090102	2.318146	-0.186551
7	6	0	-2.539757	2.129386	-1.513706
8	8	0	-3.655469	-0.481720	-0.436090
9	6	0	-3.818015	-1.897480	-0.440959
10	8	0	0.988311	-0.198336	1.150814
11	6	0	-0.577500	0.967793	2.496218
12	6	0	1.526434	-0.617154	-0.026812
13	6	0	2.999067	-0.940591	0.152899
14	8	0	0.894527	-0.737576	-1.069763
15	6	0	3.517385	-1.785695	-1.008935
16	6	0	3.820717	0.338583	0.375767
17	6	0	3.862658	1.286052	-0.817184
18	8	0	-1.014950	-1.948814	0.267190
19	1	0	-2.013938	-0.989533	1.765794
20	1	0	-1.893398	2.266036	-2.386938
21	1	0	-2.669752	3.100509	-1.025876
22	1	0	-3.521508	1.801274	-1.872199
23	1	0	-4.840208	-2.113900	-0.766159
24	1	0	-3.133956	-2.366379	-1.153878
25	1	0	-3.693723	-2.314421	0.563160
26	1	0	0.172135	1.748016	2.674280
27	1	0	-0.464348	0.235912	3.303621
28	1	0	-1.567616	1.428136	2.579945
29	1	0	3.076202	-1.562358	1.055474

30	1	0	3.388167	-1.283699	-1.973833
31	1	0	2.969887	-2.733746	-1.065777
32	1	0	4.578904	-2.017568	-0.880350
33	1	0	3.418440	0.887513	1.236129
34	1	0	4.848736	0.061972	0.637623
35	1	0	2.857452	1.590444	-1.122934
36	1	0	4.420827	2.189946	-0.554582
37	1	0	4.363686	0.827586	-1.675787
38	1	0	-0.582044	-1.717392	-0.582546

7d

Standard orientation:

Center Number	Atomic Number	Atomic Type	Coordinates (Angstroms) X	Y	Z
1	6	0	-0.324515	-0.786957	-0.572323
2	6	0	-1.016207	0.591373	-0.742458
3	6	0	-2.389519	0.393033	-0.152713
4	6	0	-2.563757	-0.756174	0.505164
5	6	0	-1.282425	-1.474749	0.392133
6	8	0	-1.066101	-2.526597	0.982122
7	6	0	-3.741612	-1.314120	1.201245
8	8	0	-3.421969	1.275794	-0.377048
9	6	0	-3.071190	2.645425	-0.203452
10	8	0	0.977550	-0.802335	0.037378
11	6	0	-0.319480	-1.607761	-1.867552
12	6	0	1.953501	-0.049609	-0.525169
13	6	0	3.276561	-0.274914	0.181050
14	8	0	1.774647	0.731656	-1.451555

15	6	0	4.373953	-0.454942	-0.866876
16	6	0	3.594895	0.898794	1.114475
17	6	0	2.665880	0.963856	2.319432
18	8	0	-0.364510	1.639706	-0.027928
19	1	0	-1.095069	0.879721	-1.797086
20	1	0	-3.516443	-1.483653	2.258847
21	1	0	-4.035759	-2.269191	0.753219
22	1	0	-4.601295	-0.639865	1.145984
23	1	0	-3.991732	3.234744	-0.254711
24	1	0	-2.616847	2.816968	0.778429
25	1	0	-2.412508	2.986552	-1.008263
26	1	0	0.124120	-2.595871	-1.698314
27	1	0	0.281856	-1.120218	-2.641594
28	1	0	-1.329636	-1.755085	-2.264372
29	1	0	3.216744	-1.206372	0.760033
30	1	0	4.511447	0.453151	-1.464174
31	1	0	4.122969	-1.269396	-1.556492
32	1	0	5.330081	-0.696629	-0.390907
33	1	0	4.624667	0.805562	1.481010
34	1	0	3.534039	1.848374	0.568530
35	1	0	2.699418	0.031307	2.891370
36	1	0	2.968932	1.779203	2.983472
37	1	0	1.630495	1.146609	2.018375
38	1	0	0.421074	1.868782	-0.564859

7e

Standard orientation:

Center Number	Atomic Number	Atomic Type	Coordinates (Angstroms) X	Y	Z

1	6	0	0.388085	-0.790034	0.474624
2	6	0	1.043309	0.603147	0.669111
3	6	0	2.440811	0.435338	0.129860
4	6	0	2.663716	-0.709989	-0.521880
5	6	0	1.394019	-1.455383	-0.455469
6	8	0	1.222956	-2.511330	-1.053040
7	6	0	3.877562	-1.240860	-1.175136
8	8	0	3.445963	1.339457	0.391256
9	6	0	3.071916	2.701378	0.205141
10	8	0	-0.891207	-0.830566	-0.181878
11	6	0	0.353702	-1.612933	1.766901
12	6	0	-1.901653	-0.098403	0.347758
13	6	0	-3.198882	-0.379640	-0.385710
14	8	0	-1.773900	0.674153	1.289451
15	6	0	-3.937260	-1.489771	0.357402
16	6	0	-4.067768	0.881410	-0.495208
17	6	0	-3.448845	1.940697	-1.398612
18	8	0	0.394214	1.636621	-0.067693
19	1	0	1.077891	0.892143	1.726726
20	1	0	3.694195	-1.414164	-2.240120
21	1	0	4.175817	-2.189418	-0.717896
22	1	0	4.720179	-0.548204	-1.089337
23	1	0	3.976536	3.311138	0.289930
24	1	0	2.648967	2.863552	-0.792751
25	1	0	2.376751	3.026617	0.985704
26	1	0	-0.065424	-2.608445	1.581050
27	1	0	-0.284232	-1.139493	2.520966
28	1	0	1.351928	-1.741857	2.199726
29	1	0	-2.960421	-0.726051	-1.398962

30	1	0	-4.195947	-1.181653	1.376787
31	1	0	-3.316717	-2.388754	0.433542
32	1	0	-4.862243	-1.760362	-0.162330
33	1	0	-5.049231	0.612344	-0.903398
34	1	0	-4.243763	1.316042	0.496139
35	1	0	-3.276733	1.545184	-2.404402
36	1	0	-4.120566	2.800764	-1.483935
37	1	0	-2.494360	2.299641	-1.001520
38	1	0	-0.409123	1.854555	0.446603

7f

Standard orientation:

Center Number	Atomic Number	Atomic Type	Coordinates (Angstroms) X	Y	Z
1	6	0	-0.407690	0.468193	1.043098
2	6	0	-1.489495	-0.640428	0.920505
3	6	0	-2.489138	-0.070878	-0.060706
4	6	0	-2.115198	1.067186	-0.657413
5	6	0	-0.793768	1.407805	-0.099261
6	8	0	-0.123252	2.352431	-0.492575
7	6	0	-2.802808	1.915801	-1.654473
8	8	0	-3.736446	-0.619359	-0.265564
9	6	0	-3.827570	-2.033538	-0.122527
10	8	0	0.957198	0.032864	1.046288
11	6	0	-0.602695	1.258136	2.344968
12	6	0	1.460612	-0.482211	-0.108206
13	6	0	2.956264	-0.701640	0.022409
14	8	0	0.792015	-0.732276	-1.102930

15	6	0	3.396867	-1.839462	-0.895800
16	6	0	3.676899	0.620936	-0.273395
17	6	0	5.174711	0.563123	-0.012456
18	8	0	-0.994295	-1.887037	0.441123
19	1	0	-1.969442	-0.829132	1.887295
20	1	0	-2.205791	1.991343	-2.568518
21	1	0	-2.956716	2.924484	-1.258478
22	1	0	-3.781246	1.509661	-1.926106
23	1	0	-4.852024	-2.327786	-0.371388
24	1	0	-3.155342	-2.541675	-0.821374
25	1	0	-3.634770	-2.341498	0.908942
26	1	0	0.113446	2.086534	2.405749
27	1	0	-0.416992	0.618208	3.215014
28	1	0	-1.610569	1.679298	2.430896
29	1	0	3.155555	-1.016071	1.056110
30	1	0	3.298820	-1.561435	-1.951801
31	1	0	2.782260	-2.731741	-0.732358
32	1	0	4.439144	-2.117865	-0.711776
33	1	0	3.501316	0.920911	-1.313571
34	1	0	3.248179	1.416811	0.349985
35	1	0	5.674800	-0.125537	-0.699622
36	1	0	5.617997	1.553972	-0.154853
37	1	0	5.382322	0.243778	1.013719
38	1	0	-0.614653	-1.723376	-0.449058

7g

Standard orientation:

Center	Atomic	Atomic	Coordinates (Angstroms)		
Number	Number	Type	X	Y	Z

1	6	0	-0.521917	0.339082	1.158548
2	6	0	-1.433218	-0.843277	0.722714
3	6	0	-2.454423	-0.201716	-0.189473
4	6	0	-2.214625	1.077512	-0.502162
5	6	0	-0.981033	1.450143	0.214740
6	8	0	-0.431852	2.535513	0.082789
7	6	0	-2.964323	2.030479	-1.347085
8	8	0	-3.601459	-0.844114	-0.600657
9	6	0	-3.501875	-2.255590	-0.765016
10	8	0	0.887395	0.084655	1.175551
11	6	0	-0.887323	0.793174	2.579512
12	6	0	1.512466	-0.096995	-0.019582
13	6	0	3.010614	-0.218129	0.182262
14	8	0	0.933775	-0.198766	-1.094508
15	6	0	3.345577	-1.674668	0.488647
16	6	0	3.747064	0.320435	-1.053816
17	6	0	5.252616	0.426996	-0.856566
18	8	0	-0.746384	-1.882078	0.029899
19	1	0	-1.926383	-1.297412	1.588990
20	1	0	-2.340815	2.378024	-2.177003
21	1	0	-3.276290	2.900188	-0.761350
22	1	0	-3.863701	1.573501	-1.773585
23	1	0	-4.462557	-2.613788	-1.147548
24	1	0	-2.733389	-2.510327	-1.501129
25	1	0	-3.318429	-2.755402	0.191068
26	1	0	-0.296267	1.670242	2.869165
27	1	0	-0.655066	0.005595	3.306617
28	1	0	-1.946037	1.055384	2.675092
29	1	0	3.282979	0.408841	1.041252

30	1	0	3.133983	-2.318806	-0.372357
31	1	0	2.746982	-2.046481	1.327857
32	1	0	4.399908	-1.794102	0.755754
33	1	0	3.543512	-0.314589	-1.924631
34	1	0	3.358406	1.316335	-1.300922
35	1	0	5.710935	-0.556557	-0.723954
36	1	0	5.713825	0.891770	-1.735076
37	1	0	5.492617	1.041989	0.015731
38	1	0	-0.353613	-1.480764	-0.774896

Table S7 Gibbs free energies and Boltzmann-population of low-energy conformers of 2*S*, 3*R* -**7**.

Conformers of Compound	In MeOH	
	ΔG[a]	P(%)[b]
**7a**	1.27	4.19%
**7b**	1.27	4.20%
**7c**	0.00	36.03%
**7d**	0.01	35.26%
**7e**	1.75	1.87%
**7f**	0.42	17.56%
**7g**	2.19	0.89%

Table S8 Cartesian coordinates for the low-energy optimized conformers of 2*S*, 3*R* -**7** at B3LYP/6-31+G (d, p) level of theory in MeOH.

7a

Standard orientation:

Center	Atomic	Atomic	Coordinates (Angstroms)		
Number	Number	Type	X	Y	Z

1	6	0	-0.337400	-0.400508	1.173269
2	6	0	-1.024520	0.915016	0.725009
3	6	0	-2.097472	0.462615	-0.229494
4	6	0	-2.170416	-0.852633	-0.451206
5	6	0	-1.066593	-1.445384	0.326915
6	8	0	-0.786065	-2.635871	0.278584
7	6	0	-3.092231	-1.653347	-1.283310
8	8	0	-2.991184	1.380897	-0.728436
9	6	0	-2.392537	2.161560	-1.764798
10	8	0	1.091267	-0.409631	0.982634
11	6	0	-0.568958	-0.738664	2.648371
12	6	0	1.572882	-0.275059	-0.280913
13	6	0	3.082598	-0.417273	-0.287350
14	8	0	0.889811	-0.038130	-1.267803
15	6	0	3.475209	-1.461416	-1.330593
16	6	0	3.746638	0.933643	-0.579868
17	6	0	3.539282	1.944695	0.540755
18	8	0	-1.637176	1.630538	1.797476
19	1	0	-0.324151	1.600284	0.239227
20	1	0	-3.632456	-2.378364	-0.666471
21	1	0	-3.831853	-1.023373	-1.787101
22	1	0	-2.538122	-2.199020	-2.052836
23	1	0	-1.983285	1.522150	-2.555367
24	1	0	-1.613477	2.815741	-1.360449
25	1	0	-3.170964	2.792497	-2.202490
26	1	0	-0.108908	0.014374	3.298188
27	1	0	-1.633569	-0.815715	2.892023
28	1	0	-0.100349	-1.696465	2.902557
29	1	0	3.412533	-0.784214	0.692666
30	1	0	3.198157	-1.141624	-2.341871

31	1	0	2.970776	-2.414374	-1.135895
32	1	0	4.555630	-1.639735	-1.315794
33	1	0	4.824735	0.787978	-0.716773
34	1	0	3.359824	1.356157	-1.514691
35	1	0	3.916654	1.558464	1.493137
36	1	0	4.075756	2.871700	0.314232
37	1	0	2.481340	2.194349	0.663771
38	1	0	-2.390386	2.117297	1.413575

7b

Standard orientation:

Center Number	Atomic Number	Atomic Type	Coordinates (Angstroms) X	Y	Z
1	6	0	-0.418601	-0.808310	0.997931
2	6	0	-1.130960	0.567804	1.030590
3	6	0	-2.100754	0.509402	-0.117992
4	6	0	-2.094882	-0.618454	-0.835341
5	6	0	-1.029515	-1.452504	-0.247764
6	8	0	-0.691137	-2.527956	-0.725384
7	6	0	-2.908179	-1.044255	-1.992463
8	8	0	-2.992108	1.538726	-0.305348
9	6	0	-2.350296	2.663878	-0.907702
10	8	0	1.018405	-0.726457	0.946891
11	6	0	-0.750431	-1.695913	2.200693
12	6	0	1.590323	-0.102864	-0.117503
13	6	0	3.098302	-0.255742	-0.064594
14	8	0	0.974997	0.489170	-0.993588
15	6	0	3.486995	-1.462271	-0.913575

16	6	0	3.807276	1.014868	-0.553645
17	6	0	3.591502	2.200338	0.379100
18	8	0	-1.860948	0.803055	2.234185
19	1	0	-0.426627	1.397112	0.914560
20	1	0	-3.462711	-1.958268	-1.755015
21	1	0	-3.633653	-0.277744	-2.280026
22	1	0	-2.269066	-1.242936	-2.858599
23	1	0	-1.856114	2.382933	-1.843927
24	1	0	-1.632298	3.118902	-0.217730
25	1	0	-3.120419	3.406949	-1.136121
26	1	0	-0.378925	-1.249196	3.129718
27	1	0	-1.826167	-1.875675	2.299085
28	1	0	-0.257510	-2.671619	2.107917
29	1	0	3.397421	-0.440710	0.975089
30	1	0	3.208423	-1.315837	-1.963602
31	1	0	2.979942	-2.367055	-0.562063
32	1	0	4.566089	-1.640550	-0.868933
33	1	0	4.885155	0.825372	-0.625856
34	1	0	3.463406	1.285618	-1.559199
35	1	0	3.944306	1.972341	1.389794
36	1	0	4.147073	3.071111	0.015450
37	1	0	2.535362	2.478016	0.437845
38	1	0	-2.602043	1.393067	2.001159

7c

Standard orientation:

Center	Atomic	Atomic	Coordinates (Angstroms)		
Number	Number	Type	X	Y	Z

1	6	0	0.265275	0.499076	0.393675
2	6	0	1.093924	-0.779347	0.102777
3	6	0	2.475173	-0.273049	-0.233464
4	6	0	2.550385	1.050919	-0.405505
5	6	0	1.197544	1.572768	-0.145659
6	8	0	0.894792	2.742614	-0.343680
7	6	0	3.671469	1.912349	-0.838052
8	8	0	3.532852	-1.097503	-0.547614
9	6	0	3.614918	-2.315604	0.184667
10	8	0	-0.972340	0.554520	-0.338249
11	6	0	0.027748	0.819777	1.876192
12	6	0	-1.846097	-0.468319	-0.140526
13	6	0	-3.135814	-0.219779	-0.897306
14	8	0	-1.616896	-1.451371	0.552934
15	6	0	-3.394609	-1.403254	-1.827673
16	6	0	-4.300836	-0.017378	0.078982
17	6	0	-4.170221	1.265522	0.889822
18	8	0	1.099917	-1.659367	1.221039
19	1	0	0.708569	-1.330088	-0.763976
20	1	0	3.898593	2.657529	-0.069722
21	1	0	4.582045	1.332458	-1.021295
22	1	0	3.418536	2.437740	-1.764689
23	1	0	2.811585	-3.000143	-0.102476
24	1	0	3.619790	-2.129116	1.263105
25	1	0	4.564434	-2.795100	-0.074329
26	1	0	-0.599488	0.067310	2.363769
27	1	0	0.968790	0.893422	2.432734
28	1	0	-0.499569	1.774912	1.981532
29	1	0	-3.020966	0.677786	-1.518679
30	1	0	-3.544781	-2.331226	-1.264124

31	1	0	-2.545887	-1.559933	-2.501513
32	1	0	-4.285826	-1.229000	-2.439870
33	1	0	-5.243224	0.022594	-0.480765
34	1	0	-4.374989	-0.867040	0.768284
35	1	0	-4.097287	2.138807	0.234395
36	1	0	-5.047109	1.395632	1.532201
37	1	0	-3.286049	1.242096	1.534505
38	1	0	0.173076	-1.971881	1.307899

7d

Standard orientation:

Center Number	Atomic Number	Atomic Type	Coordinates (Angstroms) X	Y	Z
1	6	0	-0.303143	0.523088	-0.460726
2	6	0	-1.057230	-0.772386	-0.064718
3	6	0	-2.465978	-0.321483	0.232303
4	6	0	-2.617718	1.005602	0.296862
5	6	0	-1.296877	1.581026	-0.008526
6	8	0	-1.062457	2.778872	0.091634
7	6	0	-3.787714	1.834758	0.660312
8	8	0	-3.475793	-1.175778	0.615464
9	6	0	-3.486007	-2.453071	-0.012283
10	8	0	0.926891	0.707997	0.262206
11	6	0	-0.079293	0.734680	-1.963665
12	6	0	1.860249	-0.273718	0.141943
13	6	0	3.121275	0.099462	0.898512
14	8	0	1.687049	-1.323144	-0.464720
15	6	0	3.087418	-0.590133	2.258400

16	6	0	4.377067	-0.293805	0.108321
17	6	0	4.536718	0.512519	-1.174396
18	8	0	-1.011004	-1.738861	-1.107876
19	1	0	-0.644237	-1.228654	0.843074
20	1	0	-4.055739	2.499497	-0.166229
21	1	0	-4.663779	1.220284	0.892470
22	1	0	-3.567123	2.447219	1.539567
23	1	0	-2.643865	-3.063403	0.327688
24	1	0	-3.499480	-2.357288	-1.103080
25	1	0	-4.406474	-2.963948	0.287529
26	1	0	0.589248	-0.019807	-2.389884
27	1	0	-1.021146	0.711792	-2.522672
28	1	0	0.395964	1.705769	-2.147656
29	1	0	3.132295	1.186612	1.051355
30	1	0	3.081204	-1.681270	2.151502
31	1	0	2.186986	-0.309956	2.816122
32	1	0	3.959017	-0.310539	2.859347
33	1	0	5.265092	-0.129419	0.732205
34	1	0	4.356949	-1.361647	-0.141534
35	1	0	4.580772	1.585254	-0.959528
36	1	0	5.464294	0.230768	-1.681901
37	1	0	3.708051	0.332997	-1.866026
38	1	0	-0.068115	-2.006172	-1.170053

7e

Standard orientation:

Center Number	Atomic Number	Atomic Type	Coordinates (Angstroms) X	Y	Z

1	6	0	0.193576	-0.051238	1.119526
2	6	0	1.280042	-1.009511	0.567040
3	6	0	2.230930	-0.104948	-0.169053
4	6	0	1.919545	1.194325	-0.177039
5	6	0	0.635189	1.294383	0.542712
6	8	0	0.015398	2.345226	0.646985
7	6	0	2.613239	2.361940	-0.756426
8	8	0	3.393513	-0.624435	-0.688446
9	6	0	3.135270	-1.331355	-1.902213
10	8	0	-1.151139	-0.413526	0.752140
11	6	0	0.197846	0.062471	2.645639
12	6	0	-1.474487	-0.449119	-0.566638
13	6	0	-2.951549	-0.726321	-0.770016
14	8	0	-0.678079	-0.303786	-1.483829
15	6	0	-3.284726	-2.127040	-0.259948
16	6	0	-3.835958	0.335337	-0.102367
17	6	0	-3.657641	1.716614	-0.718436
18	8	0	2.001515	-1.699273	1.587049
19	1	0	0.858014	-1.769184	-0.097832
20	1	0	2.864094	3.085900	0.024957
21	1	0	3.542751	2.071617	-1.255856
22	1	0	1.976587	2.858165	-1.495503
23	1	0	2.618376	-0.695190	-2.629086
24	1	0	2.556182	-2.240445	-1.709557
25	1	0	4.098144	-1.626987	-2.330146
26	1	0	-0.059466	-0.896061	3.109489
27	1	0	1.167348	0.395426	3.033308
28	1	0	-0.558537	0.782548	2.981657
29	1	0	-3.136000	-0.709339	-1.851606
30	1	0	-3.165294	-2.198504	0.826773

31	1	0	-2.625593	-2.874430	-0.713671
32	1	0	-4.318588	-2.393397	-0.503656
33	1	0	-4.888768	0.046144	-0.202867
34	1	0	-3.625539	0.395522	0.972527
35	1	0	-3.877126	1.698939	-1.789902
36	1	0	-4.338853	2.430270	-0.244328
37	1	0	-2.637298	2.087759	-0.581498
38	1	0	2.892263	-1.875641	1.228915

7f

Standard orientation:

Center Number	Atomic Number	Atomic Type	Coordinates (Angstroms) X	Y	Z
1	6	0	-0.221769	0.544353	-0.659241
2	6	0	-0.975796	-0.750069	-0.259576
3	6	0	-2.276432	-0.268793	0.335848
4	6	0	-2.327723	1.048605	0.556214
5	6	0	-1.047723	1.592262	0.070042
6	8	0	-0.721792	2.760582	0.243392
7	6	0	-3.357207	1.885187	1.210149
8	8	0	-3.253772	-1.113899	0.812781
9	6	0	-3.456535	-2.315057	0.078465
10	8	0	1.126189	0.596023	-0.159869
11	6	0	-0.256357	0.907885	-2.149322
12	6	0	1.958020	-0.411023	-0.535373
13	6	0	3.362963	-0.203875	-0.004999
14	8	0	1.615457	-1.376759	-1.208200
15	6	0	3.968141	1.050744	-0.631559

16	6	0	3.395371	-0.133011	1.527499
17	6	0	2.946472	-1.431437	2.185331
18	8	0	-1.180143	-1.598809	-1.383106
19	1	0	-0.436513	-1.320343	0.506308
20	1	0	-3.724233	2.648200	0.515936
21	1	0	-4.214935	1.291385	1.538401
22	1	0	-2.942199	2.389083	2.088534
23	1	0	-2.607105	-2.995127	0.198263
24	1	0	-3.658517	-2.105200	-0.976352
25	1	0	-4.338275	-2.813633	0.493184
26	1	0	0.276351	0.174109	-2.763067
27	1	0	-1.282376	0.988264	-2.526142
28	1	0	0.240129	1.869638	-2.321740
29	1	0	3.960449	-1.065243	-0.331296
30	1	0	3.442171	1.955858	-0.308970
31	1	0	3.908808	1.006694	-1.724623
32	1	0	5.021832	1.153740	-0.352890
33	1	0	4.417516	0.086606	1.858641
34	1	0	2.762170	0.686156	1.891430
35	1	0	3.563357	-2.272259	1.853162
36	1	0	3.037701	-1.352811	3.272640
37	1	0	1.900500	-1.657593	1.952847
38	1	0	-0.282896	-1.899245	-1.645111

7g

Standard orientation:

Center Number	Atomic Number	Atomic Type	Coordinates (Angstroms) X	Y	Z

1	6	0	-0.398127	-0.812778	0.972542
2	6	0	-1.007862	0.612080	0.993012
3	6	0	-2.078441	0.570817	-0.064801
4	6	0	-2.213821	-0.585962	-0.719391
5	6	0	-1.160152	-1.467277	-0.181248
6	8	0	-0.940407	-2.586890	-0.627118
7	6	0	-3.153386	-1.005531	-1.778669
8	8	0	-2.911472	1.652719	-0.228596
9	6	0	-2.249259	2.700362	-0.938711
10	8	0	1.031092	-0.835626	0.797402
11	6	0	-0.682760	-1.616263	2.244668
12	6	0	1.550561	-0.305389	-0.342553
13	6	0	3.053441	-0.523461	-0.382451
14	8	0	0.899423	0.281183	-1.195149
15	6	0	3.593512	-0.333429	-1.798696
16	6	0	3.767345	0.356553	0.654101
17	6	0	3.654721	1.855812	0.402492
18	8	0	-1.610824	0.956244	2.240326
19	1	0	-0.262709	1.382004	0.773046
20	1	0	-3.745523	-1.864293	-1.447081
21	1	0	-3.847571	-0.202165	-2.043857
22	1	0	-2.609355	-1.289485	-2.684371
23	1	0	-1.857353	2.342516	-1.897251
24	1	0	-1.445221	3.133727	-0.335235
25	1	0	-2.983130	3.485137	-1.140650
26	1	0	-0.200462	-1.153871	3.113275
27	1	0	-1.755634	-1.713032	2.442005
28	1	0	-0.267695	-2.628834	2.161766
29	1	0	3.225892	-1.575992	-0.118315
30	1	0	3.382681	0.667996	-2.188009

31	1	0	3.126812	-1.049715	-2.485120
32	1	0	4.675243	-0.494661	-1.828537
33	1	0	3.365610	0.145146	1.652894
34	1	0	4.829950	0.086033	0.683348
35	1	0	2.611242	2.180536	0.373003
36	1	0	4.152302	2.406031	1.208746
37	1	0	4.135801	2.143059	-0.536747
38	1	0	-2.328002	1.585631	2.040406

Table S9 Gibbs free energies and Boltzmann-population of low-energy conformers of 2*S*, 3*S* -**8**.

Conformers of Compound	In MeOH	
	ΔG[a]	P(%)[b]
**8a**	0.00	49.61%
**8b**	0.00	49.58%
**8c**	2.43	0.82%

Table S10 Cartesian coordinates for the low-energy optimized conformers of 2*S*, 3*S* -**8** at B3LYP/6-31+G (d, p) level of theory in MeOH.

8a

Standard orientation:

Center Number	Atomic Number	Atomic Type	Coordinates (Angstroms) X	Y	Z
1	6	0	1.391185	-0.068161	0.100707
2	6	0	-0.016836	0.485542	0.443857
3	6	0	-0.968180	-0.649021	0.131130
4	6	0	-0.377994	-1.782333	-0.262738
5	6	0	1.060428	-1.500222	-0.288327

6	8	0	1.879521	-2.330397	-0.660515
7	8	0	-2.308479	-0.405137	0.295365
8	6	0	-3.191150	-1.507178	0.106536
9	8	0	-0.140670	0.747653	1.850798
10	6	0	-0.375118	1.764415	-0.340101
11	6	0	-0.709748	1.670009	-1.807754
12	8	0	-0.447938	2.847528	0.256159
13	8	0	1.994912	0.549226	-1.042592
14	6	0	2.394339	-0.043607	1.249793
15	1	0	-0.798606	-2.732194	-0.536959
16	1	0	-2.968865	-2.311920	0.813930
17	1	0	-4.211152	-1.158411	0.296353
18	1	0	-3.144213	-1.871116	-0.925689
19	1	0	-0.333931	1.706924	1.923949
20	1	0	-0.158247	0.865046	-2.294891
21	1	0	-1.782922	1.493417	-1.922185
22	1	0	-0.441865	2.609511	-2.298724
23	1	0	2.782860	0.014351	-1.265533
24	1	0	2.592939	0.984908	1.566890
25	1	0	3.356891	-0.465777	0.938039
26	1	0	2.046795	-0.622390	2.112487

8b

Standard orientation:

Center Number	Atomic Number	Atomic Type	Coordinates (Angstroms) X	Y	Z
1	6	0	1.392155	-0.000108	0.116029
2	6	0	-0.045985	0.477735	0.447613

3	6	0	-0.934695	-0.698024	0.102708
4	6	0	-0.281446	-1.802615	-0.273393
5	6	0	1.143091	-1.452648	-0.257109
6	8	0	2.011008	-2.247658	-0.593210
7	8	0	-2.289351	-0.515377	0.228653
8	6	0	-3.115162	-1.653367	0.001876
9	8	0	-0.201248	0.694916	1.859683
10	6	0	-0.489510	1.747231	-0.310196
11	6	0	-0.548993	1.745149	-1.817500
12	8	0	-0.819447	2.758007	0.325567
13	8	0	1.966180	0.626924	-1.037217
14	6	0	2.389302	0.098149	1.265872
15	1	0	-0.648460	-2.771057	-0.558178
16	1	0	-2.881035	-2.454422	0.711096
17	1	0	-4.154895	-1.352490	0.160830
18	1	0	-3.017004	-2.006012	-1.030037
19	1	0	-0.730218	1.516851	1.940677
20	1	0	-0.490195	0.731842	-2.221135
21	1	0	-1.501919	2.177443	-2.135084
22	1	0	0.275360	2.341525	-2.213970
23	1	0	2.783927	0.130460	-1.245807
24	1	0	2.522315	1.140819	1.572749
25	1	0	3.376412	-0.267143	0.961015
26	1	0	2.076039	-0.491319	2.134636

8c

Standard orientation:

Center	Atomic	Atomic	Coordinates (Angstroms)		
Number	Number	Type	X	Y	Z

1	6	0	1.268532	-0.482012	0.220082
2	6	0	0.071222	0.487932	0.399232
3	6	0	-1.145251	-0.373109	0.116289
4	6	0	-0.878624	-1.577328	-0.402028
5	6	0	0.584162	-1.659672	-0.460852
6	8	0	1.171722	-2.590396	-0.997152
7	8	0	-2.366945	0.171058	0.432760
8	6	0	-3.513577	-0.579119	0.040931
9	8	0	-0.055978	0.978593	1.734548
10	6	0	0.148237	1.655878	-0.612172
11	6	0	0.975888	2.851364	-0.204712
12	8	0	-0.435279	1.644976	-1.700369
13	8	0	2.317600	0.017175	-0.607824
14	6	0	1.889920	-0.958164	1.533220
15	1	0	-1.532640	-2.371168	-0.709542
16	1	0	-3.548296	-1.540356	0.563762
17	1	0	-4.403204	-0.007341	0.323100
18	1	0	-3.533157	-0.724518	-1.044485
19	1	0	-0.991156	1.245115	1.834738
20	1	0	1.937461	2.528682	0.201140
21	1	0	1.165171	3.480631	-1.078655
22	1	0	0.431295	3.432556	0.543149
23	1	0	2.827429	-0.761954	-0.909813
24	1	0	2.375185	-0.127523	2.057612
25	1	0	2.669996	-1.705860	1.344349
26	1	0	1.149912	-1.414115	2.198998

Table S11 Gibbs free energies and Boltzmann-population of low-energy conformers of 2*S*, 3*R* -**8**.

Conformers of Compound	In MeOH	
	ΔG[a]	P(%)[b]
**8a**	0.00	100.00%

Table S12 Cartesian coordinates for the low-energy optimized conformers of 2*S*, 3*R* **-8** at B3LYP/6-31+G (d, p) level of theory in MeOH.

8a

Standard orientation:

Center Number	Atomic Number	Atomic Type	Coordinates (Angstroms) X	Y	Z
1	6	0	-1.373055	-0.041665	0.022234
2	6	0	0.052481	0.463661	0.380417
3	6	0	0.958017	-0.713502	0.083989
4	6	0	0.319962	-1.846146	-0.235052
5	6	0	-1.109102	-1.526777	-0.170931
6	8	0	-1.981906	-2.382105	-0.248339
7	8	0	2.310924	-0.508273	0.204085
8	6	0	3.155407	-1.624278	-0.054816
9	8	0	0.132100	0.705826	1.791120
10	6	0	0.483569	1.684429	-0.457794
11	6	0	0.654416	2.991860	0.271510
12	8	0	0.660982	1.624494	-1.678780
13	8	0	-2.268133	0.181373	1.125062
14	6	0	-2.030690	0.498108	-1.244970
15	1	0	0.701397	-2.825286	-0.457622
16	1	0	3.037126	-1.973680	-1.086068
17	1	0	4.191488	-1.300108	0.076814
18	1	0	2.961263	-2.434429	0.655801

19	1	0	-0.795037	0.820769	2.088295
20	1	0	0.905724	3.781473	-0.443636
21	1	0	-0.279851	3.259980	0.771488
22	1	0	1.467103	2.906920	0.997669
23	1	0	-3.021552	-0.429965	0.986125
24	1	0	-1.455863	0.266349	-2.146528
25	1	0	-2.181650	1.581764	-1.179800
26	1	0	-3.027917	0.061558	-1.375238

Table S13 Gibbs free energies and Boltzmann-population of low-energy conformers of 2*R*, 3*R* -**9**.

Conformers of Compound	In MeOH	
	ΔG[a]	P(%)[b]
**9a**	0.02	22.40%
**9b**	1.09	3.66%
**9c**	0.36	12.67%
**9d**	0.00	23.17%
**9e**	0.05	21.22%
**9f**	0.52	9.57%
**9g**	0.68	7.31%

Table S14 Cartesian coordinates for the low-energy optimized conformers of 2*R*, 3*R* -**9** at B3LYP/6-31+G (d, p) level of theory in MeOH.

9a

Standard orientation:

Center Number	Atomic Number	Atomic Type	Coordinates (Angstroms) X	Y	Z
1	6	0	-1.873607	-0.562514	0.344815

2	6	0	-1.012181	-0.798839	-0.902655
3	6	0	-0.273102	0.517304	-1.276697
4	6	0	-1.222601	1.698929	-1.381710
5	6	0	-2.080333	1.841720	-0.147536
6	6	0	-2.384356	0.638565	0.687438
7	6	0	-3.307981	0.880703	1.844163
8	8	0	-2.232127	-1.706612	1.049653
9	8	0	-2.576177	2.931702	0.139758
10	6	0	-1.966814	-1.256155	-2.026177
11	8	0	-0.042403	-1.816147	-0.583758
12	6	0	0.578871	-2.477423	-1.676066
13	8	0	0.702633	0.856733	-0.253332
14	6	0	2.009054	0.578929	-0.509271
15	6	0	2.861958	0.955467	0.689053
16	6	0	3.813359	-0.186174	1.070866
17	6	0	3.077939	-1.400556	1.620643
18	6	0	3.634373	2.226196	0.347801
19	8	0	2.444362	0.124560	-1.557726
20	6	0	-1.349047	-1.944902	2.147854
21	1	0	0.232622	0.405793	-2.243746
22	1	0	-0.642011	2.624018	-1.485800
23	1	0	-1.884523	1.605568	-2.248574
24	1	0	-2.834707	1.543316	2.575839
25	1	0	-3.588910	-0.040456	2.363401
26	1	0	-4.237830	1.348280	1.502247
27	1	0	-2.365988	-2.256115	-1.817298
28	1	0	-2.816442	-0.577256	-2.155720
29	1	0	-1.448836	-1.316856	-2.989323
30	1	0	-0.105048	-3.212487	-2.110318
31	1	0	0.913823	-1.778884	-2.446660

32	1	0	1.449832	-3.017787	-1.294702
33	1	0	2.205004	1.166537	1.543290
34	1	0	4.515685	0.166628	1.837227
35	1	0	4.413291	-0.493535	0.206468
36	1	0	3.795425	-2.166745	1.930721
37	1	0	2.419928	-1.843653	0.867547
38	1	0	2.471763	-1.131980	2.492016
39	1	0	4.318925	2.064567	-0.492399
40	1	0	4.222321	2.567683	1.206051
41	1	0	2.949896	3.033369	0.064921
42	1	0	-1.358762	-1.107989	2.854364
43	1	0	-1.703997	-2.835342	2.673578
44	1	0	-0.324955	-2.136067	1.814984

9b

Standard orientation:

Center	Atomic	Atomic	Coordinates (Angstroms)		
Number	Number	Type	X	Y	Z
1	6	0	1.790646	0.631751	0.299310
2	6	0	0.943102	0.749524	-0.974481
3	6	0	0.304162	-0.631353	-1.298675
4	6	0	1.336360	-1.746994	-1.330419
5	6	0	2.172521	-1.773323	-0.072609
6	6	0	2.373629	-0.513538	0.709196
7	6	0	3.284161	-0.637133	1.896058
8	8	0	2.059899	1.829751	0.954747
9	8	0	2.734971	-2.811437	0.275844
10	6	0	1.887146	1.221074	-2.098647

11	8	0	-0.101701	1.709300	-0.720069
12	6	0	-0.742018	2.276047	-1.854672
13	8	0	-0.664701	-0.991434	-0.276342
14	6	0	-1.983307	-0.804781	-0.559015
15	6	0	-2.823986	-1.160372	0.656902
16	6	0	-2.577761	-0.179354	1.814516
17	6	0	-3.075051	1.238773	1.564950
18	6	0	-4.300983	-1.281642	0.286061
19	8	0	-2.425680	-0.433539	-1.637225
20	6	0	1.133956	2.066822	2.016922
21	1	0	-0.187247	-0.600721	-2.278126
22	1	0	0.824133	-2.713852	-1.402975
23	1	0	2.010775	-1.646062	-2.186331
24	1	0	2.842312	-1.302588	2.644192
25	1	0	3.481680	0.323043	2.381781
26	1	0	4.253441	-1.047008	1.594330
27	1	0	2.211649	2.255566	-1.931267
28	1	0	2.784828	0.598363	-2.180596
29	1	0	1.388409	1.200119	-3.074213
30	1	0	-1.010157	1.522880	-2.598725
31	1	0	-1.656794	2.770971	-1.515407
32	1	0	-0.100497	3.036929	-2.307788
33	1	0	-2.496058	-2.158813	0.978586
34	1	0	-1.505238	-0.135177	2.038864
35	1	0	-3.066510	-0.564237	2.718923
36	1	0	-2.795084	1.883888	2.404161
37	1	0	-4.164494	1.271189	1.473727
38	1	0	-2.637742	1.663495	0.657759
39	1	0	-4.692938	-0.355558	-0.147310
40	1	0	-4.904444	-1.535865	1.162859

41	1	0	-4.444039	-2.070705	-0.461388
42	1	0	1.130451	1.241608	2.736947
43	1	0	1.454476	2.971022	2.541716
44	1	0	0.121323	2.237394	1.642259

9c

Standard orientation:

Center Number	Atomic Number	Atomic Type	Coordinates (Angstroms) X	Y	Z
1	6	0	-1.947986	-0.034089	0.346717
2	6	0	-1.214604	-0.905418	-0.681739
3	6	0	-0.052204	-0.091631	-1.318376
4	6	0	-0.508269	1.261168	-1.836014
5	6	0	-1.254420	2.042702	-0.781201
6	6	0	-1.987892	1.313532	0.298807
7	6	0	-2.773085	2.183593	1.236378
8	8	0	-2.714360	-0.726556	1.279309
9	8	0	-1.309994	3.270835	-0.844082
10	6	0	-2.256121	-1.329763	-1.736856
11	8	0	-0.668862	-2.042806	0.016913
12	6	0	-0.303867	-3.166862	-0.770481
13	8	0	0.973861	0.164521	-0.322158
14	6	0	2.103391	-0.588816	-0.371544
15	6	0	3.003591	-0.301928	0.815477
16	6	0	3.358280	1.186834	0.934205
17	6	0	4.149450	1.698931	-0.261465
18	6	0	2.335870	-0.815983	2.089207
19	8	0	2.371662	-1.404286	-1.244620

20	6	0	-1.998686	-0.931159	2.498963
21	1	0	0.378830	-0.645229	-2.159841
22	1	0	0.368003	1.854197	-2.125625
23	1	0	-1.160720	1.157523	-2.709236
24	1	0	-2.098209	2.821019	1.816911
25	1	0	-3.377061	1.609182	1.943469
26	1	0	-3.462940	2.825163	0.676952
27	1	0	-2.988560	-2.024874	-1.307172
28	1	0	-2.805889	-0.475917	-2.147283
29	1	0	-1.783470	-1.847951	-2.578543
30	1	0	0.265394	-2.883554	-1.658779
31	1	0	0.317905	-3.822617	-0.155508
32	1	0	-1.195479	-3.728158	-1.062291
33	1	0	3.929169	-0.871908	0.662270
34	1	0	3.958251	1.343940	1.839315
35	1	0	2.451939	1.794703	1.048025
36	1	0	4.427600	2.747046	-0.107851
37	1	0	3.564480	1.640857	-1.183948
38	1	0	5.070332	1.122477	-0.399505
39	1	0	1.422105	-0.255994	2.318089
40	1	0	3.011888	-0.725001	2.945992
41	1	0	2.057909	-1.870560	1.985375
42	1	0	-1.671935	0.018711	2.935369
43	1	0	-2.680035	-1.411859	3.207988
44	1	0	-1.138658	-1.591526	2.360465

9d

Standard orientation:

Center Atomic Atomic Coordinates (Angstroms)

Number	Number	Type	X	Y	Z
1	6	0	-2.353891	-0.356448	-0.403105
2	6	0	-0.971903	-0.826623	0.075244
3	6	0	0.016737	0.363262	-0.105541
4	6	0	-0.475172	1.579094	0.664673
5	6	0	-1.850626	1.975812	0.192529
6	6	0	-2.748185	0.933697	-0.398511
7	6	0	-4.065969	1.429677	-0.912314
8	8	0	-3.187855	-1.331000	-0.941384
9	8	0	-2.252115	3.132019	0.323845
10	6	0	-0.483284	-2.032845	-0.750994
11	8	0	-1.198810	-1.199388	1.449814
12	6	0	-0.095124	-1.668959	2.204030
13	8	0	1.346020	0.006012	0.339190
14	6	0	2.377134	0.680998	-0.239155
15	6	0	3.700564	0.191547	0.317292
16	6	0	4.499596	-0.547077	-0.762176
17	6	0	3.837150	-1.848665	-1.195643
18	6	0	4.480213	1.387636	0.859310
19	8	0	2.256727	1.547344	-1.093217
20	6	0	-3.891626	-2.027878	0.089122
21	1	0	0.057361	0.598307	-1.179914
22	1	0	-0.503047	1.395595	1.743889
23	1	0	0.204022	2.423954	0.501431
24	1	0	-4.651163	1.871872	-0.100153
25	1	0	-4.671674	0.635304	-1.359273
26	1	0	-3.911707	2.191011	-1.684607
27	1	0	-1.083236	-2.927094	-0.549669
28	1	0	-0.521019	-1.823089	-1.826905

29	1	0	0.549835	-2.300060	-0.509642
30	1	0	0.570139	-0.846295	2.474531
31	1	0	-0.485410	-2.087614	3.136847
32	1	0	0.454675	-2.460085	1.689228
33	1	0	3.506201	-0.489119	1.156717
34	1	0	5.500852	-0.780064	-0.380231
35	1	0	4.633600	0.092243	-1.643772
36	1	0	4.471587	-2.371761	-1.917941
37	1	0	2.869930	-1.665447	-1.674137
38	1	0	3.678705	-2.513312	-0.339623
39	1	0	4.730564	2.097241	0.062096
40	1	0	5.413743	1.063188	1.330233
41	1	0	3.891694	1.927616	1.609527
42	1	0	-4.409407	-1.333548	0.759846
43	1	0	-4.643544	-2.661884	-0.389856
44	1	0	-3.218104	-2.676226	0.656827

9e

Standard orientation:

Center Number	Atomic Number	Atomic Type	Coordinates (Angstroms) X	Y	Z
1	6	0	-2.126842	-0.336907	0.316225
2	6	0	-1.186984	-0.976604	-0.715038
3	6	0	-0.126337	0.058974	-1.179221
4	6	0	-0.745093	1.386000	-1.587577
5	6	0	-1.641636	1.933562	-0.505767
6	6	0	-2.354033	0.989239	0.410109
7	6	0	-3.332507	1.629957	1.349075

8	8	0	-2.866956	-1.230559	1.083982
9	8	0	-1.837929	3.145708	-0.413699
10	6	0	-2.061459	-1.452486	-1.891497
11	8	0	-0.536049	-2.096164	-0.078038
12	6	0	0.251353	-2.943262	-0.900370
13	8	0	0.808638	0.305061	-0.095841
14	6	0	2.106910	0.513394	-0.442055
15	6	0	2.947835	0.744741	0.798523
16	6	0	4.188751	-0.157645	0.796114
17	6	0	3.837940	-1.634371	0.924295
18	6	0	3.335995	2.219395	0.850561
19	8	0	2.532531	0.526226	-1.588519
20	6	0	-2.245658	-1.482155	2.346384
21	1	0	0.414052	-0.355120	-2.038306
22	1	0	0.044716	2.124684	-1.765468
23	1	0	-1.337226	1.286325	-2.502922
24	1	0	-2.807465	2.266835	2.068707
25	1	0	-3.916702	0.899571	1.917546
26	1	0	-4.048984	2.246610	0.796028
27	1	0	-2.685307	-2.305629	-1.596805
28	1	0	-2.721496	-0.663313	-2.267467
29	1	0	-1.446752	-1.789903	-2.734012
30	1	0	1.028963	-2.388707	-1.429789
31	1	0	0.743815	-3.674640	-0.251907
32	1	0	-0.373950	-3.496055	-1.607102
33	1	0	2.347143	0.512739	1.687242
34	1	0	4.840698	0.116667	1.633975
35	1	0	4.771030	-0.008256	-0.121884
36	1	0	4.751598	-2.235189	0.970755
37	1	0	3.251941	-1.981545	0.067645

38	1	0	3.260579	-1.822692	1.835210
39	1	0	3.952141	2.500753	-0.010697
40	1	0	3.900236	2.443185	1.761505
41	1	0	2.444561	2.856222	0.839178
42	1	0	-2.110126	-0.556327	2.916092
43	1	0	-2.908836	-2.137392	2.918515
44	1	0	-1.285903	-1.991976	2.232305

9f

Standard orientation:

Center	Atomic	Atomic	Coordinates (Angstroms)		
Number	Number	Type	X	Y	Z
1	6	0	-1.994473	0.151343	0.319467
2	6	0	-1.385336	-0.952351	-0.556999
3	6	0	-0.076057	-0.439804	-1.218195
4	6	0	-0.254551	0.910947	-1.890736
5	6	0	-0.839274	1.925225	-0.939379
6	6	0	-1.763517	1.468989	0.143663
7	6	0	-2.413144	2.567936	0.932242
8	8	0	-2.936382	-0.277718	1.249530
9	8	0	-0.608454	3.125022	-1.088455
10	6	0	-2.440425	-1.327431	-1.616057
11	8	0	-1.104851	-2.081443	0.296868
12	6	0	-0.640976	-3.269250	-0.325690
13	8	0	0.950748	-0.316700	-0.198502
14	6	0	2.232953	-0.541037	-0.587306
15	6	0	3.194239	-0.393307	0.576778
16	6	0	3.036222	0.952096	1.301191

17	6	0	3.351168	2.142593	0.403624
18	6	0	2.999622	-1.564158	1.538048
19	8	0	2.576050	-0.843511	-1.721544
20	6	0	-2.351253	-0.473258	2.538995
21	1	0	0.248822	-1.169464	-1.969511
22	1	0	0.717719	1.294349	-2.222010
23	1	0	-0.912189	0.841896	-2.763629
24	1	0	-1.660921	3.112039	1.513485
25	1	0	-3.170034	2.200356	1.630104
26	1	0	-2.914739	3.275758	0.264406
27	1	0	-3.284737	-1.856249	-1.156819
28	1	0	-2.834237	-0.451547	-2.142061
29	1	0	-2.022399	-2.000460	-2.373521
30	1	0	-0.431964	-3.998646	0.462564
31	1	0	-1.403648	-3.697517	-0.979855
32	1	0	0.287460	-3.101790	-0.877021
33	1	0	4.209277	-0.451681	0.164748
34	1	0	3.714987	0.980056	2.162200
35	1	0	2.020045	1.065056	1.697314
36	1	0	3.279810	3.072089	0.975438
37	1	0	2.649554	2.209142	-0.432996
38	1	0	4.365778	2.070470	-0.000674
39	1	0	2.008330	-1.541206	2.004543
40	1	0	3.750506	-1.540924	2.335934
41	1	0	3.093615	-2.520630	1.011731
42	1	0	-1.616111	-1.282248	2.533722
43	1	0	-1.889783	0.446713	2.911926
44	1	0	-3.153222	-0.751962	3.228919

9g

Standard orientation:

Center Number	Atomic Number	Atomic Type	Coordinates (Angstroms) X	Y	Z
1	6	0	-2.396953	-0.274538	-0.297766
2	6	0	-0.998682	-0.868128	-0.070424
3	6	0	0.042673	0.246046	-0.388155
4	6	0	-0.211367	1.469087	0.479711
5	6	0	-1.609639	1.988536	0.256515
6	6	0	-2.675658	1.041754	-0.197405
7	6	0	-4.015737	1.657323	-0.470184
8	8	0	-3.386117	-1.161095	-0.708704
9	8	0	-1.885038	3.166545	0.480278
10	6	0	-0.755720	-2.077381	-0.995151
11	8	0	-1.024781	-1.274259	1.312274
12	6	0	0.151301	-1.843569	1.862497
13	8	0	1.392117	-0.227925	-0.180207
14	6	0	2.364568	0.372399	-0.918326
15	6	0	3.732859	-0.198685	-0.599208
16	6	0	4.113045	0.006204	0.872913
17	6	0	4.224517	1.477510	1.251176
18	6	0	3.775646	-1.677953	-0.981353
19	8	0	2.172717	1.262091	-1.735435
20	6	0	-3.963678	-1.838917	0.409916
21	1	0	-0.077529	0.515530	-1.449424
22	1	0	-0.072980	1.251122	1.543810
23	1	0	0.498328	2.262962	0.220966
24	1	0	-4.418411	2.114409	0.440746
25	1	0	-4.751450	0.929101	-0.824366

26	1	0	-3.928685	2.431857	-1.239187
27	1	0	-1.380699	-2.931762	-0.712484
28	1	0	-0.960524	-1.828419	-2.043066
29	1	0	0.280077	-2.429739	-0.942995
30	1	0	0.560368	-2.640626	1.237469
31	1	0	0.902809	-1.074314	2.052256
32	1	0	-0.113392	-2.281303	2.829576
33	1	0	4.457506	0.332079	-1.229885
34	1	0	5.079279	-0.474154	1.067342
35	1	0	3.381599	-0.475438	1.533235
36	1	0	4.552061	1.574668	2.290571
37	1	0	3.261157	1.989019	1.156001
38	1	0	4.952892	1.992197	0.616419
39	1	0	3.107516	-2.276169	-0.350890
40	1	0	4.788736	-2.077151	-0.872239
41	1	0	3.463786	-1.820674	-2.021733
42	1	0	-4.300539	-1.132314	1.175988
43	1	0	-4.835321	-2.392571	0.049787
44	1	0	-3.260817	-2.559309	0.837919

Table S15 Gibbs free energies and Boltzmann-population of low-energy conformers of 2*S*, 3*R*, 4*R* -**10**.

Conformers of Compound	In MeOH	
	ΔG[a]	P(%)[b]
**10a**	0.00	100.00%
**10b**	7.03	0.00%

Table S16 Cartesian coordinates for the low-energy optimized conformers of 2*S*, 3*R*, 4*R* -**10** at B3LYP/6-31+G (d, p) level of theory in MeOH.

10a

Standard orientation:

Center Number	Atomic Number	Atomic Type	Coordinates (Angstroms) X	Y	Z
1	6	0	0.873235	1.017720	-0.363246
2	6	0	1.525659	-0.286797	0.114455
3	6	0	0.627705	-1.448628	-0.302024
4	6	0	-0.840564	-1.283150	-0.190930
5	6	0	-1.388778	-0.081789	0.065933
6	6	0	-0.560142	1.197150	0.178499
7	6	0	-1.229379	2.378832	-0.544199
8	8	0	1.689332	2.134484	0.020089
9	6	0	1.784930	-0.347535	1.620567
10	8	0	2.803914	-0.392325	-0.535662
11	8	0	1.093008	-2.524005	-0.680718
12	8	0	-2.738872	0.138284	0.260757
13	6	0	-3.634446	-0.964895	0.173758
14	1	0	0.852608	1.022995	-1.462638
15	1	0	-1.406059	-2.198829	-0.303008
16	1	0	-0.507768	1.470831	1.240647
17	1	0	-1.374690	2.162749	-1.608828
18	1	0	-2.209598	2.607182	-0.110430
19	1	0	-0.623467	3.286815	-0.462160
20	1	0	2.597522	1.894587	-0.254293
21	1	0	2.277478	-1.290869	1.885628
22	1	0	0.863997	-0.282532	2.207085
23	1	0	2.461111	0.453238	1.939511
24	1	0	3.005886	-1.350063	-0.573962
25	1	0	-3.611244	-1.408609	-0.826433

26	1	0	-3.407909	-1.713264	0.940863
27	1	0	-4.646262	-0.591185	0.355421

10b

Standard orientation:

Center Number	Atomic Number	Atomic Type	Coordinates (Angstroms) X	Y	Z
1	6	0	0.646564	1.100206	-0.354452
2	6	0	1.518870	-0.045216	0.186757
3	6	0	0.983045	-1.363925	-0.371021
4	6	0	-0.482203	-1.540949	-0.465957
5	6	0	-1.310150	-0.511165	-0.245739
6	6	0	-0.850964	0.922069	-0.019106
7	6	0	-1.675354	1.927095	-0.838972
8	8	0	1.116991	2.352140	0.165761
9	6	0	1.580610	-0.118839	1.713574
10	8	0	2.860285	0.180131	-0.274767
11	8	0	1.736147	-2.282326	-0.694390
12	8	0	-2.676964	-0.731458	-0.325558
13	6	0	-3.296873	-0.662498	0.959005
14	1	0	0.774652	1.158287	-1.443801
15	1	0	-0.842042	-2.537327	-0.693768
16	1	0	-1.005124	1.171329	1.039530
17	1	0	-1.602103	1.718495	-1.912455
18	1	0	-2.734839	1.895971	-0.563447
19	1	0	-1.328740	2.951749	-0.668559
20	1	0	2.084383	2.345088	0.014100
21	1	0	2.237589	-0.938001	2.030874

22	1	0	0.598912	-0.293073	2.165248
23	1	0	2.003692	0.797179	2.138435
24	1	0	3.280255	-0.703956	-0.317235
25	1	0	-2.826791	-1.360989	1.659696
26	1	0	-3.266393	0.355785	1.358223
27	1	0	-4.346396	-0.949102	0.840257

Table S17 Gibbs free energies and Boltzmann-population of low-energy conformers of 2*R*, 3*R*, 4*R* -**11**.

Conformers of Compound	In MeOH	
	ΔG[a]	P(%)[b]
**11a**	0.00	99.97%
**11b**	4.83	0.03%

Table S18 Cartesian coordinates for the low-energy optimized conformers of 7*S*, 10*R*, 11*R*, 13*E*, 14*R*, 16*S* -**1** at B3LYP/6-31+G (d, p) level of theory in MeOH.

11a

Standard orientation:

Center Number	Atomic Number	Atomic Type	Coordinates (Angstroms) X	Y	Z
1	6	0	0.850417	-1.119659	0.544929
2	6	0	1.544081	0.013486	-0.226862
3	6	0	0.728965	1.300971	-0.115221
4	6	0	-0.747711	1.218872	-0.063445
5	6	0	-1.383430	0.043333	0.079491
6	6	0	-0.655685	-1.281944	0.255458
7	6	0	-0.918973	-2.205076	-0.938586
8	8	0	1.016859	-0.869662	1.957373

9	6	0	1.788456	-0.292099	-1.701695
10	8	0	2.835332	0.221090	0.383960
11	8	0	1.276649	2.405513	-0.122135
12	8	0	-2.757012	-0.110514	0.130237
13	6	0	-3.576698	1.051970	0.072281
14	1	0	1.375402	-2.066437	0.368773
15	1	0	-1.251487	2.172393	-0.146887
16	1	0	-1.098798	-1.769178	1.136407
17	1	0	-0.362652	-3.143195	-0.837770
18	1	0	-0.647561	-1.747770	-1.893269
19	1	0	-1.982926	-2.466114	-0.998137
20	1	0	1.954718	-0.601427	2.057831
21	1	0	2.477423	0.441540	-2.138037
22	1	0	0.872189	-0.262932	-2.296521
23	1	0	2.261160	-1.273007	-1.825431
24	1	0	3.087260	1.142064	0.160726
25	1	0	-3.369756	1.717895	0.916852
26	1	0	-3.446098	1.574190	-0.880614
27	1	0	-4.620025	0.731443	0.142490

11b

Standard orientation:

Center Number	Atomic Number	Atomic Type	Coordinates (Angstroms) X	Y	Z
1	6	0	0.532423	-1.103443	0.607057
2	6	0	1.548704	-0.193787	-0.105521
3	6	0	1.012165	1.235094	-0.198327
4	6	0	-0.437685	1.441530	-0.391565

5	6	0	-1.306873	0.432680	-0.253376
6	6	0	-0.923430	-0.991358	0.107877
7	6	0	-1.212776	-1.938743	-1.059773
8	8	0	0.545824	-0.777202	2.014587
9	6	0	1.932752	-0.668821	-1.504984
10	8	0	2.752075	-0.191822	0.689421
11	8	0	1.770491	2.205744	-0.167139
12	8	0	-2.646258	0.676737	-0.520227
13	6	0	-3.453348	0.604395	0.655850
14	1	0	0.870190	-2.146136	0.561486
15	1	0	-0.755512	2.444318	-0.651186
16	1	0	-1.561947	-1.311604	0.941060
17	1	0	-0.893112	-2.959322	-0.822870
18	1	0	-0.715732	-1.633082	-1.983299
19	1	0	-2.288340	-1.977025	-1.269418
20	1	0	1.495846	-0.679705	2.240162
21	1	0	2.806982	-0.117633	-1.871212
22	1	0	1.132846	-0.524286	-2.235271
23	1	0	2.217700	-1.727267	-1.494512
24	1	0	3.218391	0.639205	0.457277
25	1	0	-3.629859	-0.437733	0.940288
26	1	0	-3.004438	1.159287	1.486054
27	1	0	-4.421660	1.058040	0.424870

Table S19 Gibbs free energies and Boltzmann-population of low-energy conformers of 2*R*, 3*R*, 4*R* -**12**.

Conformers of Compound	In MeOH	
	ΔG[a]	P(%)[b]
**12a**	0.00	100.00%

**12b**	7.57	0.00%

Table S20 Cartesian coordinates for the low-energy optimized conformers of 2*R*, 3*R*, 4*R* **-12** at B3LYP/6-31+G (d, p) level of theory in MeOH.

12a

Standard orientation:

Center Number	Atomic Number	Atomic Type	Coordinates (Angstroms) X	Y	Z
1	6	0	0.803820	1.233540	0.021317
2	6	0	1.600219	-0.046156	0.303609
3	6	0	0.858439	-1.246966	-0.283370
4	6	0	-0.621146	-1.263987	-0.249413
5	6	0	-1.324790	-0.185810	0.139667
6	6	0	-0.664943	1.145138	0.493302
7	6	0	-1.465121	2.344577	-0.036899
8	8	0	0.874621	1.522705	-1.385441
9	6	0	1.847323	-0.281943	1.793109
10	8	0	2.881626	0.079935	-0.339076
11	8	0	1.468477	-2.220845	-0.730014
12	8	0	-2.700807	-0.154044	0.277013
13	6	0	-3.452190	-1.302903	-0.098686
14	1	0	1.290072	2.084626	0.514673
15	1	0	-1.065039	-2.209970	-0.529299
16	1	0	-0.670157	1.210564	1.589988
17	1	0	-1.626735	2.279076	-1.118171
18	1	0	-2.448322	2.405462	0.443016
19	1	0	-0.943394	3.283605	0.175999
20	1	0	1.818309	1.404083	-1.623580

21	1	0	2.479338	-1.165733	1.944152
22	1	0	0.919914	-0.439082	2.352707
23	1	0	2.384008	0.564173	2.235545
24	1	0	3.193476	-0.837346	-0.488189
25	1	0	-3.304177	-1.538409	-1.157881
26	1	0	-3.197207	-2.159637	0.533301
27	1	0	-4.511087	-1.076366	0.052068

12b

Standard orientation:

Center Number	Atomic Number	Atomic Type	Coordinates (Angstroms) X	Y	Z
1	6	0	0.608533	1.237475	0.207032
2	6	0	1.524353	0.024246	0.426698
3	6	0	1.079621	-1.130064	-0.476377
4	6	0	-0.364093	-1.318311	-0.728001
5	6	0	-1.259317	-0.421771	-0.293645
6	6	0	-0.893999	0.900915	0.366095
7	6	0	-1.760942	2.059353	-0.145485
8	8	0	0.876714	1.792556	-1.092669
9	6	0	1.547025	-0.449736	1.878032
10	8	0	2.864637	0.413363	0.072201
11	8	0	1.894948	-1.927306	-0.942519
12	8	0	-2.604507	-0.656179	-0.531159
13	6	0	-3.288789	-1.047205	0.658765
14	1	0	0.873750	2.030636	0.916384
15	1	0	-0.654415	-2.219292	-1.254331
16	1	0	-1.094011	0.785994	1.439491

17	1	0	-1.707980	2.155543	-1.236503
18	1	0	-2.812971	1.915991	0.123260
19	1	0	-1.438688	3.008127	0.297144
20	1	0	1.853563	1.816788	-1.165033
21	1	0	2.263225	-1.269809	2.005417
22	1	0	0.570052	-0.808064	2.217801
23	1	0	1.874780	0.355093	2.545291
24	1	0	3.324942	-0.417147	-0.171210
25	1	0	-2.826951	-1.936764	1.100782
26	1	0	-3.314294	-0.230943	1.387296
27	1	0	-4.319684	-1.295017	0.388681
